# Comprehensive dry eye therapy: overcoming ocular surface barrier and combating inflammation, oxidation, and mitochondrial damage

**DOI:** 10.1186/s12951-024-02503-7

**Published:** 2024-05-09

**Authors:** Yuanyou Xia, Yu Zhang, Yangrui Du, Zhigang Wang, Long Cheng, Zhiyu Du

**Affiliations:** 1 Department of Ophthalmology, Second Affiliated Hospital of Chongqing Medical University, Chongqing, 400010 China; 2 Chongqing Key Laboratory of Ultrasound Molecular Imaging, Second Affiliated Hospital of Chongqing Medical University, Chongqing, 400010 China; 3 State Key Laboratory of Ultrasound in Medicine and Engineering, Chongqing Medical University, Chongqing, 400010 China

**Keywords:** Ocular adhesive, Anti-inflammation, Anti-oxidation, Mitochondrial metabolites, Dry eye

## Abstract

**Background:**

Dry Eye Disease (DED) is a prevalent multifactorial ocular disease characterized by a vicious cycle of inflammation, oxidative stress, and mitochondrial dysfunction on the ocular surface, all of which lead to DED deterioration and impair the patients’ quality of life and social functioning. Currently, anti-inflammatory drugs have shown promising efficacy in treating DED; however, such drugs are associated with side effects. The bioavailability of ocular drugs is less than 5% owing to factors such as rapid tear turnover and the presence of the corneal barrier. This calls for investigations to overcome these challenges associated with ocular drug administration.

**Results:**

A novel hierarchical action liposome nanosystem (PHP-DPS@INS) was developed in this study. In terms of delivery, PHP-DPS@INS nanoparticles (NPs) overcame the ocular surface transport barrier by adopting the strategy of “ocular surface electrostatic adhesion-lysosomal site-directed escape”. In terms of therapy, PHP-DPS@INS achieved mitochondrial targeting and antioxidant effects through SS-31 peptide, and exerted an anti-inflammatory effect by loading insulin to reduce mitochondrial inflammatory metabolites. Ultimately, the synergistic action of “anti-inflammation-antioxidation-mitochondrial function restoration” breaks the vicious cycle associated with DED. The PHP-DPS@INS demonstrated remarkable cellular uptake, lysosomal escape, and mitochondrial targeting in vitro. Targeted metabolomics analysis revealed that PHP-DPS@INS effectively normalized the elevated level of mitochondrial proinflammatory metabolite fumarate in an in vitro hypertonic model of DED, thereby reducing the levels of key inflammatory factors (IL-1β, IL-6, and TNF-α). Additionally, PHP-DPS@INS strongly inhibited reactive oxygen species (ROS) production and facilitated mitochondrial structural repair. In vivo, the PHP-DPS@INS treatment significantly enhanced the adhesion duration and corneal permeability of the ocular surface in DED mice, thereby improving insulin bioavailability. It also restored tear secretion, suppressed ocular surface damage, and reduced inflammation in DED mice. Moreover, it demonstrated favorable safety profiles both in vitro and in vivo.

**Conclusion:**

In summary, this study successfully developed a comprehensive DED management nanosystem that overcame the ocular surface transmission barrier and disrupted the vicious cycle that lead to dry eye pathogenesis. Additionally, it pioneered the regulation of mitochondrial metabolites as an anti-inflammatory treatment for ocular conditions, presenting a safe, efficient, and innovative therapeutic strategy for DED and other inflammatory diseases.

**Supplementary Information:**

The online version contains supplementary material available at 10.1186/s12951-024-02503-7.

## Introduction

As one of the most prevalent ocular disorders, Dry Eye Disease (DED) greatly affects individuals’ Quality of Life (QoL) and social functioning, including activities such as reading, driving, and computer use. With a substantial prevalence worldwide, some of the DED symptoms include ocular pain, photophobia, lacrimation, and visual impairments [1]. The annual medical expenditure for DED in the United States is approximately $ 3.8 billion, imposing a huge economic burden on society [2]. The primary etiological factors contributing to DED include tear film instability, elevated osmotic pressure, and inflammation of the ocular surface [3]. The DED treatment approaches that target inflammation have garnered increasing attention in recent years, as inflammatory responses are considered a pivotal driver of the condition [4]. The therapeutic options for DED currently include Glucocorticoids (GCs) and Cyclosporine. However, prolonged GC use poses risks such as glaucoma, cataracts, and infections, while Cyclosporine can cause a burning sensation. These limitations impede their widespread use [5].

As “energy factories,” mitochondria play a crucial role in cellular metabolism [6]. Electron leakage from the mitochondrial respiratory chain could lead to the formation of Reactive Oxygen Species (ROS), which are believed to be the primary cause of Oxidative Stress (OS). Excessive ROS accumulation could result in mitochondrial dysfunction, including the aberrant opening of the Mitochondrial Permeability Transition Pore (MPTP) and loss of the mitochondrial membrane potential [7]. Recent studies revealed a strong association between mitochondrial metabolites and inflammatory reactions. Fumarate accumulation in the Tricarboxylic Acid (TCA) cycle within mitochondria triggers a robust inflammatory response, impairing mitochondrial function [8, 9]. The resulting mitochondrial dysfunction eventually aggravates the production of ROS and inflammatory factors, establishing a detrimental cycle in inflammatory diseases, encompassing OS, inflammatory responses, and mitochondrial dysfunction. A similar vicious cycle has been implicated in the pathogenesis of DED [4, 10, 11].

Insulin, a hormone commonly found in the body, has been shown to exert anti-inflammatory effects [12–14]. In this regard, topical insulin therapy has been effectively used to treat challenging ocular diseases such as persistent epithelial defect, chemical corneal epithelial injury, and refractory corneal ulcers [15–17]. According to previous research, the Insulin-like Growth Factor Binding Protein-3 (IGFBP-3) can restore mitochondrial function in hyperosmotic models of corneal epithelial cells, alleviating dry eye symptoms [18, 19]. Furthermore, Ger et al. designed Ce-sH nanoparticles for the treatment of alkaline corneal burns, with the goal of enhancing Statens Seruminstitut Rabbit Cornea (SIRC) cell proliferation through insulin-like growth factor 1 (IGF-1) [20]. Additionally, Titone et al. discovered that insulin could promote the accumulation of Insulin Receptors (INSR) in mitochondria and enhance mitochondrial homeostasis in corneal epithelial cells [21]. Based on these findings on insulin’s ability to improve mitochondrial dysfunction and reduce inflammatory responses within the eye, we speculated that it could be a potential treatment for DED. However, as a macromolecular peptide, insulin possesses inherent limitations, such as its large size, poor lipid solubility, and susceptibility to degradation. These limitations could result in a low intracellular delivery efficiency and bioavailability. Enhancing the intracellular delivery of protein and peptide drugs has long been a scientific challenge in research endeavors. Furthermore, ocular surface drug delivery remains an urgent issue that should be addressed. Traditional ophthalmic drugs have < 5% bioavailability due to tight corneal connections, rapid tear circulation, and tear film and blood-eye barriers [22]. Consequently, frequent administration of high drug concentrations is often necessary to achieve therapeutic effects in clinical practice, leading to an increased risk of drug side effects. The development of nanocarriers offers a promising strategy for enhancing the bioavailability of ophthalmic drugs [23]. Previous research has focused on the creation of innovative nanodrugs to overcome ocular barriers, aiming to improve drug bioavailability in the eye while minimizing side effects [24–27]. Therefore, there is a need to develop novel nanocarriers that can significantly enhance insulin bioavailability on the ocular surface.

Elamipretide (SS-31) is a peptide that specifically targets mitochondria [28]. Previous research showed that compared to its initial concentration, SS-31 concentration in the mitochondrial precipitation was significantly higher by approximately 5000-fold, indicating its exceptional ability to effectively target mitochondria [29]. Moreover, unlike conventional drugs targeting mitochondria, such as MitoQ, SS-31 uptake does not depend on the potential across the mitochondrial membrane and does not cause mitochondrial depolarization. Furthermore, SS-31 promotes mitochondrial cristae and respiratory chain integrity while reducing ROS production [30]. These mitochondrial targeting and antioxidant properties make SS-31 a promising candidate for the targeted alleviation of OS-related disorders and nanomaterial modification [31, 32].

**Scheme 1:**
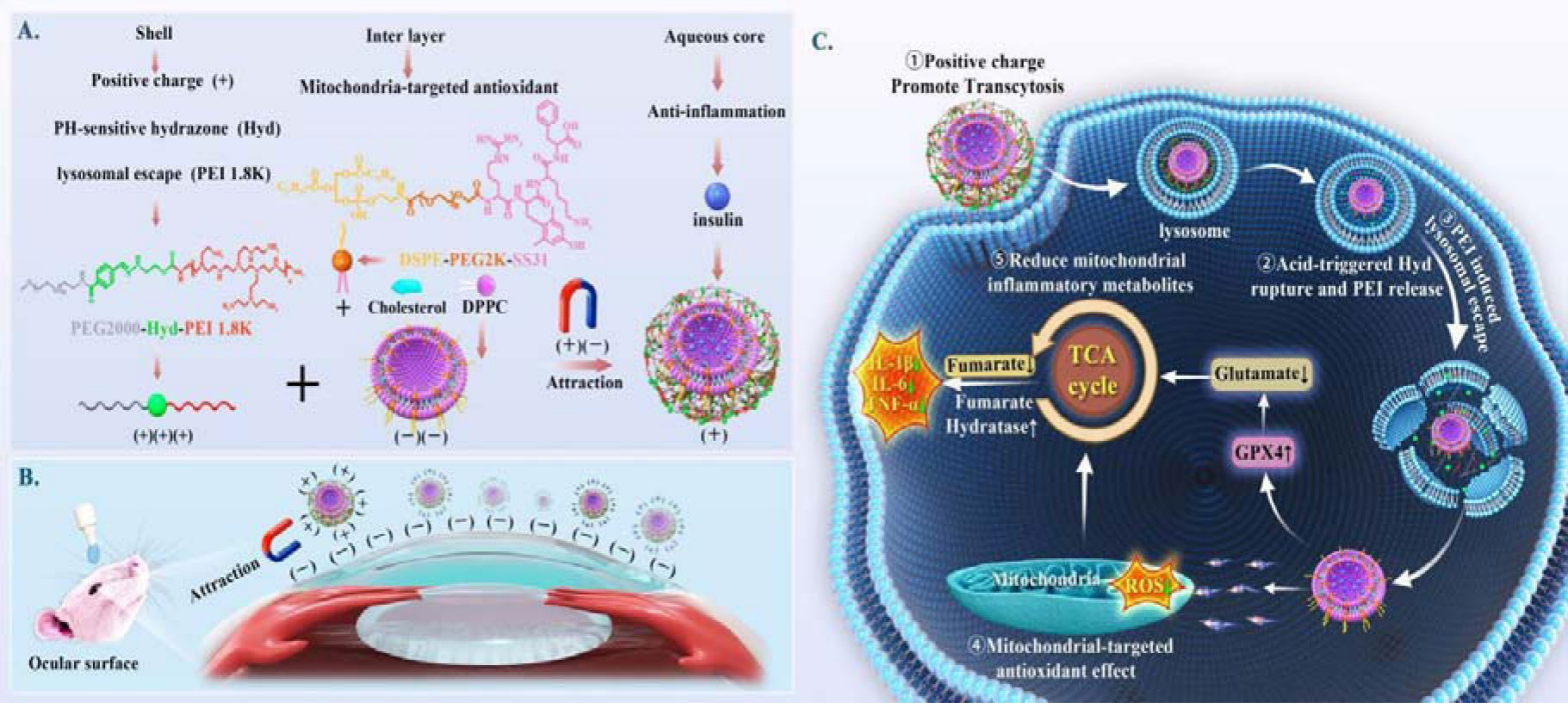
(**A**) PHP-DPS@INS NP synthesis scheme and layer functions. (**B**) Adhesion Function of PHP-DPS@INS NPs on the Ocular Surface. (**C**) The positive charge of PHP-DPS@INS NPs facilitates endocytosis, and the hyd bond undergoes an acid-mediated cleavage upon lysosomal entry, leading to PEI release and their subsequent rapid escape from the lysosome. Upon cytoplasmic entry, SS31 selectively targets mitochondria and exerts antioxidant effects and INS exerts anti-inflammatory actions by reducing fumarate levels in the TCA cycle. Together, these two components synergistically restore mitochondrial function and structure, disrupting the pathological vicious cycle of DED

In light of the favorable performance of nanomaterials in treating ocular diseases, some researchers have dedicated their efforts to developing nanomaterials that target the vicious circle mechanism in the pathogenesis of DED in recent years. Our team previously developed a novel tetrandrin-loaded liposome NPs(PFOB@LIP-Tet) for anti-inflammatory treatment of DED, which demonstrated significant efficacy in a rabbit model of DED [33]. Huang et al. also created mitochondria-targeted SkQ1 NPs for treating DED with a single antioxidant [34]. Recognizing that DED is a multifactorial disease, several studies have adopted a synergistic therapy strategy combining “anti-inflammatory-antioxidant” therapy and achieved superior efficacy compared to monotherapy [35–38]. Therefore, this study also adopts a synergistic therapeutic approach to address the vicious cycle of DED. We have developed a liposome nanoparticle with an “anti-inflammatory-antioxidant-mitochondrial repair” function (Scheme 1).The hierarchical liposomes were initially coated with a polymer shell (PEG2000-Hyd-PEI, PHP) to induce a surface charge reversal from negative to positive. This positively charged formulation promoted cellular endocytosis and adhesion to the negatively charged ocular surface [39]. The liposomes first entered lysosomes upon internalization via endocytosis. Subsequently, the acidic environment of lysosomes triggered the pH-sensitive hydride (hyd) bond within the liposome shell, leading to its rupture and release of PEIs (a positively charged polymers with proton sponge properties), facilitating their escape from lysosomes [40, 41]. The SS-31 peptide located in the middle layer of NPs further targeted mitochondria following their escape into the cytoplasm. Compared to conventional NPs, this delivery strategy involving the “ocular surface adhesion-lysosomal escape-mitochondrial targeting” pathway greatly improved cytoplasmic drug delivery and bioavailability. Moreover, co-administration of the antioxidant SS-31 peptide with anti-inflammatory insulin synergistically restored mitochondrial function and disrupted the vicious cycle associated with DED pathogenesis. Furthermore, high-throughput targeted metabolomics analysis revealed an abnormal build-up of proinflammatory metabolites (fumarate) in hypertonic Human Corneal Epithelial Cell (HCEC) models, which could be reduced by insulin or PHP-DPS@INS via GPX4 activation.

In an in vitro DED model, we observed that PHP-DPS@INS NPs exhibited efficient cellular uptake, escape from lysosomes, and specific mitochondrial targeting. These NPs also demonstrated significant capabilities in reducing ROS production and secretion of inflammatory factors (such as IL-1β, IL-6, and TNF-α), and a mitochondrial membrane repair potential. In vivo, the corneal epithelium showed significant healing, along with the inhibition of ocular surface inflammation and restoration of tear secretion, alleviating dry eye. Notably, the nanosystem designed herein represents the pioneering attempt to regulate TCA cycle metabolites for DED treatment. This study presents a multifunctional nanosystem capable of overcoming ocular surface barriers and integrating anti-inflammatory and anti-OS properties, as well as mitochondrial functionality for DED treatment, offering a novel, safe, and efficient therapeutic avenue.

## Results and discussion

### Preparation and characterization of the hierarchical nanosystem (PHP-DPS@INS NPs)

#### DSPE-PEG2k-SS31 synthesis

**Fig. 1:**
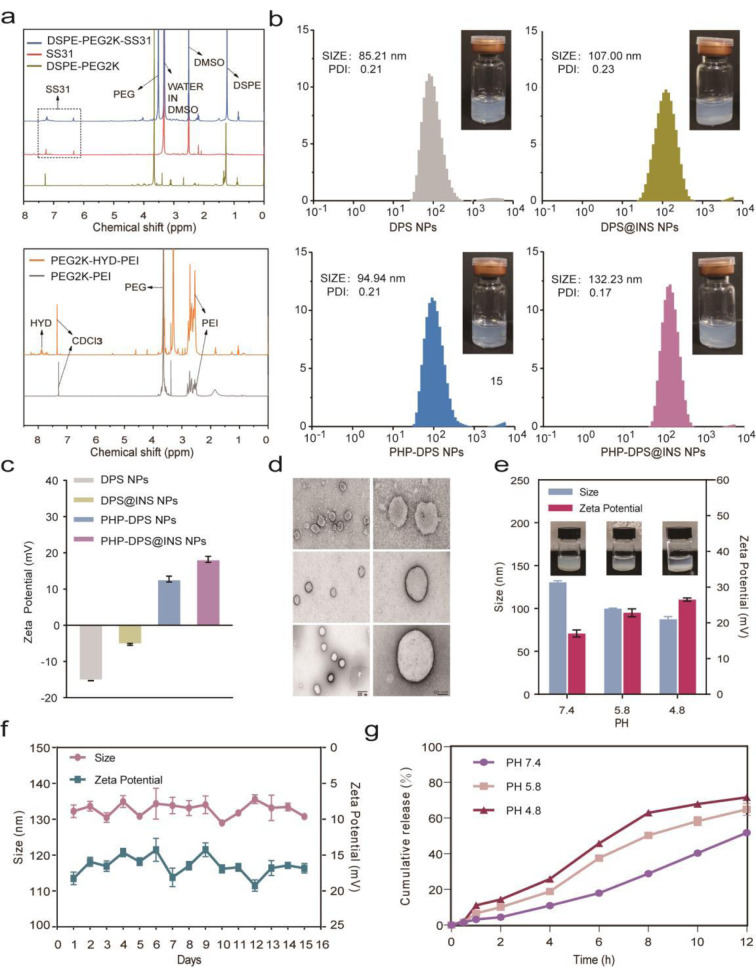
PHP-DPS@INS NP characterization. (**a**) ^1^H NMR spectra of DSPE-PEG2000-SS31 and PEG2000-Hyd-PEI. (**b**) Particle size, distribution, PDI, and appearance of DPS NPs, DPS@INS NPs, PHP-DPS NPs, and PHP-DPS@INS NPs. (**c**) Zeta potential of DPS NPs, DPS@INS NPs, PHP-DPS NPs, and PHP-DPS@INS NPs. (**d**) TEM image of DPS NPs, PHP-DPS NPs, and PHP-DPS@INS NPs (scale bar: 200 nm; 50 nm). (**e**) Variations in particle size, zeta potential, and appearance of PHP-DPS@INS NPs after 12 h at pH 7.4, 5.8, and 4.8. (**f**) DLS-measured changes in particle size and zeta potential of PHP-DPS@INS NPs over 15 days. (**g**) In vitro INS release from PHP-DPS@INS NPs under pH 7.4, 5.8, and 4.8 at 37 ℃ for 12 h

The amphiphilic polymer DSPE-PEG2k-SS31 (DPS) with mitochondria-targeted antioxidant activity was created by conjugating the SS31 peptide, a mitochondria-targeted antioxidant peptide, with a phospholipid bimolecular via PEG (Figure S1). Through ^1^H NMR analysis, DSPE-PEG2k-SS31 was confirmed to exhibit characteristic peaks at 7.4 and 6.3 ppm, as well as at 1.3 ppm (DSPE) and 3.6 ppm (PEG2k), consistent with those of the SS31 peptide and DSPE-PEG2k (DP), respectively (Fig. 1a).

#### PEG2000-Hyd-PEI synthesis

As a cationic polymer, PEI is commonly employed for lysosomal escape and has been extensively used as a carrier for nucleic acid substances [42], biomarkers, and vaccine adjuvants [43], as well as for NP modification. However, its strong positive charge could induce cytotoxicity [44]. This issue could be mitigated by introducing PEG modification to reduce PEI’s cytotoxicity and prevent macrophage clearance (Figure S2). The PEI-PEG connection is based on a hydride bond (Hyd), which is stable under neutral pH environments but cleaves under acidic conditions within the lysosome [45, 46], releasing PEI for the lysosomal escape of NPs (Figure S3). Characteristic peaks observed in the ^1^H NMR spectrum confirmed that PEG2000-Hyd-PEI displayed signals corresponding to PEI and PEG2000 at 2.5-3 ppm and 3.6 ppm, respectively. It also exhibited distinctive acyl hydride bond peaks at 7.5-8 ppm, which were absent in PEG2000-PEI samples (Fig. 1a).

#### PHP-DPS@INS NPs characterization

The DLS method was used to determine particle size, potential, dispersion, and stability. The DPS NPs and PHP-DPS NPs had average particle sizes of 85.21 ± 0.48 nm and 94.94 ± 1.79 nm, respectively. On the other hand, DPS@INS NPs had a particle size of 107.00 ± 2.95 nm, with PHP-DPS@INS NPs exhibiting the largest particle size at 132.23 ± 1.75 nm (Fig. 1b). These results demonstrate a gradual increase in NP size as a result of polymer coating and insulin loading, providing indirect evidence of successful liposome modification and INS encapsulation within NPs. Notably, all particles displayed a PDI below 0.3, with PHP-DPS@INS NPs having excellent particle regularity (PDI = 0.17 ± 0.01). The zeta potential of DPS NPs was determined to be -15.27 ± 0.06 mV. Due to the positive charge of INS, the zeta potential of DPS@INS NPs increased to -5.27 ± 0.26 mV upon INS loading. However, this absolute value is relatively small and renders the particles unstable. To address this issue, PEG2000-Hyd-PEI coating was applied, reversing the potential of PHP-DPS NPs and PHP-DPS@INS NPs, with PHP-DPS@INS NPs exhibiting a maximum potential of + 18.23 ± 0.84 mV (Fig. 1c). The increase in absolute NP potential enhances particle stability, while the charge reversal from negative to positive promotes adhesion on the ocular surface and cellular uptake. Through UHPLC analysis, we established the standard curve for insulin (Figure S4) and determined that the EE of insulin in PHP-DPS@INS NPs was 48.91 ± 0.36%, while the DL capacity was 9.78 ± 0.07% (Figure S5, Table S1). The TEM images reveal that DPS NPs exhibit the smallest diameter, and the liposome morphology is rendered unstable by the absence of PHP coating. Conversely, the diameter of PHP-DPS NPs increases further, resulting in a more stable liposome structure with a darker shell layer color, likely attributed to the presence of PHP coating. Meanwhile, PHP-DPS @INS NPs display the largest diameter (approximately 150 nm) among the three types, possibly due to INS loading. Furthermore, it is evident from the figure that PHP-DPS @INS NPs demonstrate uniform size and distribution (Fig. 1d).Furthermore, we also assessed the stability of PHP-DPS@INS NPs stored at RT over 15 days (Fig. 1f). Remarkably, during continuous monitoring, minimal fluctuations were observed in both particle size and zeta potential for DPS@INS NPs, indicating excellent stability at RT without any signs of leakage.

### The PH responsivity of PHP-DPS@INS NPs

Initially, we investigated the difference in pH between the normal and DED groups using both in vivo and in vitro models, which was critical to assess whether PHP-DPS@INS NPs is leaking outside the lysosome. Intracellular pH(pHi) was measured using BCECF-AM.Based on the CLSM images (Figure S6.a) and flow cytometry results (Figure S6.b, c), no significant difference in intracellular pH was observed between normal HCECs and hyperosmolar stress (HOS,450 mOsm/l) cultured HCECs. Additionally, the pH values of the ocular surface in normal mice and DED mice were assessed using pH paper, revealing a range of 7.0-7.5 with no statistically significant difference observed (Figure S7.a, b).Herein, we established pH gradients of 4.8, 5.8, and 7.4, given the pH discrepancy between lysosomes (4.5-5.0) and the intracellular environment (7.35–7.45). After 12 h, PHP-DPS@INS exhibited an increase in sediment, a decrease in particle size, and an increase in potential (Fig. 1e). This phenomenon could be attributed to the cleavage of hydride bonds (Hyd) in the NP shell (PEG2000-Hyd-PEI) under acidic conditions, decreasing the NP size and releasing PEI with a stronger positive charge. Furthermore, we conducted insulin release experiments in different pH environments and discovered that it increased with decreasing pH. We observed the fastest release rate at pH 4.8 (the lysosomal pH), indicating the pH responsiveness of PHP-DPS@INS NPs. We observed no significant burst release phenomenon in the three curves. After 12 h, the cumulative INS release from PHP-DPS@INS NPs was 51.75%±1.19, 64.91%±3.34, and 71.64%±1.18 at pH 7.4, 5.8 and 4.8, respectively (Fig. 1g, Table S2). Both DLS detection and drug release assays under various pH conditions confirmed the excellent pH responsiveness of PHP-DPS@INS NPs.

### Cytotoxicity and optimization of treatment conditions in vitro

According to the CCK8 assay results, the highest activity of HCECs was observed after 24 h of incubation in a normal medium at a concentration of 20 µg/ml INS (Figure S8.a) and 200 µg/ml PHP-DPS@INS NPs (Figure S8.b). Similarly, the optimal cell activity was achieved at a concentration of 200 µg/ml PHP-DPS@INS NPs in HOS (450 mOsm/l)-cultured HCECs, with a significantly higher cell activity observed at 12 h compared to 6 and 24 h (Figure S8.c). The live-dead staining results were consistent with those of the CCK-8 assay. As evidenced by fewer red-stained cells (indicative of dead cells) and increased numbers of green-stained live cells (Figure S8.d), HOS (450 mOsm/l)-cultured HCECs exhibited enhanced cell viability after 12 h of treatment with PHP-DPS@INS NPs. Consequently, we selected the 12-hour treatment with PHP-DPS@INS NPs (200 µg/ml) on HOS (450 mOsm/l)-cultured HCECs as the standard experimental condition for subsequent tests.

### Cellular uptake of PHP-DPS@INS NPs

**Fig. 2:**
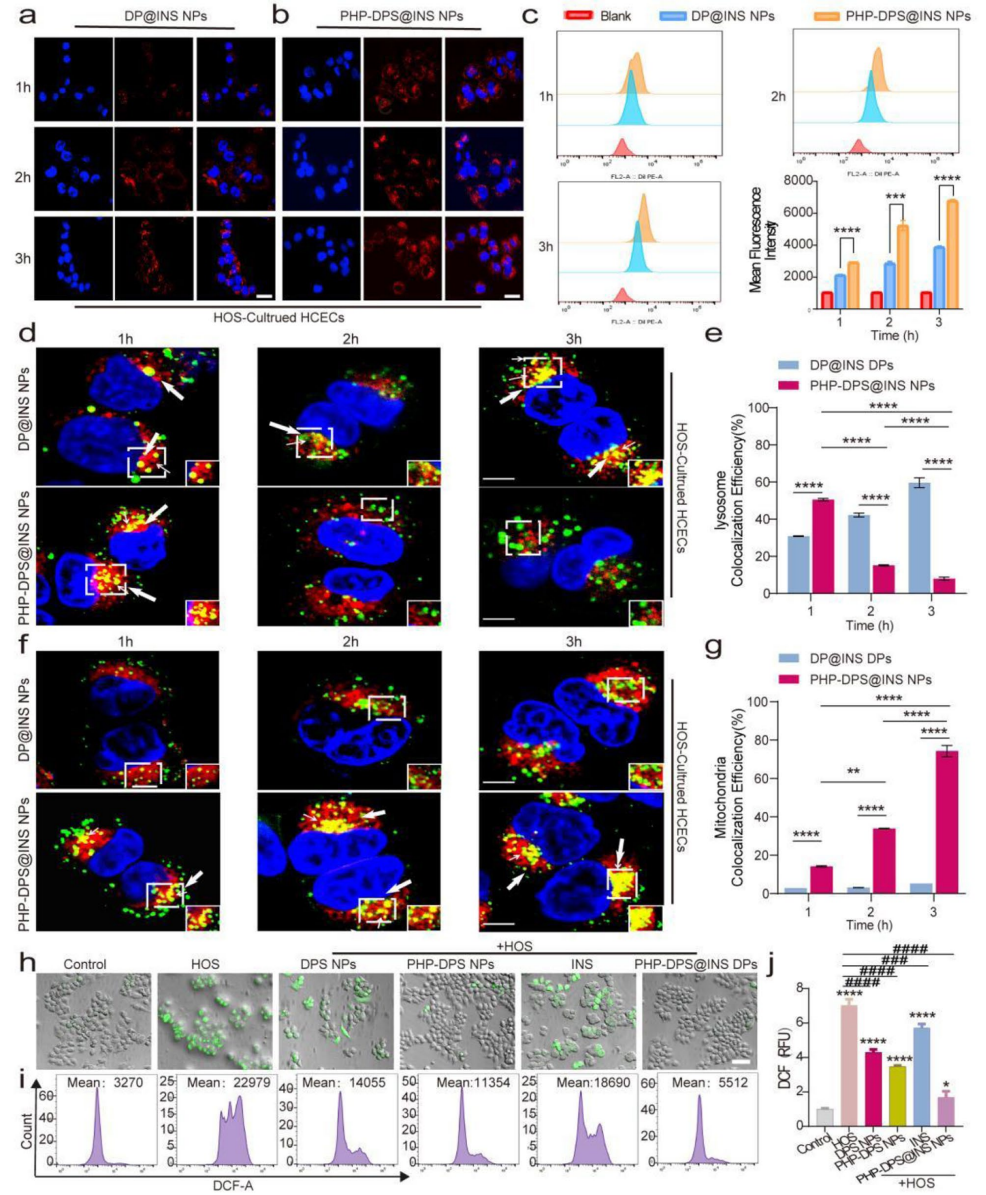
The effects of different nanoparticles on cell uptake, lysosomal escape, mitochondrial targeting and anti-ROS in HOS(450mOsM L^− 1^)-cultured HCECs. (**a**) Fluorescence images of HOS-cultured HCECs incubated with DP@INS NPs and (**b**) PHP-DPS@INS NPs at different treatment times (Blue: DAPI; Red: DiI-labeled nanoparticles; scale bars: 50 μm). (**c**) Flow cytometric curves and quantitative results of uptake of DiI-DP@INS NPs and DiI-PHP-DPS@INS NPs at 1, 2, and 3 h in HOS-cultured HCECs. (**d**) Lysosomal escape of DiO-DP@INS NPs and DiO-PHP-DPS@INS NPs at 1, 2, and 3 h in HOS-cultured HCECs by CLSM (Blue: DAPI; Green: DiO-labeled nanoparticles; Red: lysosomes labeled with Lysotracker Red; scale bars:10 μm). The white arrow represents the co-localization of nanoparticles and lysosomes. The figure in the lower right corner illustrates the representative image derived from the white square. (**e**) Quantitative results of fluorescence image about lysosome colocalization (*n* = 3 per group). (**f**) Mitochondrial targeting of DiO-DP@INS NPs and DiO-PHP-DPS@INS NPs at 1,2, and 3 h in HOS-cultured HCECs by CLSM (Blue: DAPI; Green: DiO-labeled nanoparticles; Red: mitochondria labeled with Mitotracker Red; scale bars:10 μm). The white arrow represents the co-localization of nanoparticles and mitochondria. The figure in the lower right corner displays the representative image selected by the white square. (**g**) Quantitative results of fluorescence image about Mitochondrial colocalization (*n* = 3 per group). (**h**) The bright-field image of ROS fluorescence in HOS-cultured HCECs after different treatments by DCFH-DA staining (scale bar:50 μm). (**i**) Flow cytometer diagram of DCFH-DA staining in different treatments. (**j**) Quantitative results of the average fluorescent intensity of ROS by flow cytometry. Data are presented as the mean ± SD, *P < 0.05, **P < 0.01, ***P < 0.001, and ****P < 0.0001 compared with control (normal) group; ^#^*p* < 0.05, ^##^*p* < 0.01, ^###^*p* < 0.001, and ^####^*p* < 0.0001 compared with HOS group

Flow cytometry and CLSM were used to compare the cellular uptake capabilities of DP@INS NPs and PHP-DPS@INS NPs, with both NPs labeled with DiI fluorescent dye (red). We first used normal HCECs to compare the uptake ability of DP@INS NPs and PHP-DPS@INS NPs. The fluorescence intensity of PHP-DPS@INS NPs was stronger than that of DP@INS NPs at different time points (Figure S9.a), which was further confirmed by flow cytometry (Figure S9.b, c). In addition, we also used HOS-cultured HCECs to compare the uptake ability of DP@INS NPs and PHP-DPS@INS NPs.According to the CLSM results, the fluorescence intensity for PHP-DPS@INS NPs was more significantly enhanced than that of DP@INS NPs within 3 h (Fig. 2a, b). On the other hand, the flow cytometry analysis results showed that the fluorescence intensity of PHP-DPS@INS NPs was almost twice as high as that of DP@INS NPs at both 2 and 3 h (*p* < 0.001) (Fig. 2c). These findings demonstrate that PHP-DPS@INS NPs exhibit enhanced cellular uptake compared to DP@INS NPs in both HCECs and a hypertonic model of HCECs.This implies a successful charge reversal of NPs following PEG2000-Hyd-PEI modification, where positively charged NPs (PHP-DPS@INS NPs) displayed enhanced favorability for cellular endocytosis relative to their negatively charged counterparts (DP@INS NPs).

### Lysosomal escape of PHP-DPS@INS NPs

Due to their biomembrane-like structure, liposomes offer significant advantages in enhancing cellular uptake and biocompatibility, making them widely utilized for drug delivery [47]. However, while liposomes can enhance cellular uptake, they do not necessarily improve drug bioavailability proportionally. This is because liposome nanoparticles enter cells through various endocytosis mechanisms and are transported to lysosomes, where a large number of drugs and their carriers are degraded. To enhance drug bioavailability, it is crucial for the nanoparticles to escape from lysosomes and be transferred to the cytoplasm - a process known as lysosomal escape [48]. The proton sponge effect is a commonly employed method for achieving lysosomal escape. Specifically, high-buffering drugs can activate proton pumps, leading to an increase in intralysosomal membrane potential. In order to achieve membrane equilibrium, chloride ions flow into lysosomes, further increasing intralysosomal osmotic pressure which results in lysosomal expansion and eventual rupture - facilitating drug release. Polyethyleneimine (PEI) serves as a typical representative of the proton sponge effect due to its high density of primary, secondary and tertiary amines [49, 50]. The PHP-DPS@INS NPs we designed feature an acid-responsive shell (PEG2000-Hyd-PEI), wherein the Hyd-bond can be cleaved in an acidic environment releasing PEI. PEI induces an increase in osmotic pressure within lysosomes due to its proton sponge effect causing influx of water into lysosomes ultimately resulting in swelling and rupture of the organelle. The inner component of the nanoparticles - DPS@INS NPs - then escapes from lysosomes transferring into the cytoplasm where it exerts therapeutic effects.

In order to test the lysosomal escape ability of PHP-DPS@INS NPs, we labeled both DP@INS NPs and PHP-DPS@INS NPs with the DIO fluorescent dye (green) and stained the nucleus (blue) and lysosomes (red) of HCECs. Through CLSM, we found that the yellow fluorescence of DP@INS NPs in HCECs, where NPs co-localized with lysosomes, was initially minimal at 1 h (30.84%) but gradually increased over time, becoming prominent in the cytoplasm at 3 h (59.62%). This observation suggests that DP@INS NPs are sequestered within lysosomes upon internalization and progressively accumulate over time. On the other hand, PHP-DPS@INS NPs exhibited higher levels of yellow fluorescence in the cytoplasm at 1 h (50.52%), which subsequently decreased at 2 and 3 h (15.15%, 7.98%), while the green fluorescence progressively increased. We observed that PHP-DPS@INS NPs entered the lysosome after 1 h, whereas a significant proportion of NPs successfully escaped the lysosomal compartment by the end of 2 h and localized within the cytoplasm of HCECs (Fig. 2d, e). These findings demonstrate the successful escape of PHP-DPS@INS NPs from lysosomes in HOS (450 mOsm/l)-cultured HCECs.

### Mitochondrial targeting of PHP-DPS@INS NPs

Several experiments have demonstrated the mitochondrial inner membrane targeting and antioxidant effects of the SS-31 peptide [28–32]. Furthermore, the SS-31 peptide has shown cell membrane penetration capabilities, ensuring efficient cytoplasmic delivery of insulin [29]. Consequently, we conjugated the SS31 peptide to a phospholipid bilayer via PEGylation and employed it as the interlayer of the liposome nanosystem. The co-localization of DP@INS NPs and PHP-DPS@INS NPs labeled with DIO (green fluorescence) and mitochondria (red fluorescence) was observed using CLSM. In the presented figure, the yellow fluorescence signal from DP@INS NPs, indicating their co-localization with mitochondria, consistently exhibited weak intensity across all periods (2.86%, 3.14%, 5.31%). This finding suggests a highly limited entry of DP@INS NPs into the mitochondria. Conversely, for PHP-DPS@INS NPs, the proportion of yellow fluorescence was only 14.22% at 1 h but gradually increased over time, reaching 74.27% at 3 h (Fig. 2f, g). These results demonstrate the progressive accumulation of PHP-DPS@INS NPs within the mitochondria, highlighting their efficient targeting capability.

The timing of mitochondrial targeting is crucial, corresponding to lysosomal escape. At 1 h, PHP-DPS@INS NPs undergo endocytosis, predominantly localizing within lysosomes, with only a minor fraction reaching the mitochondria. By 2 h, the majority of PHP-DPS@INS NPs have successfully evaded the lysosomes, initiating specific targeting towards the mitochondria. At 3 h, most PHP-DPS@INS NPs have already translocated from lysosomes to the mitochondria, while unmodified DP@INS NPs show no instances of lysosomal escape or mitochondrial targeting.

### The ROS scavenging ability of PHP-DPS@INS NPs in vitro

Oxidative stress plays a pivotal role in the pathogenesis of DED [51]. Intracellular ROS serve as crucial biomarkers for evaluating levels of oxidative stress, predominantly originating from mitochondria. Hence, precise targeting of mitochondria to eradicate ROS presents a promising therapeutic strategy [52]. The hyperosmolar stress (HOS) model, induced by exposing HCECs to a 450mOsM hyperosmolar medium, effectively mimics dry eye by promoting ROS production and inflammatory responses [53, 54]. Intracellular ROS levels (green fluorescence) were assessed using the DCFH-DA method. As shown in Fig. 2h, following hypertonic stimulation, a robust green fluorescence signal was observed in the HOS group, exhibiting significantly higher intensity compared to the Control group. In both DPS NPs and INS groups, there was a slight reduction in fluorescence intensity, while the PHP-DPS NPs and PHP-DPS@INS NPs treatment groups displayed a substantial attenuation of green fluorescence. Quantitative results obtained by flow cytometry further confirmed the aforementioned conclusions, indicating significant scavenging effects on ROS in all groups. The INS group demonstrated the lowest ROS reduction ability, only 18.66% lower than that of the HOS group. Moreover, DPS NPS, PHP-DPS NPs, and PHP-DPS@INS NPs displayed a remarkable decrease in ROS fluorescence compared to the HOS group with reductions of 38.83%, 50.59%, and 76.01%, respectively (Fig. 2i, j). Thus, PHP-DPS@INS NPs exhibit the most potent ability in reducing ROS levels. Nanoparticles loaded with the SS-31 peptide (DPS NPs, PHP-DPS NPs, and PHP-DPS@INS NPs) exhibit robust antioxidant capacity. Our findings reveal that PHP modification and INS co-incorporation synergistically amplify the antioxidant power of DPS NPs, culminating in unparalleled ROS removal capability. This translates to significantly enhanced potential for therapeutic applications where oxidative stress and inflammation are key players, such as in inflammatory diseases.

### The anti-inflammatory effects and potential therapeutic mechanisms in vitro

**Fig. 3:**
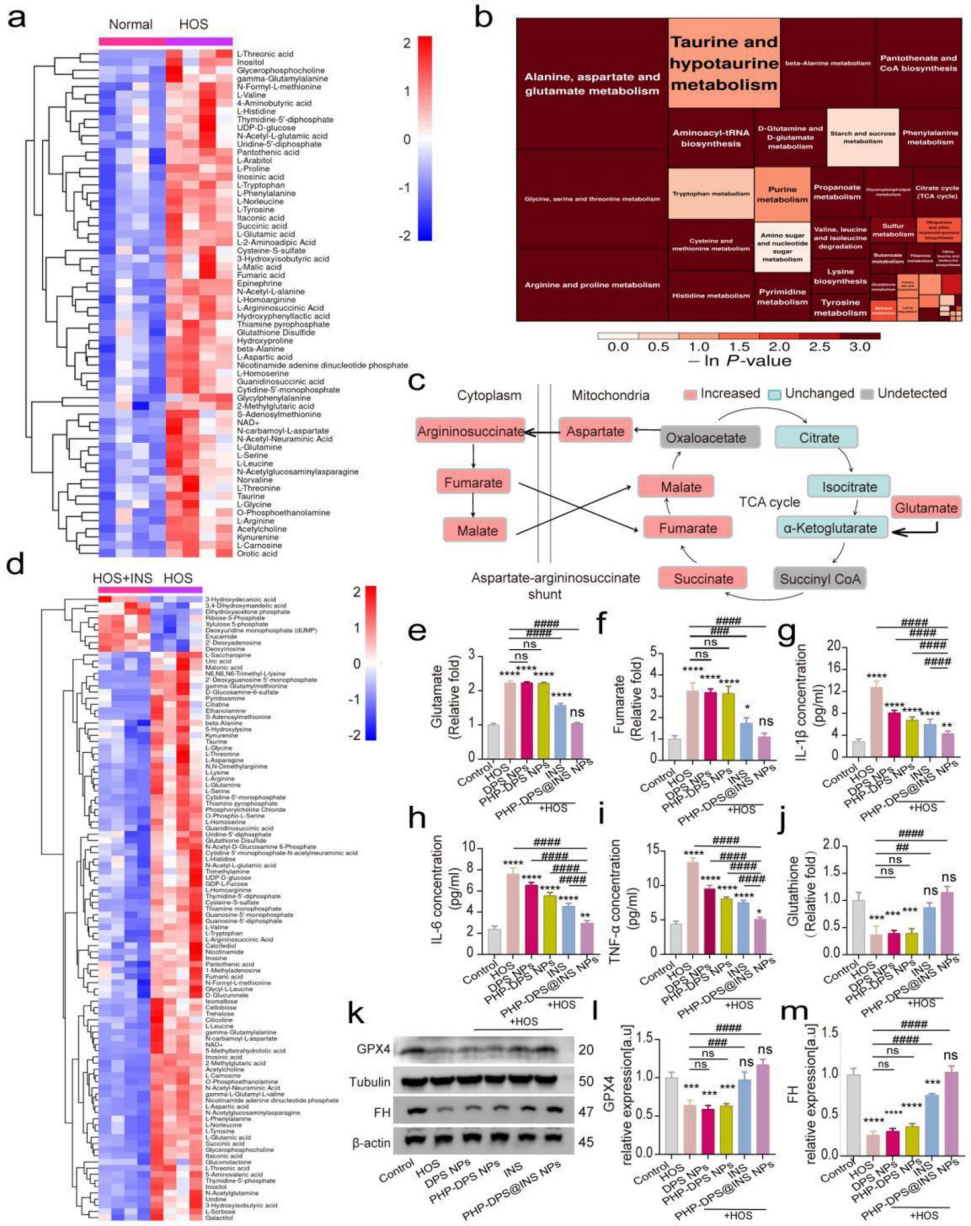
Targeted metabolomic analysis of the potential mechanism of PHP-DPS@INS NPs in the treatment of HOS(450mOsM L − 1)-cultured HCECs and the validation tests. (**a**) Hierarchical clustering analysis of metabolites between Normal group and HOS group(ordinate: the cluster of differential metabolites; red color: relatively high expression of the metabolites; blue color: relatively low expression of the metabolites). (**b**) Pathway analysis between Normal group and HOS group (square size: the influence factor size of this pathway in the topological analysis, the bigger the square, the greater the influence factor; square color: the degree of the enrichment analysis, the darker the color, the more significant the enrichment degree). (**c**) Metabolic changes in aspartate-argininosuccinate shunt and TCA cycle detected by targeted metabolomic analysis. (**d**) Hierarchical clustering analysis of metabolites between HOS + INS group(INS:20 µg/ml) and HOS group (ordinate: the cluster of differential metabolites; red color: relatively high expression of the metabolites; blue color: relatively low expression of the metabolites). (**e**) Glutamate and (**f**) Fumarate concentration of normal HCECs and HOS(450mOsM L^− 1^)-cultured HCECs following different treatments (*n* = 3). Using ELISA to detect (**g**) IL-1β, (**h**) IL-6 and (**i**) TNF-α concentration of normal HCECs and HOS (450mOsM L^− 1^)-cultured HCECs after different treatments. (**j**) Glutathione concentration of normal HCECs and HOS(450mOsM L^− 1^)-cultured HCECs after different treatments (*n* = 3). (**k**) Representative Western Blotting images of GPX4 AND FH in normal HCECs and HOS(450mOsM L^− 1^)-cultured HCECs after different treatments. Quantitative analysis of WB bands about relative expression of GPX4 (**l**) and FH (**m**). Data are presented as the mean ± SD, *P < 0.05, **P < 0.01, ***P < 0.001, and ****P < 0.0001 compared with control(normal) group; ^#^*P* < 0.05, ^##^*P* < 0.01, ^###^*P* < 0.001, and ^####^*P* < 0.0001 compared with HOS group. Normal/Control group: normal cultured HCECs; HOS: HCECs cultured in 450mOsM for 12 h; HOS + INS: HCECs cultured in 450mOsM for 12 h and then added with 20 µg/ml INS for another 12 h; VS: versus; ln *P*-value: the negative logarithm of the P value; GPX4: glutathione peroxidase; FH: fumarate hydratase

The early identification of insulin-like growth factor 1 receptor (IGF-1R) and INSR in the cornea underscores the essential association between insulin and corneal tissue [55, 56]. Importantly, unlike most bodily tissues, insulin is not required for glucose uptake in the corneal epithelium [57, 58]. Therefore, our primary focus is on exploring other impacts of insulin on corneal tissue. In recent years, increasing attention has been directed towards the anti-inflammatory effect of insulin [12–14] and its promising therapeutic potential for corneal inflammatory diseases [15–17], although further investigation is necessary to elucidate its underlying mechanism. Given the close association between insulin and mitochondrial dysfunction in metabolism, we initially employed high-throughput targeted metabolomics to assess alterations in metabolites within HCECs stimulated by HOS (450mOsm/L for 12 h, HOS group) and conventionally cultured HCECs (Normal group). The thermogram clearly illustrates significant disparities in metabolites between the normal group and the HOS group (Fig. 3a, Table S3). Through a comprehensive analysis of pathways associated with differential metabolites, including enrichment and topology analyses, our findings revealed a significant enrichment of metabolic pathways involving alanine, aspartate, and glutamate (Fig. 3b, Table S4, Figure S10). We further treated the HOS group with 20 µg/ml INS (12 h) for therapeutic purposes. As shown in the thermogram, the previously elevated metabolites in the HOS group exhibited a significant reduction upon the addition of INS (Fig. 3d, Table S5). The OPLS-DA score plot demonstrated a substantial distinction between the two sample groups, all falling within the 95% confidence interval (Figure S11,12). The KEGG pathway diagram of alanine, aspartate, and glutamate metabolism illustrates that α-ketoglutarate serves as the entry point for glutamate into the TCA cycle, providing essential substrates [59]. Consequently, an increase in glutamate levels promotes the subsequent elevation of TCA metabolites such as succinate, fumarate, and malate. Notably, fumarate can undergo recycling within the TCA cycle through the aspartic-arginine succinate shunt, leading to a further escalation in its content (Fig. 3c, Figure S13). Recent literature has highlighted that an aberrant increase in mitochondrial fumarate levels can elicit a robust inflammatory response, accompanied by the inhibition of fumarate hydratase (FH) [8, 9], thus establishing a detrimental feedback loop to further enhance fumarate accumulation. The metabonomics results suggest that INS significantly inhibits the elevation of glutamate and metabolites in the TCA cycle in the HOS model of HCECs. Therefore, we ∼ hypothesized that INS may attenuate glutamate levels, subsequently reducing TCA metabolite levels, particularly the inflammatory metabolite - fumarate, and alleviating FH enzyme inhibition, ultimately resulting in anti-inflammatory effects. To validate the hypothesis, we employed glutamate, fumarate, and inflammatory factor kits for detection (Fig. 3e-i). The findings demonstrated a significant elevation in glutamate, and fumarate, as well as inflammatory factors IL-1β, IL-6, and TNF-α levels within the HOS group compared to the normal group (*P* < 0.0001). After the treatments, no significant changes were observed in glutamate and fumarate levels between the DPS NPs and PHP-DPS NPs treatment groups when compared to the HOS group; however, a slight reduction was observed in the inflammatory factors IL-1β, IL-6, and TNF-α, attributable to the antioxidant effect of the SS-31 peptide. Moreover, both glutamate and fumarate levels were significantly lower in the INS group and PHP-DPS@INS NPs treatment group compared to those in the HOS group. WB results indicated a significant decrease in FH expression in the HOS group compared to the normal group (Fig. 3k, m). Treatment with DPS NPs and PHP-DPS NPs did not lead to significant changes in FH expression. However, treatment with INS and PHP-DPS@INS NPs resulted in a significant increase in FH expression, with the most pronounced increase observed in the PHP-DPS@INS NPs group. These findings align with the metabolomics results, supporting our hypothesis that INS’s anti-inflammatory effect on the HCEC hypertonic model is associated with reduced levels of glutamate and fumarate, as well as activation of the FH enzyme.

INS can induce a reduction in glutamate levels through the activation of glutathione peroxidase 4 (GPX4) protein, leading to reduced fumarate levels and subsequent activation of the FH enzyme. The intracellular glutamate content is closely associated with the GPX4 protein. The cystine/glutamate reverse transport system Xc- on the cell membrane facilitates the efflux of intracellular glutamate into the extracellular environment, resulting in a decrease in intracellular glutamate levels [60]. Simultaneously, cystine is imported into the cell for glutathione synthesis. Glutathione serves as a substrate for GPX4, and an increase in glutathione levels can enhance GPX4 activity. Consequently, the activation of GPX4 contributes to decreased intracellular glutamate concentrations. Wu et al. demonstrated that insulin-like growth factor 1 (IGF-1) activation in human liver cancer cells upregulates the expression of GPX4 [61]. Our previous research demonstrated that insulin increased GPX4 expression and attenuated glutamate levels in HCECs under H_2_O_2_-induced inflammatory conditions [62].In an HOS model of dry eye, insulin may also mitigate intracellular glutamate levels by activating the GPX4 protein. We evaluated intracellular GPX4 levels (Fig. 3k, l) and intracellular glutathione content (Fig. 3j). The results demonstrated a significant inhibition of GPX4 protein expression and a notable decrease in intracellular glutathione content within the HOS model group. Treatment with INS or PHP-DPS@INS NPs significantly upregulated GPX4 and increased glutathione levels compared to control, unlike DPS and PHP-DPS NPs alone. This suggests that INS plays a key role in activating antioxidant pathways, further enhanced by the presence of PHP in the combined group.

In summary, INS can reduce intracellular glutamate levels by enhancing GPX4 activity in HOS(450mOsM/l)-cultured HCECs. This reduction in glutamate concentration leads to a depletion of tricarboxylic acid cycle substrates, resulting in decreased inflammatory metabolite fumarate. Our findings reveal a promising anti-inflammatory strategy: targeting the vicious cycle of fumarate accumulation. Decreasing fumarate levels via this approach triggers a cascade of positive effects, including reduced inflammatory mediators like IL-1β, IL-6, and TNF-α. Furthermore, utilizing nanoparticles (PHP-DPS@INS NPs) to deliver INS significantly increases its effectiveness, opening doors for potential therapeutic applications.

### The reparative efficacy of PHP-DPS@INS NPs for mitochondrial dysfunction in vitro

#### PHP-DPS@INS NPs restore mitochondrial membrane polarization

**Fig. 4:**
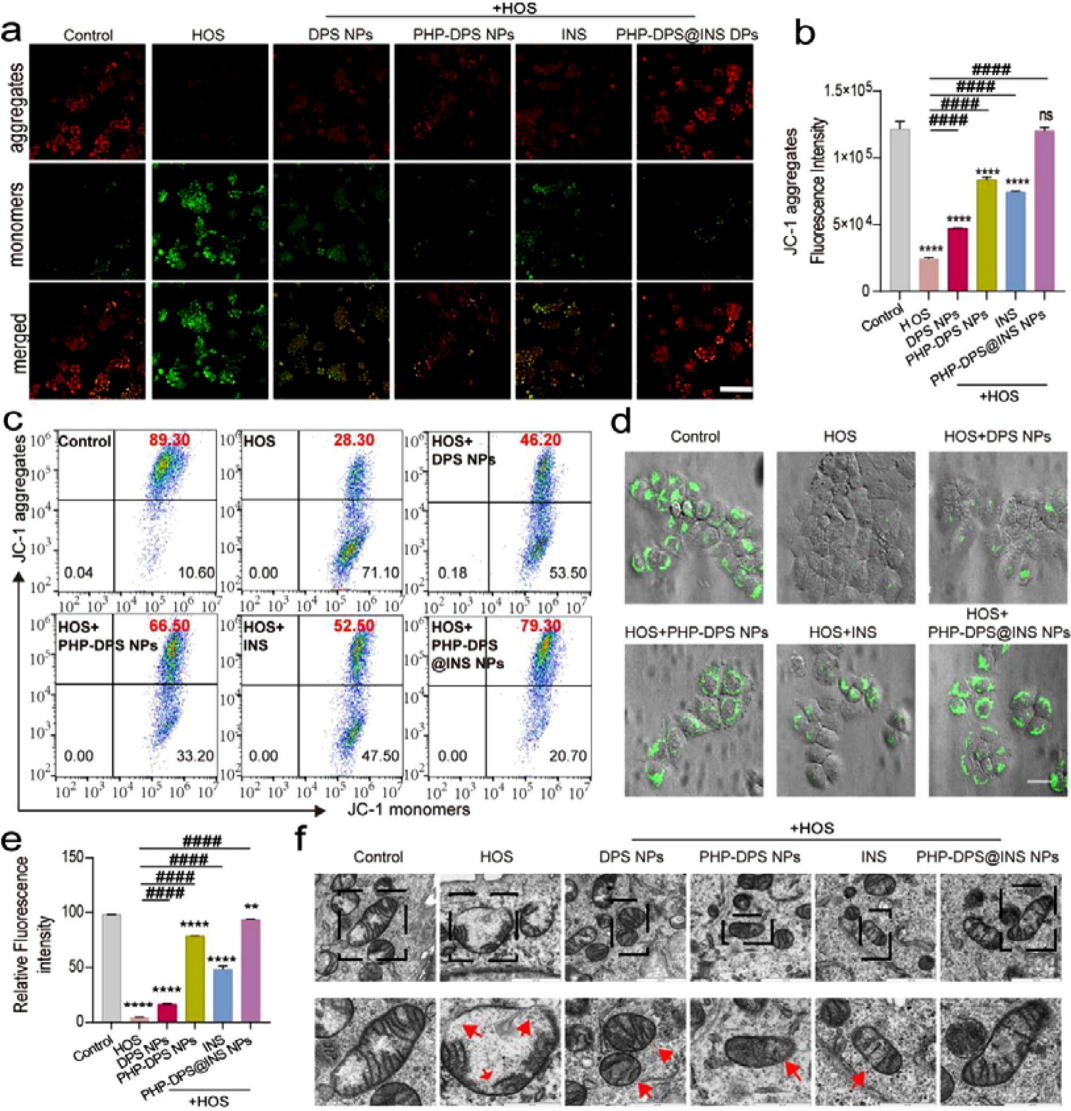
PHP-DPS@INS NPs alleviate mitochondrial dysfunction of HOS(450mOsM L^− 1^)-cultured HCECs. (**a**) Fluorescence images of mitochondria membrane potential changes of normal HECEs and HOS(450mOsM L^− 1^)-cultured HCECs after different treatments (scale bar: 50 μm). (**b**) Quantitative analysis of JC-1 aggregates examined by flow cytometer. (**c**) JC-1 fluorescence detected by flow cytometer in normal HECEs and HOS (450mOsM L^− 1^)-cultured HCECs after different treatments. (**d**) Fluorescence images (scale bar:25 μm) and (**e**) quantitative analysis of restoration of mPTPs in the normal HECEs and HOS(450mOsM L^− 1^)-cultured HCECs after different treatments by Calcein AM/CoCl_2_ staining. (**f**) TEM images of the mitochondrial ultrastructure of normal HCECs, HOS(450mOsM L^− 1^)-cultured HCECs, and HOS(450mOsM L^− 1^)-cultured HCECs treated with different treatments (scale: 1000 nm).The next row (scale bar:600 nm) is an enlarged illustration of the black boxes of the respective images in the previous row (Red arrow: damaged mitochondria). Data are presented as mean ± SD, *P < 0.05, **P < 0.01, ***P < 0.001, and ****P < 0.0001 compared with control (normal) group; ^#^*p* < 0.05, ^##^*p* < 0.01, ^###^*p* < 0.001, and ^####^*p* < 0.0001 compared with HOS group. Normal/Control: normal cultured HCECs; HOS: HCECs cultured in 450mOsM for 12 h; mPTPs: mitochondrial permeability transition pores

The preservation of normal mitochondrial function relies on maintaining a well-preserved mitochondrial membrane potential, with deviations from this potential closely associated with mitochondrial diseases [63]. Thus, assessing membrane potential serves as a crucial parameter for evaluating mitochondrial function. The evaluation of mitochondrial membrane potential was conducted using JC-1, which exists as a polymer in normally polarized mitochondria, exhibiting red fluorescence. In contrast, depolarized mitochondria display green fluorescence due to the presence of JC-1 monomers. As illustrated in the figure, the HOS group showed a significant depolarization of mitochondrial membrane potential, indicated by intense green fluorescence. The use of DPS NPs, PHP-DPS NPs, and INS led to an improvement in membrane potential, although the red fluorescence remained faint. Remarkably, PHP-DPS@INS NPs demonstrated a significant improvement in membrane potential depolarization, evident through red fluorescence (Fig. 4a). The quantitative results obtained through flow cytometry were consistent with those from CLSM. As the JC-1 polymer serves as an indicator of normal mitochondrial function, we conducted a quantitative analysis to assess the JC-1 polymer channel’s proportion. The JC-1 polymer percentage in the HOS group (28.30%) was markedly lower compared to the control group (89.3%). Conversely, there was a slight increase in JC-1 polymer percentages in the DPS NPs treatment group (46.20%), INS treatment group (52.5%), PHP-DPS NPs treatment group (66.50%), and a notably higher increase in the PHP-DPS@INS NPs treatment group (79.30%), as shown in Fig. 4b, c. These findings suggest that while DPS NPs, INS, and PHP-DPS NPs treatments exhibit some reparative effects on mitochondrial membrane potential, their efficacy is limited. Notably, PHP-DPS@INS NPs treatment demonstrates a pronounced restorative effect on mitochondrial membrane potential.

#### PHP-DPS@INS NPs contribute to the repair of the mitochondrial permeability transition pore (mPTP)

mPTP is a histone complex located between the inner and outer mitochondrial membranes. Excessive opening of mPTP can result in depolarization, swelling, and dysfunction of mitochondria. The openness of mPTP was assessed using Calcein AM/CoCl_2_. Upon mPTP closure, only green fluorescence localized within the mitochondria was observed; conversely, upon mPTP opening, all cytoplasmic green fluorescence disappeared. The CLSM figure illustrates that the green fluorescence in the HOS group was nearly completely quenched, showing a 95.25% reduction in fluorescence intensity compared to the normal group. This observation indicates that HOS induces abnormal mPTP opening in HCEC. However, treatment with DPS NPs, INS, and PHP-DPS NPs led to a partial recovery of green fluorescence, while the PHP-DPS@INS NPs treatment group exhibited substantial recovery. Notably, the PHP-DPS@INS NPs group demonstrated only a marginal decrease of 4.72% compared to the normal group, signifying a substantial restoration of mPTP (Fig. 4d, e).

#### Effects of PHP-DPS@INS NPs on mitochondrial structure

The effect of PHP-DPS@INS NPs on the mitochondrial structure of HCEC was observed using TEM. As illustrated in Fig. 4s (red arrows indicating aberrant mitochondrial regions), mitochondria within the HOS model exhibited swelling, spherical morphology, partial vacuolation, and a contraction of internal cristae structure towards the periphery or even rupture of the mitochondrial membrane. The DPS NPs treatment group displayed reduced mitochondrial swelling, decreased internal vacuolation, and partial restoration of internal cristae structure. Furthermore, both PHP-DPS NPs and INS treatment groups demonstrated a further reduction in mitochondrial swelling; however, disordered cristae structure persisted, with only the PHP-DPS@INS NPs group displaying elongated and healthy morphology.

In summary, the PHP-DPS@INS NPs treatment group demonstrates a more pronounced efficacy in inhibiting excessive opening of the mPTP, reducing mitochondrial membrane depolarization, and ultimately restoring mitochondrial function compared to other treatment groups. These findings reveal a remarkable synergy: combining anti-inflammatory and antioxidant treatments significantly outperforms individual applications in repairing mitochondrial function. This cumulative effect offers a promising therapeutic avenue for breaking the destructive loop of “inflammation-oxidative stress-mitochondrial dysfunction” in inflammatory diseases.

### PHP-DPS@INS NPs enhance ocular surface retention and corneal penetration in vivo

#### Optical coherence tomography (OCT) assessment of the ocular surface distribution

**Fig. 5:**
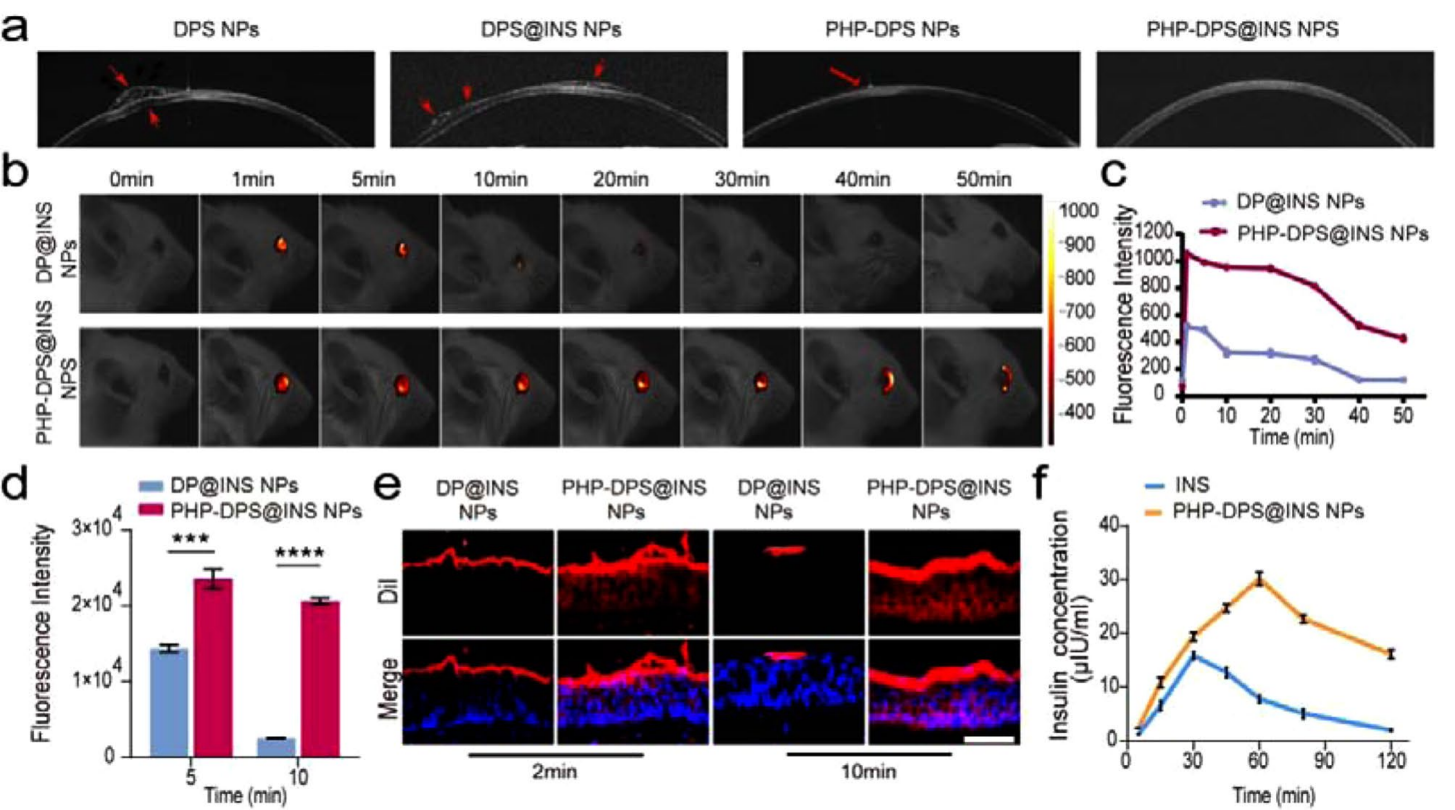
Retention, uptake and corneal permeabilitys of different nanoparticles on ocular surface. (**a**) Optical coherence tomography images of different nanoparticles on ocular surface of New Zealand white rabbits (red arrow: uneven distribution of nanoparticles on cornea and corneal compression). (**b**) Fluorescence images and (**c**) Quantitative analysis of DP@INS NPs and PHP-DPS@INS NPs on eyes of BAK-induced DED model mice at different time points. Nanoparticles are labeled by DiI. (**e**) Fluorescent images (scale bar: 50 μm) and (**d**) quantitative analysis of penetration and retention of DP@INS NPs and PHP-DPS@INS NPs in ocular surface of BAK-induced DED mice by frozen sections at 2 and 10 min. Nanoparticles are labeled by DiI. (**f**) Curve of insulin concentration of INS and PHP-DPS@INS NPs entering the anterior chamber of BAK-induced DED mice at different time points

OCT, a noninvasive and noncontact imaging technique with high resolution [64], facilitates the diagnosis of various ocular diseases. Due to the small size of babl/c mouse corneas, normal New Zealand white rabbits were chosen for OCT detection to visualize ocular surface adhesion in each treatment group. After 3 min of administration, OCT images revealed significant agglomeration (indicated by red arrows) and slight corneal deformation due to compression in the DPS NPs suspension. While DPS@INS nanoparticles clumped on the cornea (red arrows), PHP-DPS and PHP-DPS@INS suspensions showed improved distribution. Notably, only PHP-DPS@INS displayed a uniform spread across the cornea (Fig. 5a). This suggests PHP-DPS@INS are better at achieving a homogeneous distribution on the eye surface, likely due to their increased charge that promotes nanoparticle dispersion. In vivo, fluorescence for ocular surface retention time of PHP-DPS@INS NPs: DiI-labeled DP@INS NPs and PHP-DPS@INS NPs were applied to the eyes of DED mice and observed using a small animal fluorescence imaging system (Viber lourmat, France). The fluorescence of both nanoparticles exhibited distinct degrees of attenuation over time following administration. After 5–10 min, the fluorescence intensity of DP@INS NPs significantly decreased by 37.06%, further attenuation by 47.22% at the 30-minute mark. PHP-DPS@INS NPs exhibited a markedly slower attenuation rate compared to DP@INS NPs (*p* < 0.05). Notably, at the 30-minute time point, PHP-DPS@INS NPs demonstrated only a 13.27% decrease in activity, highlighting their enhanced stability and sustained therapeutic potential.At the 40-minute time point, the fluorescence of PHP-DPS@INS NPs on the cornea of mice began to weaken significantly, and by the 50th minute, the fluorescence had mainly accumulated in the inner canthus (nasolacrimal duct drainage). At the same time, at the 40th minute, the fluorescence of DP@INS NPs on the cornea of mice was almost undetectable (Fig. 5b, c). The ocular surface retention duration of PHP-DPS@INS NPs was significantly prolonged compared to that of DP@INS NPs, potentially attributed to the positive charge attribute of PHP-DPS@INS NPs enhancing adhesion with the negatively charged ocular surface. In contrast, the restricted adherence of DP@INS NPs to the ocular surface led to their rapid removal through blinking and tear circulation.

#### Frozen sections to observe corneal epithelial permeability

DiI-labeled DP@INS NPs and PHP-DPS@INS NPs were applied to the eyes of DED mice, and the corneal epithelial permeability of the nanoparticles was observed using corneal frozen sections. At 2 min, DP@INS NPs were predominantly localized on the corneal surface, whereas PHP-DPS@INS NPs had successfully penetrated the superficial corneal epithelium. By 10 min, DP@INS NPs remained confined to the corneal surface with a significant washout effect (fluorescence decreased by 82.09%). Compared to other formulations, PHP-DPS@INS NPs exhibited superior adhesion and deeper penetration into the corneal epithelium (Fig. 5d, e). This suggests their unique properties, likely linked to the presence of PHP and INS, facilitate efficient drug permeation through the ocular barrier, ultimately enhancing drug availability within the eye.

#### Pharmacokinetics of insulin in aqueous humor

To investigate the penetration of nanoparticle-loaded drugs into the anterior segment, eye drops containing INS(35 µg/ml) and PHP-DPS@INS NPs(with INS:35 µg/ml) were administered to DED mice, and the concentration of INS in aqueous humor was quantified using an ELISA kit at different time points. The concentration of INS in aqueous humor peaked at 30 min (15.89 ± 0.71 µIU/ml) and returned to near baseline levels by 120 min (2.06 ± 0.14 µIU/ml). The concentration of PHP-DPS@INS NPs in aqueous humor was significantly higher than that of pure INS at all time points (*P* < 0.05). At 60 min, INS reached its peak level in the aqueous humor (30.11 ± 1.24 µIU/ml), and by the end of the experiment, it maintained a consistent concentration in the aqueous humor (16.11 ± 0.84 µIU/ml) (Fig. 5f). These results demonstrate that PHP-DPS@INS NPs not only enhance the concentration of INS entering the anterior segment but also prolong the release duration of INS compared to INS alone.

The above experiments have illustrated that PHP-DPS@INS NPs exhibit excellent capability in penetrating the ocular surface barrier. In comparison with other treatment groups, PHP-DPS@INS NPs display a more uniform distribution on the corneal surface, along with enhanced ocular surface adhesion time and corneal penetration capacity. These findings significantly contributed to the improvement of drug bioavailability in the cornea.

### Clinical evaluation of PHP-DPS@INS NPs in DED mice

In clinical practice, fluorescein staining, tear Break Up Time (tBUT), and tear secretion experiments are commonly employed as diagnostic tests and tools for evaluating the efficacy of DED treatment [65]. Therefore, we utilized the aforementioned tests to assess the effectiveness of PHP-DPS@INS NPs in DED mice.

#### Repair effect of PHP-DPS@INS NPs on corneal epithelium and conjunctival goblet cell

**Fig. 6:**
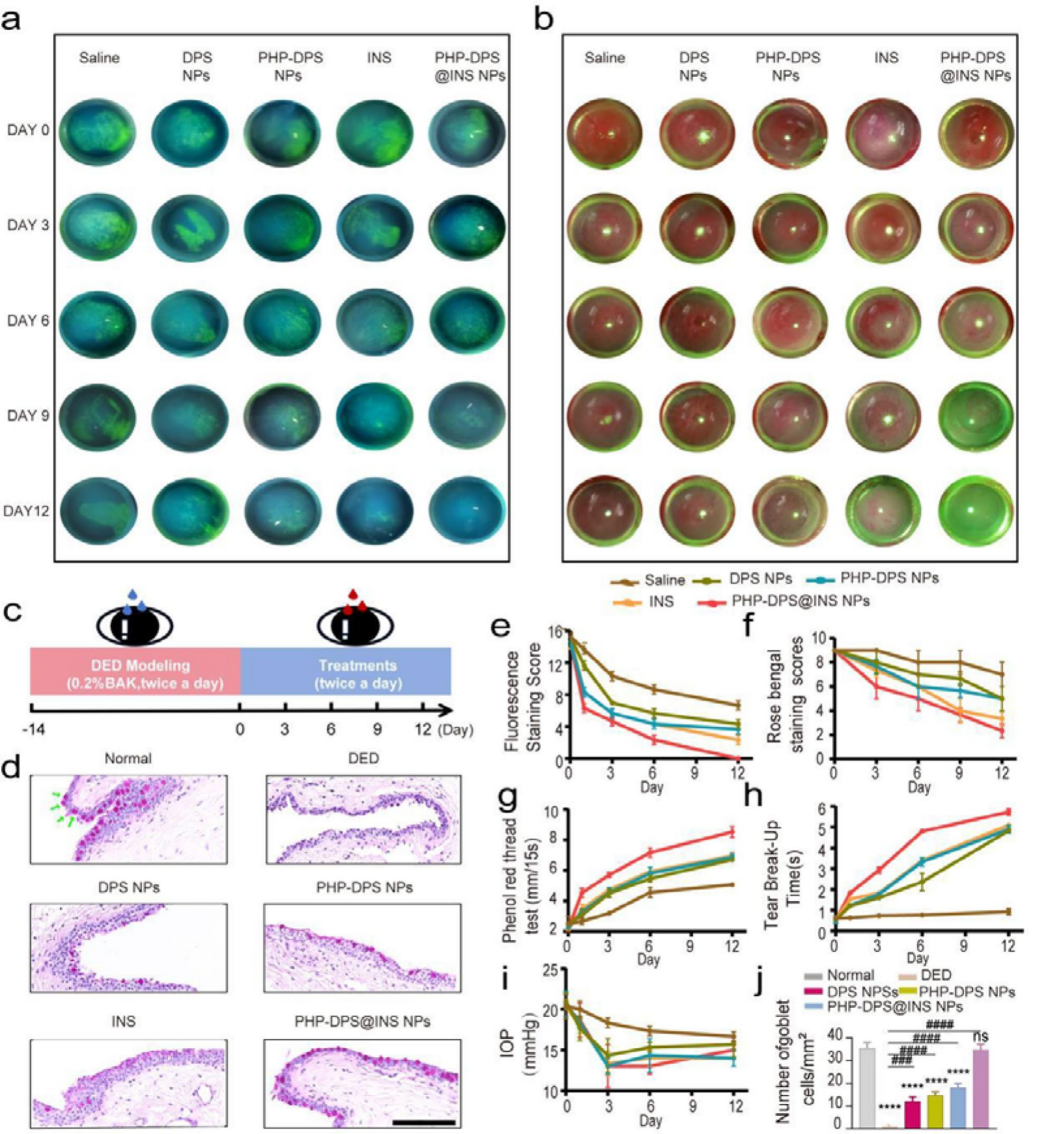
Therapeutic effects of different treatments on ocular surface.(**a**) Images of sodium fluorescein staining, (**e**) Sodium fluorescein staining score, (**b**) rose bengal staining images, (**f**) rose bengal staining scores, (**g**) Quantification of the tear production (length of wet part of phenol red thread), (**h**) Tear break-up time, and (**l**) Intraocular pressure (IOP) of BAK-induced DED mice received different treatments on 0,3, 6, 9, and 12 days, respectively.(**c**) Establishment of in vivo DED model and diagram of the treatment plan. (**d**) PAS staining and (**j**) quantitative analysis of conjunctival goblet cells (scale bar:50 μm) of the BAK-induced DED mice after different treatments.Data are presented as mean ± SD; *n* = 3. BAK: benzalkonium chloride; BAK-induced DED mice: the eye of balb/c mice received 0.2% benzalkonium chloride, 5ul at a time, twice daily (8:00am and 8:00 Pm) for 14 days; Day0: The model of BAK-induced DED mice has just been completed and has not received any treatment

DED induces varying degrees of corneal epithelial damage, leading to the appearance of green fluorescence staining on the corneal epithelial defects. To quantify the efficacy of ocular surface repair, we utilized a 16-point scoring system for fluorescence staining [66].On the third day, all groups, including Saline, DPS NPs, PHP-DPS NPs, and INS, exhibited epithelial staining scores greater than 5 (scores were 10.33 ± 0.58, 7.33 ± 0.58, 5.67 ± 0.58 and 5.33 ± 0.58, respectively), suggesting the persistence of relatively severe epithelial injury at this time point. However, on the third day, PHP-DPS@INS NPs exhibited the lowest score (score: 4.33 ± 0.58) among all groups, indicating its superior efficacy in promoting epithelial repair compared to other treatments. Nevertheless, complete healing of the epithelium had not yet been achieved. By the 12th day, the staining score of the Saline group remained > 6, and significant epithelial damage persisted. The DPS NPs group, PHP-DPS NPs group, and INS group had scores of 4.33 ± 0.58, 3.67 ± 0.58, and 2.33 ± 0.58, respectively, with a small amount of fluorescence still observed in the corneal epithelium. These findings indicate that the treatments mentioned had a discernable effect on DED treatment, although with limited efficacy. Notably, only the PHP-DPS@INS NPs group displayed minimal fluorescence staining in the corneal epithelium after 12 days of treatment (Fig. 6a, e). Rose Bengal staining is used to detect abnormal corneal epithelial and conjunctival epithelial cells in DED.The results revealed a significant increase in the staining of abnormal corneal and conjunctival epithelial cells in DED among saline-treated mice throughout the treatment cycle. However, there was a slight improvement in the DPS and PHP-DPS NPs treatment groups, with further decrease after INS treatment. Notably, the PHP-DPS@INS NPs treatment group exhibited a significant decrease in staining at day 6, which became almost invisible by day 12 (Fig. 6b, f).The treatment of conjunctival goblet cells further confirmed the aforementioned conclusions. PAS staining revealed a significant decrease in the number of conjunctival goblet cells in the DED group compared to the normal group, while there was a gradual increase in the number of conjunctival goblet cells in the DPS treatment group, PHP-DPS NPs treatment group, and INS treatment group. The number of conjunctival goblet cells in the PHP-DPS NPs treatment group had returned to nearly normal levels (Fig. 6d, j).This clear observation underscores the significant reparative effects of PHP-DPS@INS NPs on corneal epithelial and conjunctival goblet cell damage associated with DED.

#### Repair effect of PHP-DPS@INS NPs on tear secretion

The phenol-carmine cotton thread test is employed to assess tear secretion. Tear secretion was stimulated in the DPS NPs group (6.70 ± 0.11 mm), PHP-DPS NPs group (6.87 ± 0.21 mm), and INS group (6.97 ± 0.21 mm) at 12 days, albeit with limited efficacy; however, the phenolphthalein cotton line discoloration length of the PHP-DPS@INS NPs group (8.53 ± 0.35 mm) was significantly longer than that of the Saline group (5.07 ± 0.15 mm) (*P* < 0.01) (Fig. 6g). Among all treatment groups, PHP-DPS@INS NPs exhibited superior effectiveness in restoring tear secretion.

#### Repair effect of PHP-DPS@INS NPs on tear film stability

The instability of the tear film is a critical characteristic of DED, and assessing tear film stability is effectively done by measuring tear tBUT [67]. After 12 days of various treatments, the tBUT of the Saline group did not exhibit significant improvement (0.93 ± 0.15s). However, partial restoration of tear film stability was observed in the DPS NPs group, PHP-DPS NPs group, and INS group (tBUT: 4.83 ± 0.06s, 4.95 ± 0.02s, and 5.16 ± 0.15s respectively). The tBUT of the PHP-DPS@INS NPs group showed a significant increase (5.73 ± 0.15s), differing statistically from that of the Saline group (*P* < 0.01) (Fig. 6h). These findings highlight that PHP-DPS@INS NPs can significantly enhance tear film stability.

#### Effect of PHP-DPS@INS NPs on intraocular pressure (IOP)

Glucocorticoids are commonly used as anti-inflammatory agents for DED; however, a common adverse effect is the elevation of IOP. Therefore, we investigated the effect of each treatment group on intraocular pressure. During the 12-day treatment period, IOP in each treatment group exhibited fluctuations, potentially attributed to inflammatory stimulation and strain during manipulation; however, they consistently remained within the normal range (Fig. 6i).

Following a 12-day treatment period, PHP-DPS@INS NPs effectively repaired corneal epithelial defects, increased conjunctival goblet cells, enhanced tear film stability, and restored tear secretion in a mouse model of DED. Importantly, these therapeutic effects were achieved without elevating intraocular pressure, demonstrating remarkable efficacy.

### The underlying mechanism of the therapeutic effect of PHP-DPS@INS NPs in vivo

In our cellular experiment, we observed that the therapeutic efficacy of PHP-DPS@INS NPs might be attributed to GPX4 protein activation, resulting in reduced glutamate and fumarate levels. Subsequently, our goal was to further elucidate the therapeutic mechanism of PHP-DPS@INS NPs in an in vivo setting.

#### Histomorphological study

**Fig. 7:**
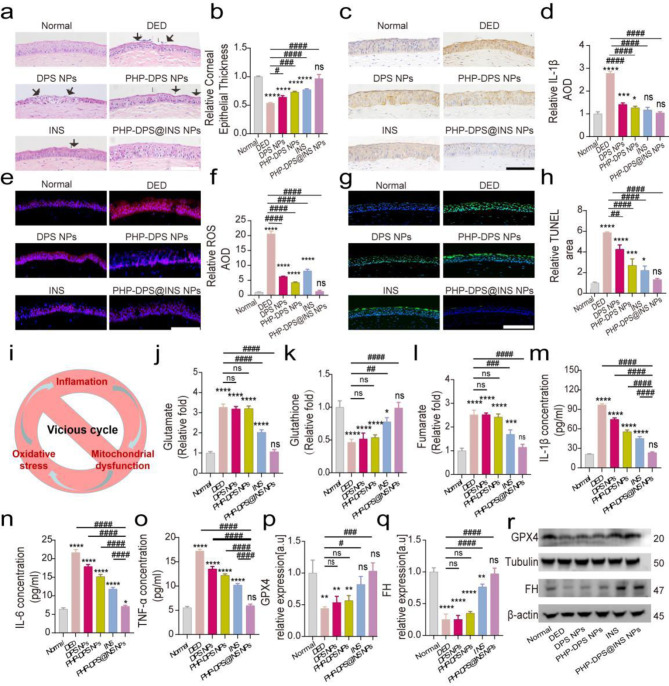
Histological analysis and the potential mechanism of PHP-DPS@INS NPs in the treatment of BAK-induced DED mice model. All the tests were performed on the 12th day of treatment. (**a**) Representative H&E images of the corneal epithelial (black arrow: corneal epithelial damage; scale bar:50 μm). (**b**) Comparison of corneal thickness. (**c**) The image (scale bar:50 μm) and (**d**) Quantitative analysis of IL-1βimmunohistochemical staining of corneal tissues. Representative immunofluorescence staining images of (**e**) oxidative stress (ROS) and (**g**) Apoptosis (TUNEL) in corneal tissues (scale bar:50 μm). Quantification of fluorescence intensity of (**f**) ROS and (**h**) Apoptotic area. (**i**) Mechanism diagram of PHP-DPS@INS NPs in the treatment of DED. (**j**) Glutamate t, (**k**) Glutathione, and (**l**) Fumarate concentration in corneal tissues. The concentration of (**m**) IL-1, (**n**) IL-6 and (**o**) TNF-α in corneal tissues. (**r**) Representative Western Blotting images of GPX4 AND FH expressed in corneal tissues. Quantitative analysis of WB bands about relative expression of GPX4 (**p**) and FH (**q**). Data are presented as mean ± SD; *n* = 3. *P < 0.05, **P < 0.01, ***P < 0.001, and ****P < 0.0001 compared with the normal group; ^#^*p* < 0.05, ^##^*p* < 0.01, ^###^*p* < 0.001, and ^####^*p* < 0.0001 compared with the DED group. DED group: the eye of balb/c mice received 0.2% benzalkonium chloride, 5ul at a time, twice daily (8:00am and 8:00 Pm) for 14 days; DPS NPs(262.5 µg/ml), PHP-DPS NPs(315 µg/ml), INS (35 µg/ml), and PHP-DPS@INS NPs(350 µg/ml) group: BAK-induced DED mice received the corresponding treatments for 12 days ,5ul at a time, twice daily. GPX4: glutathione peroxidase; FH: fumarate hydratase

In the Normal group, the corneal epithelium appeared intact and well-organized, comprising 5–6 layers of epithelial cells. In the DED group, similar to variations in the clinical DED, the superficial corneal epithelium exhibited exfoliation and unevenness, coupled with disordered and significantly thinner epithelial cells (53.85% of the normal group). Following treatment with DPS NPs, although the corneal epithelium remained thin (64.15% of the normal group), there was still significant disarrangement of epithelial cells. Treatment with PHP-DPS NPs or INS enhanced the recovery as evidence by the increased number of corneal epithelial layers (73.07% and 77.27% of the normal group, respectively), yet an irregular arrangement persisted. The corneal epithelium satisfactorily returned to normal thickness (96.70% of the normal group) within 12 days only in the PHP-DPS@INS NPs group. This was accompanied by complete restoration of a well-ordered arrangement of epithelial cells (Fig. 7a, b).

#### Anti-inflammatory effect of PHP-DPS@INS NPs in corneal tissue

For corneal immunohistochemical staining, we chose IL-1β due to its status as one of the most prominent inflammatory signals in the body [68], as well as being a therapeutic target for various inflammatory diseases. The results showed that IL-1β staining intensity was higher in the DED group relative to that of the normal group. The expression level of IL-1β in the cornea was reduced by all treatments, with INS and PHP-DPS@INS NPs exhibiting the most significant inhibitory effect on the levels of inflammatory factor IL-1β, causing a decrease of 57.75% and 62.48%, respectively, compared to the DED group (Fig. 7c, d).

#### Anti-ROS effect of PHP-DPS@INS NPs in corneal tissue

The corneal tissue was subjected to ROS immunofluorescence staining (red fluorescence). A significantly increased red fluorescence was observed in the DED group compared to the normal group. Following 12 days of various treatments, there was a reduction of 69.60%, 79.32%, and 60.43% in red fluorescence intensity in the DPS NPs group, PHP-DPS NPs group, and INS group, respectively, compared to the DED group (Fig. 7e, f). Fluorescence staining of DED corneas revealed a significant decrease in ROS production after a 12-day treatment with PHP-DPS@INS nanoparticles.

#### Effects of PHP-DPS@INS NPs on apoptosis of corneal tissue

The TUNEL staining assay was performed to detect apoptosis in the corneal epithelium. Compared to the normal group, the number of apoptotic cells (green fluorescence) was higher in the DED group. After 12 days of treatment, the number of TUNEL-positive cells in the DPS NPs group, PHP-DPS NPs group, and INS group decreased by 27.36%, 53.97%, and 62.42% respectively, compared to the DED group; however, significant corneal green fluorescence could still be detected. The PHP-DPS@INS NPs group exhibited the lowest number of TUNEL-positive cells in the corneal tissue, with an 77.43% decrease compared to the DED group (Fig. 7g, h), indicating the superior anti-apoptotic ability of PHP-DPS@INS NPs in DED corneal tissue compared to other treatment groups.

#### PHP-DPS@INS NPs can alleviate DED symptoms by inhibiting fumarate levels in vivo

The corresponding assay kits were employed to determine the levels of glutamate, glutathione, and fumarate in corneal tissue. Results indicated that the levels of glutamate and fumarate were increased, while that of glutathione were reduced in the corneal tissue. Conversely, both the INS group and PHP-DPS@INS NPs group exhibited a substantial reduction in glutamate and fumarate levels, along with an augmented presence of glutathione within DED corneal tissue (Fig. 7j-l). The level of inflammatory factors in the corneal tissue was evaluated through the ELISA. Notably, IL-1β, IL-6, and TNF-α levels were increased in the DED group. In the treated groups, inflammatory factors were decreased, with PHP-DPS@INS NPs exhibiting the most potent anti-inflammatory effect, followed by INS, PHP-DPS NPs, and DPS NPs (Fig. 7m-o). Consistent with our in vitro findings, formulations that incorporated INS exhibited superior anti-inflammatory activity compared to those lacking INS.WB results demonstrated significant inhibition of GPX4 and FH expressions in the DED group. Administration of PHP-DPS NPs and DPS NPs did not yield a significant improvement in the expressions of GPX4 and FH. Conversely, treatment involving INS and PHP-DPS@INS NPs led to an upregulation in the expressions of GPX4 and FH within the corneal tissue, with the PHP-DPS@INS NPs group demonstrating a more pronounced effect (Fig. 7p-r).

The histological results affirm the effectiveness of PHP-DPS@INS NPs in disrupting the detrimental “inflammation-oxidative stress-mitochondrial dysfunction” cycle in DED (Fig. 7i). PHP-DPS@INS NPs efficiently restored the thinning and exfoliation of corneal epithelium induced by DED, significantly attenuated abnormal accumulation of inflammatory factors and ROS in the corneal tissue, and reduced the apoptotic cell population within the cornea. Consistent with our in vitro findings, the anti-inflammatory potential of PHP-DPS@INS NPs was attributed to GPX4 activation, leading to a decrease in the inflammatory metabolite—fumarate accumulation and restoration of FH activity.

### In vivo safety assessment

**Fig. 8:**
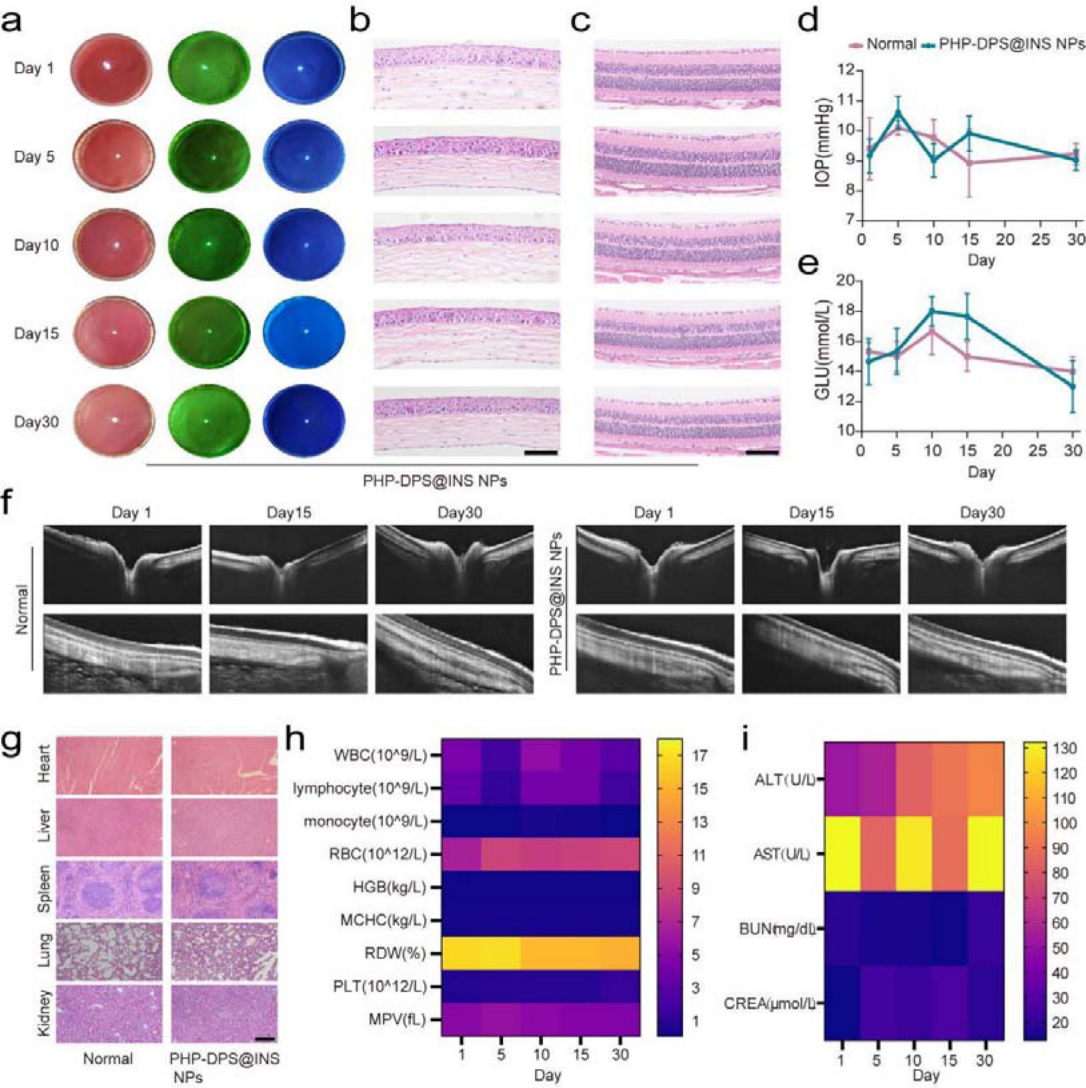
Biosafety of 30-day PHP-DPS@INS NPs therapy in vivo. The ocular surface of mice treated with PHP-DPS@INS NPs was measured by (**a**) slit lamps examination, H&E staining images of (**b**) cornea and (**c**) retina(scale bar:50 μm), and (**d**) intraocular pressure (IOP) measurement on 1,5,10,15 and 30 days. (**f**) OCT images of the structure and morphology of optic papilla and local retina of New Zealand white rabbits. (**g**) Representative H&E staining images of heart, liver, spleen, lung, and kidney on the 30th day of PHP-DPS@INS NPs therapy (scale bar:50 μm). (**e**) Blood glucose levels, (**h**) whole blood parameters and (**i**) Serum levels of ALT, AST, BUN and CRE of mice treated with PHP-DPS@INS NPs on 1, 5, 10, 15 and 30 days. Data are presented as mean ± SD; *n* = 3

Given the favorable therapeutic effects of PHP-DPS@INS NPs demonstrated in both in vitro and in vivo dry eye models, we conducted an in vivo safety evaluation to explore the potential application value and translational prospects of this formulation. PHP-DPS@INS NPs were administered to the eyes twice a day, 5 µl each time, and were evaluated on days 1, 5, 10,15, and 30, respectively. The cornea appeared transparent and smooth under the white light of the slit lamp, with no neovascularization observed under the under the green light. Following sodium fluorescein staining, there were no indications of epithelial defects or ulcerations observed under the cobalt blue light (Fig. 8a). HE staining showed that the number and arrangement of corneal epithelial cells was normal (Fig. 8b). The histological and structural examination of the retina was conducted using HE staining and OCT. The results from HE staining indicated that the number of retinal cells in each layer of mice was within normal range, with clearly stratified layers and normal thickness(Fig. 8c). Additionally, OCT results revealed that compared to normal group, the structure of the optic disc and peripheral retina in PHP-DPS@INS NPs group appeared clear with normal thickness, devoid of hyperplasia, defects or lesions(Fig. 8f). Moreover, IOP exhibited normal fluctuations with any abnormal increase (Fig. 8d). Given the widespread use of INS as a hypoglycemic hormone, we also assessed the impact of PHP-DPS@INS NPs on blood glucose levels in mice over 30 days following ocular administration and observed no abnormal effects (Fig. 8e).HE staining analysis was conducted on the major organs of the mice (heart, liver, spleen, lung, and kidney. When compared with normal mice, no histopathological lesions were found (Fig. 8g). The mice underwent routine blood tests and assessment of liver and kidney function, with the obtained blood measurements falling within the normal range (Fig. 7g-i, Table S6-7). These results suggest that PHP-DPS@INS NPs have favorable in vivo safety profile and the potential to treat DED.

## Conclusion

DED is a prevalent ocular surface disease globally, significantly impacting patients’ quality of life and imposing substantial economic burdens on society. The challenges associated with ocular drug delivery and the intricate etiology of DED have resulted in a scarcity of effective treatment methods for this condition. Consequently, we developed a multifunctional nanosystem. To overcome the bottleneck of ocular delivery, we successfully modified the nanoparticles to reverse their charge. Cationic nanoparticles display improved adherence to the anionic corneal surface, promoting endocytosis. Upon internalization of PHP-DPS@INS NPs into lysosomes in the acidic environment, the pH-sensitive urea bond within the nanoparticle shell undergoes cleavage at a specific site, resulting in the release of PEI with a proton sponge effect. The mechanism ultimately enables efficient escape of nanoparticles from lysosomes and subsequent entry into the cytoplasm. Using high-throughput targeted metabolomics, we identified that the anti-inflammatory effect of INS in the hypertonic HCEC model was associated with a reduction in the mitochondrial inflammatory metabolite-fumarate. When compared to individual treatments, the anti-inflammatory effect of INS synergistically combined with the antioxidant effect of the SS-31 peptide, effectively inhibiting the elevation of inflammatory factors and ROS levels. Moreover, it restored mitochondrial membrane potential and other crucial functions while improving mitochondrial structure. This synergistic approach disrupts the detrimental cycle of “inflammation-oxidative stress-mitochondrial dysfunction” in DED. In vivo experiments revealed that PHP-DPS@INS NPs more efficiently restored corneal epithelium integrity, increase the number of conjunctival goblet cells, improved tear film stability, promoted tear secretion, and had excellent biological safety. In conclusion, this study presents a novel, safe, and efficacious therapeutic strategy for the management of DED. Importantly, we show that the PHP-DPS@INS NPs treatment system can inhibit inflammation by modulating mitochondrial metabolites, suggesting that it is a promising approach for the treatment of inflammatory disorders associated with DED.

## Experimental section

### Materials

Human recombinant Insulin (INS), Benzalkonium Chloride (BAC), and Sodium Chloride (NaCL) were purchased from Sigma-Aldrich (St. Louis, MO, USA). The SS-31 peptide was purchased from China Peptide Biotechnology (Shanghai, China). Cholesterol, 1,2-Dipalmitoyl-Snglycero-3-Phosphocholine (DPPC), and 1,2-stearoyl-sn-glycerol-3-Phosphoethanolamine-N-[methoxy (polyethylene glycol)-2000] (DSPE-mPEG2000) were purchased from AVT Pharmaceutical Technology (Shanghai, China). Polyethyleneimine (PEI), DSPE-PEG2k-NHS, and PEG2000-NH2 were purchased from Ruixi Biotechnology (Xi ‘an, China). The Cell Counting Kit-8 (CCK-8), the Calcein-AM/PI double stain kit, the JC-1 assay kit, Mitotracker red, Lysotracker red, the MPTP Assay Kit, 2,7-Dichlorodihydrofluorescein Diacetate (DCFH-DA), 1,1′-dioctadecyl-3,3,3′,3′-tetramethylindocarbocyanine perchlorate (DiI), and 4,6-Diamidino-2-Phenylindole (DAPI) were purchased from Biyuntian (Shanghai, China). The Fumarate Assay Kit and the Glumatate assay kit (MAK060) were purchased from Sigma-Aldrich (St. Louis, MO, USA), and NOVUS (Littleton, CO, USA), respectively. The Glutathione ELISA kit (E-EL-0026c) was purchased from Elabscience (Wuhan, China). The IL-1β, IL-6, and TNF-α ELISA kits were purchased from BioLegend (California, USA). The recombinant human insulin ELISA kit was purchased from Soleibo Biotechnology (Beijing, China). The GPX4 (52455) and fumarate hydratase (ab233394) antibodies were purchased from CST (Danvers, MA, USA) and Abcam (Cambridge, UK), respectively. Chloroform (CHCl_3_) used herein was synthesized by Chuandong Chemical (Chongqing, China). The phenol red cotton thread was purchased from Zone-Quick (Tokyo, Japan). All other reagents were at least of analytical grade.

### DSPE-PEG2000-SS31 synthesis

First, DSPE-PEG2k-NHS (100 mg) was dissolved in 3 ml DMF. For complete dissolution, the SS-31 peptide (1.1 equivalents) and triethylamine (3.0 equivalents) were added, and the reaction was conducted at Room Temperature (RT) for 12 h. Subsequently, the reaction solution was transferred to a dialysis bag (MW: 2000 Da) and dialyzed in pure water for 24 h. The dialysate was then collected and freeze-dried to obtain DSPE-PEG2000-SS31. Finally, the molecular structure of DSPE-PEG2000-SS31 was identified using ^1^H-NMR (400 MHz, Bruker, Germany).

### PEG2000-PEI synthesis

PEG2000-NHS (0.5 g) was dissolved in 10 ml of chloroform, followed by the addition of PEI1.8k (1.0 eq.) to achieve complete dissolution. After a 0.5 h reaction at room temperature, the solvent was removed by vacuum evaporation, and the resulting product was dissolved in 5 ml of methanol. Subsequently, the product was placed in a dialysis bag with a molecular weight cutoff of 2000 and dialyzed in pure water for 24 h. The dialysate was collected and freeze-dried to obtain the PEG2000-PEI product.

### PEG2000-Hyd-PEI synthesis

First, 1 g PEG2000-NH_2_ was dissolved in 10 mL chloroform to obtain PEG2000-Hyd-OH. Subsequently, HO-Hyd-COOH (1.1 eq.), EDC (2.0 eq.), and DMAP (0.1 eq.) were added, and the reaction was performed at RT for 8 h. The reaction solution was concentrated through vacuum rotary evaporation, precipitated in a large amount of glacial ether, filtered, collected, and then subjected to vacuum drying. The resulting PEG2000-Hyd-OH (1 g) was dissolved in 10 mL ultra-dry acetonitrile supplemented with di(p-nitrophenyl) carbonate (1.0 eq.) and triethylamine (5.0 eq.), and then reacted at 50 °C for 5 h. Following that, a PEI (1.0 eq.) solution dissolved in 5 mL chloroform was added and the mixture was reacted at RT for 0.5 h. After removing the solvent through vacuum rotary evaporation, 5 mL methanol was added to dissolve the product, which was then placed in a dialysis bag (MW: 2000) and dialyzed in pure water for 24 h. The dialysate was collected and freeze-dried to obtain PEG2000-Hyd-PEI1.8 K. The molecular structure of PEG2000-Hyd-PEI was determined using ^1^H-NMR (400 MHz, Bruker, Germany).

### PHP-DPS@INS NPs synthesis

*T*he reverse evaporation method was used to produce PHP-DPS@INS NPs. First, 20 mg of DPPC, cholesterol, and DSPE-PEG2000-SS31 (mass ratio = 3:1:1) were dissolved in 10 ml chloroform, and then a 2 ml INS solution (4 mg/ml) was added. The W/O colostrum was formed through ultrasonic shock treatment for 5 min. Subsequently, the round bottom flask was placed on a rotary evaporator, and CHCl_3_ was removed through vacuum evaporation (50 rpm, 30 °C, 1 h) until the liposome gel formed. Next, the liposome gel was detached by adding normal saline. It was then re-fixed in a rotary evaporator (50 rpm, 30 °C, 30 min) to obtain the DPS@INS NP suspension. Subsequently, the suspension was slowly shaken and incubated for 1 h after adding 4 mg PEG2000-Hyd-PEI. It was then sonicated for 5 min in an ice water bath (35 W, on and off intervals of 5 s each, for 5 min) using an ultrasonicator (Sonics & Materials Inc., USA). Subsequently, PHP-DPS@INS NPs were obtained by extruding through a polycarbonate membrane using a 220 nm aperture. On the other hand, PHP-DPS NPs were prepared using the same protocol as PHP-DPS@INS NPs but without INS loading. Additionally, DPS NPs were obtained following the same scheme as DPS@INS NPs but without loading INS. Through the same scheme as DPS@INS NPs, DP@INS NPs were obtained by replacing DSPE-PEG2000-SS31 with DSPE-PEG2000.

### DiO or dii labeled nanoparticles synthesis

20 mg of DPPC, cholesterol, and DSPE-PEG2000 (mass ratio = 3:1:1) were dissolved in 10 ml of chloroform. Subsequently, 2 ml of INS solution (4 mg/ml) was added. After undergoing ultrasound impact treatment for 5 min, W/O colostrum was formed. Then, 0.5 mg of DiI or DiR was introduced into the mixture before placing the round-bottomed flask on a rotary evaporator to remove chloroform by vacuum evaporation (50 rpm, 30 C, 1 h), resulting in the formation of liposome gel and obtaining DiO or DiI labeled DP@INS NPs;20 mg DPPC, cholesterol, and DSPE-PEG2000-SS31 (mass ratio = 3:1:1) was dissolved in 10 ml chloroform, followed by the addition of 2 ml INS solution (4 mg/ml). After 5 min of ultrasound treatment, a W/O emulsion was formed. Subsequently, 0.5 mg DiI or DiR was added, and the round-bottom flask was subjected to vacuum evaporation on a rotary evaporator (50 rpm, 30 C, 1 h) to remove chloroform and form liposome gel. Then, 4 mg PEG2000-Hyd-PEI was added and the suspension was gently agitated and incubated for another hour to obtain DiO or DiI labeled PHP-DPS@INS NPs. The entire process was conducted under light-free conditions.

### Physicochemical characterization

Particle size, Polydispersity Index (PDI), and zeta potential of PHP-DPS@INS NPs were measured using a Dynamic Light Scattering (DLS) analyzer (Malvern, ZEN3600, UK). The morphology of PHP-DPS@INS NPs was visualized through Transmission Electron Microscopy (TEM, JEM-2100, Japan) with negative staining. Insulin concentration in the solution was determined through Ultra-High Performance Liquid Chromatography (UHPLC, Shimadzu 20, Japan) along with a UV variable wavelength detector and an Ultimate XB-C18 column. We performed UHPLC analysis using a mobile phase comprising water, acetonitrile, and trifluoroacetic acid at a 68.5:31.5:0.1 ratio (flow rate: 1 ml/min, detection wavelength: 220 nm). Encapsulation Efficiency (EE) and Drug Loading (DL) were calculated using the following formulas:

EE (%) = Mass of the encapsulated drug / Total mass of the drug × 100%.

DL (%) = Mass of the encapsulated drug /Mass of total liposomes × 100%.

### In vitro stability

The PHP-DPS@INS NPs were stored at 37 °C. The particle size and Zeta potential of PHP-DPS@INS NPs were measured using DLS over 15 days.

### Measurement of intracellular pH (pHi)

The intracellular pH (pHi) was assessed using 20,70-bis-(2-carboxyethyl)-5-(and-6)-carboxyfluorescein (BCECF). HCECs (1 × 10^4^ cells) were seeded in a laser confocal cell dish and cultured overnight as the control group. For the HOS group, the original medium was replaced with 450 mOsM medium, and the cells were incubated for 12 h. After discarding the medium, 1 ml of pre-prepared BCECF probe solution was added for 30 min. The supernatant was removed, and the cells were washed with PBS three times. The BCECF fluorescence in HCECs was observed by CLSM. Following trypsin digestion and resuspension with PBS, BCECF levels were measured by flow cytometry at an excitation wavelength of 488 nm and emission wavelengths of 640 nm and 525 nm.

### Ocular surface pH measurement

The ocular surface pH was measured using precision pH paper. Male Balb/c mice (weighing 18–20 g) were administered daily eye drops containing 0.2% BCA, 5 µl each time, twice a day (8:00 am and 8:00 pm), for 14 days to establish the DED group. Male Balb/c mice (weighing 18–20 g) without any treatment were used as the normal group. After calming the mice, a precision pH test paper was gently touched to the corneal surface, and the pH value was read after 30 s based on the color of the test paper and color card.

### PH responsiveness

The PHP-DPS@INS NPs were mixed with pure water with different pH values (pH 4.8, 5.8, and 7.4), and the corresponding particle size and zeta potential were evaluated using DLS after 12 h.

### Drug release in vitro

The PHP-DPS@INS NPs were placed in a dialysis bag (MWCO = 3.5 kDa), immersed in 50 mL PBS solutions with different pH values (pH 4.8, 5.8, and 7.4), and continuously stirred at 37 °C. Samples (200 µL) were taken and replaced with an equal volume of fresh medium at each predetermined time. The INS content was measured and analyzed using the above-mentioned UHPLC approach.

### Cell culture and establishment of the hyperosmotic stress (HOS) model

Herein, Human Corneal Epithelial Cells (HCECs) were obtained from the BeNa Culture Collection (BNCC337876, Beijing, China). The cells were cultured in a DMEM medium (Gibco, USA) supplemented with 10% Fetal Bovine Serum (FBS; Gibco, USA) and 1% penicillin-streptomycin. The HOS model of HCECs was induced by adding 69 mM NaCl to the DMEM medium to raise the medium’s osmotic pressure to 450 mOsM. The cells were cultured at 37 °C, 5% CO^2^, and 90% Relative Humidity (RH).

### In vitro toxicity and treatment conditions

The CCK-8 assay: Normally cultured HCECs were incubated with different concentrations of INS (2.5, 5, 10, 20, 40, 80, 160, and 320 µg/ml) and PHP-DPS@INS NPs (25, 50, 100, 200, 400, 800, 1600, and 3200 µg/ml) in a serum-free medium for 24 h. Additionally, the same concentrations of PHP-DPS@INS NPs were added to HOS-cultured HCECs. A newly prepared CCK-8 solution was added to each well after 6, 12, and 24 h of incubation. The absorbance of the reaction product at 450 nm was measured using a microplate reader (LabServ ®, Thermo Fisher Scientific) to determine the in vitro Cell Survival Rate (CSR). The optimal therapeutic concentration was selected based on the CCK-8 assay results.Calcein-AM/PI staining: HOS-cultured HCECs were incubated with PHP-DPS@INS NPs (200 µg/ml), and the negative control (normal) and positive control (HOS) groups were set up. The medium was discarded at 6, 12, and 24 h, and the cells were washed twice with PBS. The cells were then incubated with the pre-configured Calcein-AM/PI staining reagent at 37 ° C for 30 min. The samples were observed under a Confocal Laser Scanning Microscope (CLSM; Nikon, Tokyo, Japan).

### PHP-DPS@INS NP uptake in HOS-cultured HCECs

Herein, HCECs (1 × 10^4^ cells) were seeded overnight in a laser confocal cell culture dish. After discarding the waste medium, the cells were incubated with a 450 mOsM medium for 12 h. They were then incubated with DiI-DP@INS NPs and DiI-PHP-DPS@INS NPs (200 µg/ml). Subsequently, the cells were washed with PBS and fixed with 4% paraformaldehyde at predetermined time points (1, 2, and 3 h). The nuclei were then stained with DAPI. Intracellular fluorescence was observed using CLSM and the cellular uptake behavior was determined through flow cytometry.

### Lysosomal escape and mitochondrial targeting in HOS-cultured HCECs

First, HCECs (1 × 10^4^ cells) were seeded in a laser confocal cell culture dish and cultured overnight. The cells were then incubated with the 450 mOsM medium for 12 h after discarding the waste medium. Next, the cells were incubated with DiO-DP@INS NPs and DiO-PHP-DPS@INS NPs (200 µg/ml), with three samples in each group. The medium was discarded, and the cells were washed with PBS three times at predetermined time points (1, 2, and 3 h). In each dish, a lysosomal red fluorescent probe (500 µL) or mitochondrial red fluorescent probe (400 µL) was added and the mixture was incubated in a cell thermostatic incubator for 35 min. Subsequently, the supernatant was discarded, and the cells were rinsed with PBS three times. Each well was incubated with 1 ml DAPI for 15 min, and rinsed with PBS three times. After rinsing, the cells were fixed in a 500 µl paraformaldehyde solution. Lysosomal localization and mitochondrial localization of NPs in HCECs were observed using CLSM.

### ROS measurement

The intensity of DCF fluorescence can reflect ROS levels in cells. Herein, HCECs (1 × 10^4^ cells) were seeded overnight in a laser confocal cell culture dish. After removing the waste medium, the 450 mOsM medium was added and the mixture was incubated for 12 h. Thereafter, DPS NPs (150 µg/ml), PHP-DPS NPs (180 µg/ml), INS (20 µg/ml), and PHP-DPS@INS NPs (INS: 20 µg/ml, PHP-DPS: 180 µg/ml) were added to each group and incubated for 12 h. Subsequently, 1 ml DCFH-DA solution was added to each dish and incubated for 20 min. The supernatant was discarded, and the cells were rinsed with PBS three times. The ROS fluorescence in HCECs was observed through CLSM. The DCF fluorescence of each sample was measured through flow cytometry at 488 and 525 nm excitation and emission wavelengths, respectively.

### High-throughput target metabolomics detection and analysis

First, HCECs were placed in a 100 mm petri dish overnight and then incubated with the 450 mOsM medium for 12 h (the HOS group). Simultaneously, another sample was treated with 20 µg/ml insulin for 12 h (the INS group). On the other hand, normally cultured HCECs were set as the normal group. Each group had four samples. The metabolites in the appeal samples were detected and analyzed through high-throughput target metabolomics. Briefly, the LC-MS/MS method was used to accurately analyze 600 metabolites in the appeal samples qualitatively and quantitatively. The 600 metabolites include metabolites of amino acid metabolism, as well as the TCA, glycolysis, and pentose phosphate pathways, among other pathways. Chromatographic conditions: An ACQUITY UPLC H-Class (Waters) UPLC system equipped with a Waters Atlantis Premier BEH ZHILIC column (2.1 mm * 150 mm, 1.7 μm) was used. Phase A comprised ultrapure water and acetonitrile (8:2) supplemented with 10 mmol/L acetic acid, whereas phase B comprised ultrapure water and acetonitrile (1:9) also supplemented with 10 mmol/L acetic acid. The pH of the A and B mobile phases was adjusted to 9 using ammonia water before performing gradient elution. The column temperature was maintained at 40 °C throughout the analysis, and each sample received 1 µL injection volume. The Ion Drive Turbo V source operating in a positive ion mode (ESI+) and Multiple Reaction Monitoring (MRM) were employed for Mass Spectrometry (MS) analysis. The source temperature was maintained at 500 °C, and the source voltage was set to + 5000 V/-4500 V. A Curtain Gas pressure of 35 PSI (4.67 KPa) was also applied. All MS data acquisitions and quantitative analyses of target compounds were performed using SCIEX Analyst Work Station Software (v1.7.2) and Data-Driven Flow software (v-1.0.1).

### PHP-DPS@INS NPs inhibited the inflammatory response of HOS-cultured HCECs

The waste medium of HOS-cultured HCECs was replaced by the serum-free medium of DPS NPs (150 µg/ml), PHP-DPS NPs (180 µg/ml), INS (20 µg/ml), and PHP-DPS@INS NPs (INS: 20 µg/ml, PHP-DPS: 180 µg/ml) and incubated for 12 h. After incubation, the Glutamate (GLU), Glutathione (GSH), and Fumarate levels in cells were quantified using commercially available kits per the manufacturers’ instructions. The cell supernatant was examined per the IL-1β, IL-6, and TNF-α ELISA kit protocols, and the absorbance of each hole was measured using a microplate reader (BioTek Instruments Inc.). The cells were lysed with a RIPA lysis buffer (Sigma-Aldrich, St. Louis, MO, USA) and incubated on ice for 15 min. The supernatant containing the target proteins was then collected and centrifuged at 14,000 rpm for 15 min. The protein levels were quantified through the Bradford assay. The lysate samples were loaded onto channels and separated by Sodium Dodecyl Sulphate-Polyacrylamide Gel Electrophoresis (SDS-PAGE). The resulting protein bands were transferred to Polyvinylidene Fluoride (PVDF) membranes (Millipore, USA) for nonspecific binding with 5% BSA. The membranes were first incubated with GPX4 (1:1,000, ab125066, Abcam) and FH/Fumarase (1:1,000,ab233394, Abcam) antibodies, and then with a Horseradish Peroxidase (HRP)-labeled goat anti-rabbit IgG (ab6721, Abcam) or goat anti-mouse IgG (ab205719, Abcam) antibody at a 1: 3000 ratio. The results were analyzed using ImageLab 4.1 software (BioRad).

### Mitochondrial membrane potential

After treatment, 1 ml JC-1 staining solution (1X) was added to each well before incubating for 20 min. The supernatant was discarded, and the cells were rinsed twice with the JC-1 staining buffer (1X). Next, 1 ml DAPI was added for nuclear staining, and the cells were incubated for 15 min. Subsequently, the medium was discarded, and the cells were washed with PBS three times. This was followed by addition of paraformaldehyde (500 µl) to each well, and the red and green fluorescence intensities were observed through CLSM. The JC-1 fluorescence was quantified through flow cytometry.

### MPTP

After treatment, cells in each treatment group were gently washed with PBS once. Calcein AM working solution was then added to the cell suspension and mixed well before incubating in a cell thermostatic incubator for 30 min in the dark. Subsequently, CoCl_2_ was added to each group and mixed well for 15 min. Fluorescence intensity was observed using CLSM.

### Mitochondrial morphology

The samples were sequentially fixed, rinsed, dehydrated, and embedded. They were then sliced into ultra-thin sections (with a thickness of 70–90 nm) using an ultra-thin slicing machine (EM UC7, LEICA, GERMAN). The sections were stained with the lead citrate staining solution and the acetic acid hydrogen peroxide staining solution for 5 min and dried. Mitochondrial morphology was observed through TEM (Tecnai G2 12, FEI, USA), with the acceleration voltage set at 100 kV.

### DED mice model

Male Balb/c mice (weighing 18–20 g) and New Zealand white rabbits (weighing 2.0–2.5 kg) were acquired from the Laboratory Animal Center of Chongqing Medical University. All experimental procedures were conducted per the ARVO Statement on the Use of Animals in Ophthalmology and Vision Research. The Institutional Animal Care Committee of Chongqing Medical University approved the experimental protocol (Ethics number: IACUC-CQMU-2023-0168). All mice were housed at constant temperature (25 ± 1 °C) and humidity (50 ± 10%) with a 12 h: 12 h light-dark cycle. The animals received daily eye drops containing 0.2% BCA, 5 µl each time, twice a day (8:00 am and 8:00 pm), for 14 days.

### PHP-DPS@INS NP distribution and uptake in the anterior segment of the eye

After preparing the corneal frozen sections, the distribution and uptake of PHP-DPS@INS NPs in the anterior segment were observed using an OCT machine (CIRRUS HD 5000, ZEISS, Germany), and a small animal fluorescence imaging system (Viber lourmat, France), respectively. The distribution of eye drops in the cornea was observed through OCT. The New Zealand white rabbits were treated with different eye drops (the DPS NPs group: 262.5 µg/ml, the DPS@INS NPs group: 306.25 µg/ml, the PHP-DPS NPs group: 315 µg/ml, and the PHP-DPS@INS NPs group: 350 µg/ml; 30 µl each time). After 3 min, the ocular surface of the anesthetized New Zealand white rabbits (intraperitoneally injected with 1% pentobarbital sodium, at 50 mg/kg) was detected and recorded through OCT. The retention of each liposome NP in the cornea of DED mice was observed using the small animal fluorescence imaging apparatus. The DiI-DP@INS NPs (350 µg/ml) and DiI-PHP-DPS@INS NPs (350 µg/ml) were applied to DED mice, 5 µl each time, and the fluorescence distribution on their cornea was observed at 0, 1, 5, 10, 20, and 30 min. The frozen sections were examined to determine the penetration of NPs in the corneal epithelium of DED mice. The DED mice were treated with DiI-DP@INS NPs (350 µg/ml) and DiI-PHP-DPS@INS NPs (350 µg/ml), 5 µl each time. The eyeballs of each mouse were completely removed after 2 and 10 min, and then embedded and stored at -80 °C. Subsequently, they were sliced into 7 μm continuous sections using a frozen sectioning machine. The distribution of NPs in the cornea was observed and analyzed using an inverted fluorescence microscope (Eclipse, Ti-S, Nikon, Japan). The experiments were performed in triplicate for each group.

### Insulin pharmacokinetics in aqueous humor

The DED mice were treated with pre-configured INS eye drops (35 µg/ml) and PHP-DPS@INS NPs eye drops (with INS: 35 µg/ml), 5 µl each time, followed by a gentle closure of the eyelids. At 0.05, 0.25, 0.5, 0.75, 1, and 2 h post-treatment, the aqueous humor was promptly extracted from the corneoscleral edge using a micro-injector needle upon euthanizing the mice. The tissue drug concentration was determined using a human recombinant insulin ELISA kit (Solyndra, Beijing) per the kit instructions.

### In vivo efficacy evaluation

Each treatment group’s efficacy was evaluated through corneal fluorescein staining and scoring, bengal red staining and scoring, tBUT detection, tear secretion test, and IOP monitoring. The mice were randomly divided into five groups for observation (4 mice (8 eyes) in each group): Saline, DPS NPs treatment (262.5 µg/ml), PHP-DPS NPs treatment (315 µg/ml), INS treatment (35 µg/ml) and PHP-DPS@INS NPs treatment (350 µg/ml); 5 µl administered each time, twice a day (8:00 am and 8:00 pm). The measurements were taken at the end of modeling (day 0), day 3, day 6, day 9, and the last day of treatment (day 12). Corneal staining was examined using a slit microscope after a local pointing of the eye with a 0.1 mL 0.25% fluorescein sodium dye solution and was observed by manual blinking five times to ensure the fluorescein sodium evenly covered the cornea. The cornea was divided into four quadrants which were individually assessed based on the following scoring criteria: 0 (no staining was seen); 1 (< 30 spots, mild micro-spot staining); 2 (> 30 spots, no diffuse staining); 3 (no positive spots, severe diffuse staining); and 4 (visible fluorescein plaque staining). The scores were determined separately in each quadrant and then added to obtain the total score (Total score range: 0–16 points). The tear film BUT was determined by dripping 0.1 mL 0.25% sodium fluorescein into the conjunctiva of the lower eyelid and manually blinking five times. After the last blink, the time at which the first black dry spot appeared was recorded. The tear secretion test was performed by placing the phenol-carmine thread in the peripheral conjunctival sac of the lateral canthus of mice for 15 s, measuring the length of the stained phenol-carmine thread with a millimeter ruler, and recording the results. Intraocular pressure monitoring was performed using the TonoLab rebound tonometer (Icare, Finland). The probe was vertical and 3–4 mm away from the central part of the cornea. The above measurements were recorded three times for each eye.

### Histopathological evaluation

On the final day of treatment (day 12), mice eyeballs were collected from each treatment group and compared to the normal control and dry eye model groups. Formalin-fixed eyeballs were embedded in paraffin and sectioned into 5 μm thick slices for H&E and PAS staining. Immunohistochemical slices were labeled with a primary antibody overnight at 4 °C before incubating with a secondary antibody at 21 °C for 1 h. We performed ROS corneal tissue fluorescence staining using a ROS probe added to the samples and incubated at 37 °C for 40 min without light exposure. After washing and sealing, the slices were observed under a fluorescence microscope, and images were captured. Apoptosis was detected through Terminal Deoxynucleotidyl Transferase-mediated Nick End Labeling (TUNEL) staining, and the nuclei were restained with DAPI before being observed under a fluorescence microscope.

### Exploration of in vivo therapeutic mechanisms

After removing the contents, the corneal tissue was preserved at -80 °C. The corneal tissue was carefully separated, homogenized with the RIPA lysate, and centrifuged at 15,000 rpm for 15 min. Following the manufacturers’ instructions, commercial kits were used to quantify the GLU, GSH, and fumarate levels. The levels of intracorneal inflammatory markers (IL-1β, IL-6, and TNF-α) were determined using an ELISA kit. Section 2.6.3 details the WB method used.

### In vivo safety assessment

The ocular safety of PHP-DPS@INS NPs (350 µg/ml) was assessed by bilateral instillation of 5 µl eye drops twice daily on days 1, 5, 10, 15, and 30. Under a slit lamp, the mice corneas were examined using different light sources: white, green, and cobalt blue (combined with sodium fluorescein staining) lights for overall corneal morphology assessment, corneal vessel observation, and detecting corneal epithelial defects, respectively. These observations were recorded. Corneal histology was evaluated through H&E staining, while retinal structure was examined by HE staining and OCT. Given the limited resolution of our OCT machine for detecting the mouse retina, we opted to conduct retinal OCT examination on normal New Zealand rabbits.Intraocular pressure was measured using the Schiotz tonometer (TV01, Icare, Finland). Blood samples were collected from each group of mice to assess blood glucose levels, perform routine blood analysis, and evaluate liver and kidney function. Additionally, the in vivo safety of the formula was confirmed through histological examination, with H&E staining conducted on the main tissues.

### Statistical analysis

Results were presented as Mean (M) ± Standard Deviation (SD). Statistical analyses were performed using the student’s t-test or one-way Analysis of Variance (ANOVA). Results with *p* ≤ 0.05 were considered statistically significant.

### Electronic supplementary material

Below is the link to the electronic supplementary material.

Supplementary Material 1

## Data Availability

No datasets were generated or analysed during the current study.

## Abbreviations

BAK: Benzalkonium chloride
CCK-8: Cell Counting Kit-8
CHCl3: Chloroform
CLSM: Confocal Laser Scanning Microscope
CSR: Cell Survival Rate
DED: Dry Eye Disease
DPPC: 1,2-Dipalmitoyl-Snglycero-3-Phosphocholine
DSPE-mPEG2000: 1,2-stearoyl-sn-glycerol-3-Phosphoethanolamine-N-[methoxy (polyethylene glycol)-2000
DCFH-DA: 2,7-Dichlorodihydrofluorescein Diacetate
DiI: 1,1′-dioctadecyl-3,3,3′,3′-tetramethylindocarbocyanine perchlorate
DAPI: 4,6-Diamidino-2-Phenylindole
DLS: Dynamic Light Scattering
DL: Drug-loading
EE: Encapsulation efficiency
FH: Fumarate hydratase
GCs: Glucocorticoids
GLU: Glutamate
GSH: Glutathione
GPX4: Glutathione peroxidase
Hyd: Hydride
HCEC: Human Corneal Epithelial Cell
HOS: Hyperosmolar stress
H&E: Hematoxylin and Eosin
INS: Insulin
IGFBP-3: Insulin-like Growth Factor Binding Protein-3
INSR: Insulin Receptors
IGF-1R: Insulin-like growth factor 1 receptor
IGF-1: Insulin-like growth factor 1
IOP: Intraocular pressure
MPTP: Mitochondrial Permeability Transition Pore
MRM: Multiple Reaction Monitoring
MS: Mass Spectrometry
M: Mean
NPs: Nanoparticles
NaCL: Sodium Chloride
OS: Oxidative Stress
OCT: Optical coherence tomography
OPLS-DA: Orthogonal projections to latent structures- discriminant analysis
PEI: Polyethyleneimine
PVDF: Polyvinylidene Fluoride
QoL: Quality of Life
ROS: Reactive Oxygen Species
SDS-PAGE: Sulphate-Polyacrylamide Gel Electrophoresis
SD: Standard Deviation
TCA: Tricarboxylic Acid
tBUT: tear Break Up Time
TUNEL: Terminal Deoxynucleotidyl Transferase-mediated Nick End Labeling
UHPLC: Ultra-High Performance Liquid Chromatography

## References

[1] Clayton JAD, Eye (2018). N Engl J Med.

[2] Yu J, Asche CV, Fairchild CJ (2011). The economic burden of dry eye disease in the United States: a decision tree analysis. Cornea.

[3] Zhang Y, Qi Y, Li Y, Zhang Y, Zhao Y (2024). Han,H.;Wang,Y.Engineered assemblies from isomeric pentapeptides augment dry eye treatment. J Control Release.

[4] Rhee MK, Mah FSI. in Dry Eye Disease: How Do We Break the Cycle? Ophthalmology. 2017,124,S14-S19. 10.1016/j.ophtha.2017.08.029 29055357

[5] Nagai N. Otake H. Novel drug delivery systems for the management of dry eye. Adv Drug Deliv Rev. 2022,191114582. 10.1016/j.addr.2022.114582 36283491

[6] Harrington JS, Ryter SW, Price DR (2023). ,A.M.K.Mitochondria in health, disease, and aging. Physiol Rev.

[7] Zorov DB, Juhaszova M, Sollott SJ. Mitochondrial reactive oxygen species (ROS) and ROS-induced ROS release. Physiol Rev. 2014;94:909–50. 10.1152/physrev.00026.2013 PMC4101632 24987008

[8] Zecchini V, Paupe V, Herranz-Montoya I, Janssen J, Wortel IMN, Ferguson A, Chowdury SR, Segarra-Mondejar M, Costa ASH, Pereira GC, Tronci L, Young T, Nikitopoulou E, Yang M, Bihary D, Caicci F, Nagashima S, Speed A, Bokea K. Fumarate induces vesicular release of mtDNA to drive innate immunity. Nature. 2023;615:499–506. 10.1038/s41586-023-05770-w PMC10017517 36890229

[9] Hooftman A, Peace CG, Ryan DG, Day EA, Yang M, McGettrick F, Yin M, Montano EN, Huo L, Toller-Kawahisa JE, Ryan TAJ, Bolado-Carrancio A, Casey AM, Prag HA, Costa ASH, Venuturupalli DJ. De Los Santos G, Ishimori M, Wallace S. Macrophage fumarate hydratase restrains mtRNA-mediated interferon production. Nature. 2023;615:490–8. 10.1038/s41586-019-0000-0 PMC10411300 36890227

[10] Ouyang W, Wang S, Yan D, Wu J, Zhang Y, Li W. Hu,Ji.;Liu,Z.The cGAS-STING pathway-dependent sensing of mitochondrial DNA mediates ocular surface inflammation. Signal Transduct Target Ther. 2023;21371. 10.1038/s41392-023-01624-z PMC10514335 37735446

[11] Chi W, Hua X, Chen X, Bian F, Yuan X, Zhang L, Wang X, Chen D, Deng R, Liu ZLY, de Paiva CS (2017). Li,D.;mitochondrial DNA oxidation induces imbalanced activity of NLRP3/NLRP6 inflammasomes by activation of caspase-8 and BRCC36 in dry eye. J Autoimmun.

[12] Park K, Li,Qi.;Evcimen ND, Rask-Madsen C, Maeda Y, Maddaloni E, Yokomizo H, Shinjo T, St-Louis R, Fu,J.;Gordin,D.;Khamaisi,M.;Pober D;KeenanH (2018). King,G.L.Exogenous insulin infusion can decrease atherosclerosis in Diabetic rodents by improving lipids, inflammation, and endothelial function. Arterioscler Thromb Vasc Biol.

[13] Yassin M, Sadowska Z, Tritsaris K, Kissow H, Hansen CHF, Forman JL, Troelsen JT, Pedersen AE (2018). Olsen,J. rectal insulin Instillation inhibits inflammation and Tumor Development in Chemically Induced Colitis. J Crohns Colitis.

[14] Chang Y, Hung L, Chen Y, Wang W, Lin C, Tzeng H, Suen J, Chen Y. Insulin reduces inflammation by regulating the activation of the NLRP3 inflammasome. Front Immunol. 2021:11587229. 10.3389/fimmu.2020.587229 PMC7933514 33679687

[15] Diaz-Valle D, Burgos-Blasco B, Rego-Lorca D, Puebla-Garcia V, Perez-Garcia P, Benitez-Del-Castillo JM, Vicario-de-la-Torre M, Gegundez-Fernandez JA. Comparison of the efficacy of topical insulin with autologous serum eye drops in persistent epithelial defects of the cornea. Acta Ophthalmol. 2022:e100912–919. 10.1111/aos.14997 34407296

[16] Balal S, Din N, Ashton C, Ahmad S (2023). Healing of Chemical Injury-related persistent corneal epithelial defects with topical insulin. Cornea.

[17] Bremond-Gignac D, Daruich A, Robert MP (2019). Chiambaretta,F.Recent innovations with drugs in clinical trials for neurotrophic keratitis and refractory corneal ulcers. Expert Opin Investig Drugs.

[18] Bogdan ED, Stuard WL, Titone R, Robertson DM. IGFBP-3 mediates metabolic Homeostasis during Hyperosmolar stress in the corneal epithelium. Invest Ophthalmol Vis Sci. 2021:6211. 10.1167/iovs.62.7.11 PMC8196413 34100890

[19] Sambhariya WS, Trautmann IJ (2023). Robertson,D.M.Insulin-like growth factor binding protein-3 mediates hyperosmolar stress-induced mitophagy through the mechanistic target of rapamycin. J Biol Chem.

[20] Ger T, Yang C, Ghosh S, Lai J. Biofunctionalization of nanoceria with sperminated hyaluronan enhances drug delivery performance for corneal alkali burn therapy. Chem Eng J. 2023;476:146864.

[21] Titone R, Robertson (2020). D.M.Insulin receptor preserves mitochondrial function by binding VDAC1 in insulin insensitive mucosal epithelial cells. FASEB J.

[22] Shi H, Zhou J, Wang Y, Zhu Y, Lin De, Lei L, Vakal S, Wang J, Li X. A Rapid corneal Healing Microneedle for efficient ocular. Drug Delivery Small. 2022:18e2104657. 10.1002/smll.202104657 35083856

[23] Han H, Li S, Xu M, Zhong Y, Fan W, Xu J. Zhou,T.;Ji,J.;Ye,J.;Yao,K.Polymer- and lipid-based nanocarriers for ocular drug delivery: current status and future perspectives. Adv Drug Deliv Rev.2023,196,114770. 10.1016/j.addr.2023.114770 36894134

[24] Luo L, Nguyen DD, Lai J (2020). Dually functional hollow ceria nanoparticle platform for intraocular drug delivery: a push beyond the limits of static and dynamic ocular barriers toward glaucoma therapy. Biomaterials.

[25] Jian H, Wu R, Lin T, Li Y, Lin H, Harroun SG. Huang,C.Super-Cationic Carbon Quantum dots synthesized from Spermidine as an Eye Drop Formulation for Topical Treatment of bacterial Keratitis.ACS Nano. 2017,11,6703–16. 10.1021/acsnano.7b01023 28677399

[26] Yang C, Nguyen DD. Lai,J.Poly(l-Histidine)-Mediated On-Demand therapeutic delivery of Roughened Ceria Nanocages for treatment of Chemical Eye Injury.Adv Sci.2023,10,e2302174. 10.1002/advs.202302174 PMC10502830 37430140

[27] Nguyen DD, Luo L (2023). Yang,C.;Lai,J.Highly retina-permeating and Long-Acting Resveratrol/Metformin Nanotherapeutics for Enhanced Treatment of Macular. Degeneration ACS Nano.

[28] Birk AV, Soong Y, Mills W, Singh P, Warren JD, Pardee JD (2013). Szeto,H.H.The mitochondrial-targeted compound SS-31 re-energizes ischemic mitochondria by interacting with cardiolipin. J Am Soc Nephrol.

[29] Zhao K, Luo G, Giannelli S, Szeto HH (2005). Mitochondria-targeted peptide prevents mitochondrial depolarization and apoptosis induced by tert-butyl hydroperoxide in neuronal cell lines. Biochem Pharmacol.

[30] Szeto HH. First-in-class cardiolipin-protective compound as a therapeutic agent to restore mitochondrial bioenergetics. Br J Pharmacol. 2014,1712029–50. 10.1111/bph.12461 PMC3976620 24117165

[31] Zhao T, He F, Zhao K, Yuxia L, Li H, Liu X, Cen J, Duan S;A (2023). Triple-targeted rutin-based self-assembled delivery vector for treating ischemic stroke by vascular normalization and anti-inflammation via ACE2/Ang1-7 signaling. ACS Cent Sci.

[32] Xu H, She P, Zhao Z, Ma B, Li. G.;Wang,Y.Duplex Responsive Nanoplatform with Cascade Targeting for Atherosclerosis Photoacoustic diagnosis and multichannel combination therapy. Adv Mater. 2023,35e2300439. 10.1002/adma.202300439 36828777

[33] Huang L, Gao H, Wang Z, Zhong Y (2021). Hao,L.;Du,Z.Combination Nanotherapeutics for Dry Eye Disease Treatment in a rabbit model. Int J Nanomed.

[34] Huang B, Zhang N, Qiu X, Zeng R, Wang S, Hua M, Li Q, Nan K (2024). Lin,S.Mitochondria-targeted SkQ1 nanoparticles for dry eye disease: inhibiting NLRP3 inflammasomeactivation by preventing mitochondrial. DNA Oxidation J Control Release.

[35] Yang C, Anand A, Huang C, Lai (2023). J.Unveiling the power of Gabapentin-Loaded Nanoceria with multiple therapeutic capabilities for the treatment of dry. Eye Disease ACS Nano.

[36] Lin P, Jian H, Li Y, Huang Y, Anand A, Huang C (2022). Lin,H.;Lai,J.Alleviation of dry eye syndrome with one dose of antioxidant, anti-inflammatory, and mucoadhesive lysine-carbonized nanogels. Acta Biomater.

[37] Luo L, Nguyen DD, Lai. J.Long-acting mucoadhesive thermogels for improving topical treatments of dry eye disease. Mater Sci Eng C Mater Biol Appl.2020,115,111095. 10.1016/j.msec.2020.111095 32600699

[38] Li Y, Luo L, Harroun SG, Unnikrishnan B, Chang H, Huang Y, Lai J, Huang. C.Synergistically dual-functional nano eye-drops for simultaneous anti-inflammatory and anti-oxidative treatment of dry eye disease.Nanoscale.2019,11,5580–94. 10.1039/c9nr00376b 30860532

[39] Jia F, Li L, Fang Y, Song M, Man J, Jin Q. Lei,Y.;Ji,J.Macromolecular platform with Super-cation enhanced trans-cornea infiltration for noninvasive nitric oxide delivery in ocular Therapy.ACS Nano. 2020,14,16929–38. 10.1021/acsnano.0c05977 33289535

[40] Jiang R, Li L, Li MB. Construction of degradable DNAzyme-Loaded nanocapsules for self-sufficient gene therapy of Pulmonary metastatic breast Cancer. ACS Nano. 2023,5. 10.1021/acsnano.3c09581 37925681

[41] Roy S. Zhu,D.;ParakW.J.;Feliu,N. Lysosomal Proton Buffering of Poly(ethylenimine) measured in situ by fluorescent pH-Sensor Microcapsules.ACS Nano. 2020,14,8012–23. 10.1021/acsnano.9b10219 32568521

[42] Ediriwickrema A, Zhou J, Deng Y, Saltzman WM (2014). Multi-layered nanoparticles for combination gene and drug delivery to tumors. Biomaterials.

[43] Dai CC, Yang J, Hussein WM, Zhao L, Wang X, Khalil ZG. Toth,I.;Stephenson,R.J.Polyethylenimine: an intranasal adjuvant for liposomal peptide-based subunit vaccine against Group A Streptococcus.ACS infect Dis. 2020,6,2502–12. 10.1021/acsinfecdis.0c00452 32786276

[44] Zhong D, Jiao Y, ;Zhang Y, Zhang W, Li N, Zuo Q, Wang Q. Xue,W.;Liu,Z.Effects ofthe gene carrier polyethyleneimines on structure and function of blood components.Biomaterials.2013,34.294-305. 10.1016/j.biomaterials.2012.09.060 23069714

[45] Wu D, Zhao Z, Liu H, Fu K, Ji Y, Ji W;LiY (2023). Yan,Q.;Yang,G.Escherichia Coli Nissle 1917-driven microrobots for effective tumor targeted drug delivery and tumor regression. Acta Biomater.

[46] Liang MY, Zhang MJ, Qiu W, Xiao Y, Ye MJ, Xue P, Kang YJ. Sun,ZJ.;Xu,Z.Stepwise size Shrinkage Cascade-activated Supramolecular Prodrug boosts Antitumor Immunity by Eliciting Pyroptosis. Adv Sci. 2022,9e2203353. 10.1002/advs.202203353 PMC9475545 35869614

[47] Shah S, Dhawan V, Holm R, Nagarsenker MS. Perrie,Y.Liposomes: advancements and innovation in the manufacturing process. Adv Drug Deliv Rev.2020,154–155,102–22. 10.1016/j.addr.2020.07.002 32650041

[48] Chatterjee S, Kon E. Sharma,P.;peer,D.Endosomal escape: a bottleneck for LNP-mediated therapeutics. Proc Natl Acad Sci U S A. 2024,121, e2307800120. 10.1073/pnas.2307800120 PMC10945858 38437552

[49] Akinc A, Thomas M (2005). Klibanov,AM.;Langer,R.Exploring polyethylenimine-mediated DNA transfection and the proton sponge hypothesis. J Gene Med.

[50] Sonawane ND, Szoka (2003). FC Jr.;Verkman,AS. Chloride accumulation and swelling in endosomes enhances DNA transfer by polyamine-DNA polyplexes. J Biol Chem.

[51] Jina K, .;Gea Y, Ye Z, Pana X, Yana Y, Mao Z, Ye J. Anti-oxidative and mucin-compensating dual-functional nano eye drops for synergistic treatment of dry eye disease. Appl Mater Today. 2022,27,101411.

[52] Zhang J, Gao B, Ye B, Sun Z, Qian Z;YuL, Bi Y, Ma L, Ding Y. Du,Y.;Wang,W.;Mao,Z. mitochondrial-targeted delivery of polyphenol-mediated antioxidases complexes against Pyroptosis and Inflammatory diseases. Adv Mater 2023,35e2208571. 10.1002/adma.202208571 36648306

[53] Ma B, Zhou Y, Liu R, Zhang K, Yang T, Hu C, Gao Y, Lan Q, Liu Y, Yang X, Qi (2021). H.Pigment epithelium-derived factor (PEDF) plays anti-inflammatory roles in the pathogenesis of dry eye disease. Ocul Surf.

[54] Villani E, Marelli L, Dellavalle A, Serafino M, Nucci (2020). P. latest evidences on meibomian gland dysfunction diagnosis and management. Ocul Surf.

[55] Rocha EM (2002). Cunha,DA.;Carneiro,EM.;Boschero,AC.;Saad,MJA.;Velloso,LA. Identification of insulin in the tear film and insulin receptor and IGF-1 receptor on the human ocular surface. Invest Ophthalmol Vis Sci.

[56] Naeser (1997). Insulin receptors in human ocular tissues.Immunohistochemical demonstration in normal and diabetic eyes. Upsala J Med Sci.

[57] Cunha DA, Carneiro EM, Alves,Mde C, Jorge AG, Sousa SM, Boschero AC, Velloso LA (2005). Rocha,E.M.Insulin secretion by rat lacrimal glands: effects of systemic and local variables. Am J Physiol Endocrinol Metab.

[58] Friend J, Snip RC (1980). Kiorpes,TC.;Thoft,RA. Insulin sensitivity and sorbitol production of the normal rabbit corneal epithelium in vitro. Invest Ophthalmol Vis Sci.

[59] DeBerardinis RJ, Mancuso A, Daikhin E, Nissim I, Yudkoff M, Wehrli S. Thompson,C.B.Beyond aerobic glycolysis: Transformed cells can engage in glutamine metabolism that exceeds the requirement for protein and nucleotide synthesis.Proc.Natl.Acad.Sci.U.S.A. 2007,104,19345–19350. 10.1073/pnas.0709747104 PMC2148292 18032601

[60] SriRamaratnam,R.;WELSCH YANGWS,M, Shimada E. K.;Skouta,k.;Viswanathan,V.S.;Cheah,J.H.;Clemons,P.A.;Shamji,A.F.;Clish,C.B.;Brown,L.M.;Girotti,A.W.;Cornish,V.W.;Schreiber,S.L.;Stockwell,B.R.Regulation of ferroptotic cancer cell death by GPX4.Cell. 2014,156,317–331. 10.1016/j.cell.2013.12.010 PMC4076414 24439385

[61] Wu K, Yan M;LiuT, Wang Z, Duan Y, Xia Y, Ji G, Shen YWL, Li L, Zheng P, Dong B (2023). Xu,D.Creatine kinase B suppresses ferroptosis by phosphorylating GPX4 through a moonlighting function. Nat Cell Biol.

[62] Xiong X, Jiang H, Liao Y, Du Y, Zhang Y, Wang Z. Zheng,M.;Du,Z.Liposome-trimethyl chitosan nanoparticles codeliver insulin and siVEGF to treat corneal alkali burns by inhibiting ferroptosis. Bioeng Transl Med. 2023,8e10499. 10.1002/btm2.10499 PMC10013822 36925675

[63] Schapira (2006). AHV.Mitochondrial disease. Lancet.

[64] Beckmann L, Cai Z, Margolis M, Fang R, Djalilian. A.;Zhang,HF.Recent advances in optical coherence tomography for anterior segment imaging in small animals and their clinical implications.Ocul surf. 2022,26,222–33. 10.1016/j.jtos.2022.08.011 PMC10040227 36195237

[65] Kojima T, Dogru M, Kawashima M, Nakamura. S.;Tsubota,k.Advances in the diagnosis and treatment of dry eye. Prog Retin Eye Res. 2020,29100842. 10.1016/j.preteyeres.2020.100842 32004729

[66] Li S, Lu Z, Huang Y, WangY.;Jin Q. Shentu,X.;Ye,J.;Ji,J.;Yao,K.;Han,H.Anti-Oxidative and anti-inflammatory micelles: Break the Dry Eye Vicious Cycle. Adv Sci. 2022,9e2200435. 10.1002/advs.202200435 PMC9189644 35435328

[67] Tsubota K, Shimazaki J, Watanabe H, Dogru M, Yamada M, Kinoshita S, Kim HM, Tchah HW, Hyon JY, Yoon KC, Seo KY, Sun X (2017). S.New perspectives on Dry Eye Definition and diagnosis: a Consensus Report by the Asia Dry Eye Society. Ocul Surf.

[68] Dinarello CA. Immunological and inflammatory functions of the interleukin-1 family. Annu Rev Immunol. 2009,27,519 – 50. 10.1146/annurev.immunol.021908.132612 19302047

