# Supplementary material for: Comprehensive dry eye therapy: overcoming ocular surface barrier and combating inflammation, oxidation, and mitochondrial damage
Source: J Nanobiotechnology. 2024 May 9;22:233. doi: 10.1186/s12951-024-02503-7 (PMC11080212; doi:10.1186/s12951-024-02503-7)
Supplement: Supplementary file 1 — Supplementary Material 1 [file 12951_2024_2503_MOESM1_ESM.docx]

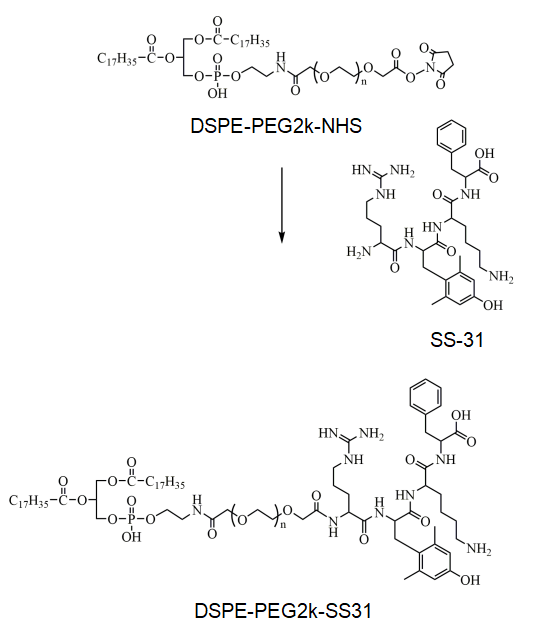


**Figure S1.** Illustration of the synthesis of DSPE-PEG2k-SS31.


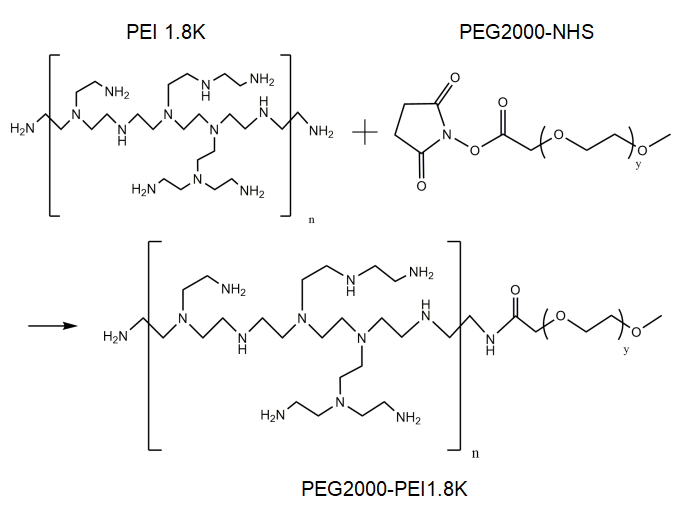


**Figure S2.** Illustration of the synthesis of PEG2000-PEI.


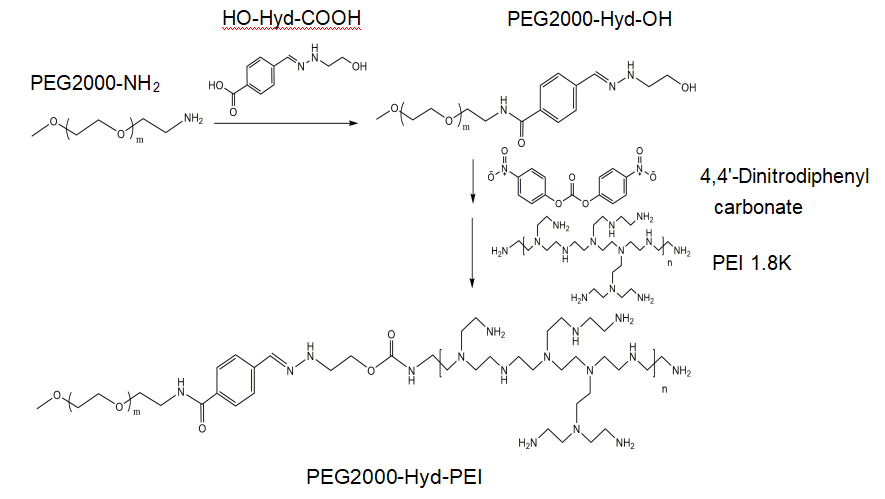


**Figure S3.** Illustration of the synthesis of PEG2000-Hyd-PEI.


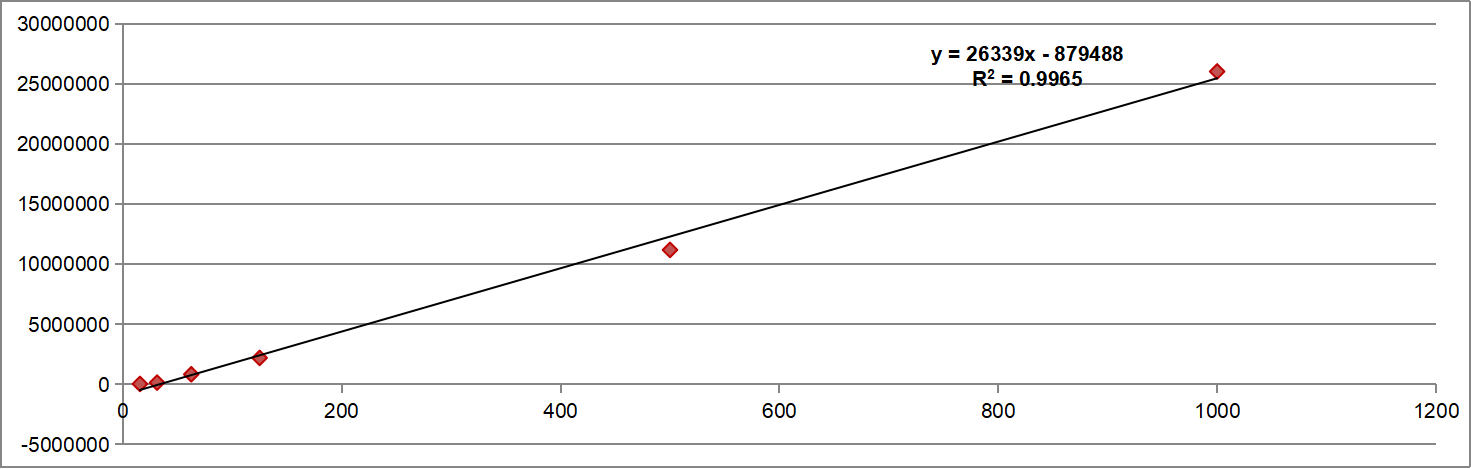


**Figure S4.** Calibration Curve of Insulin by Ultra-High Performance Liquid Chromatography (UHPLC).


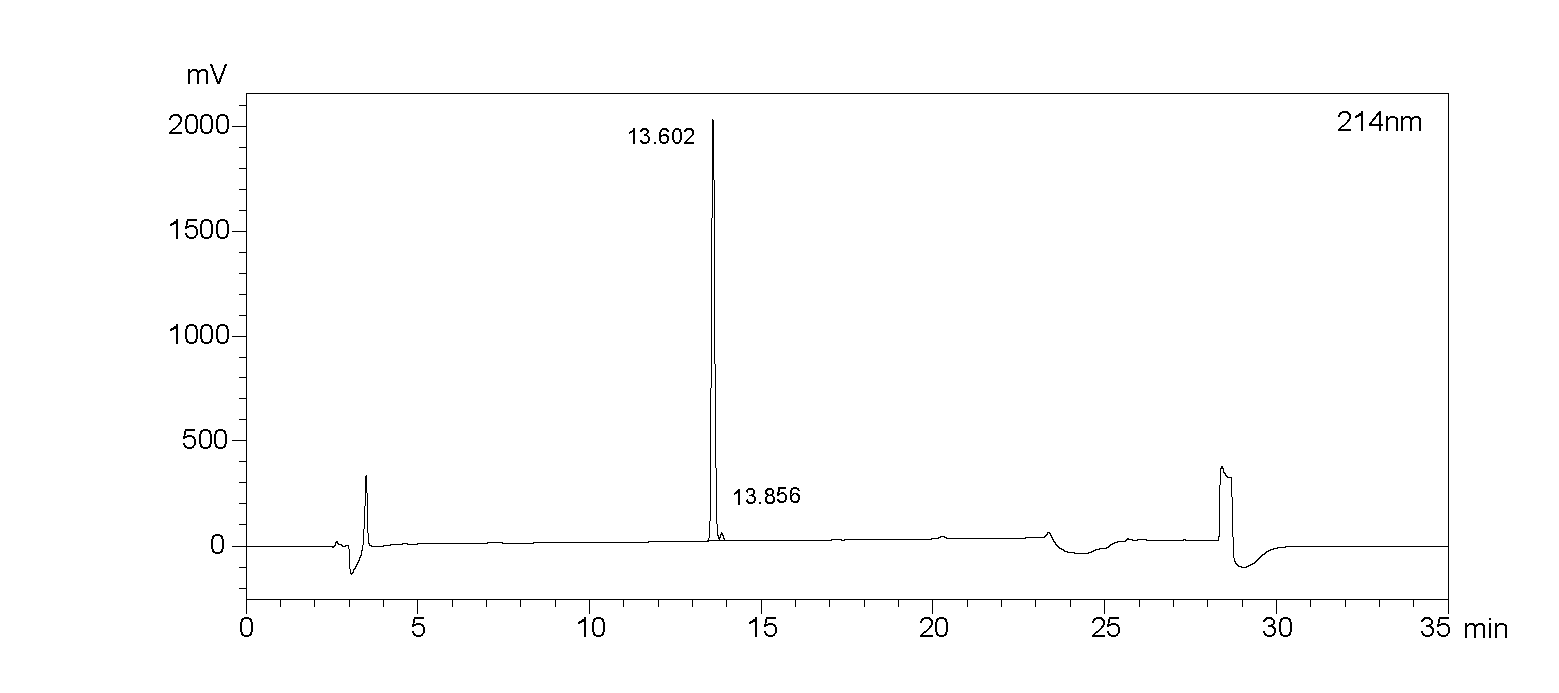

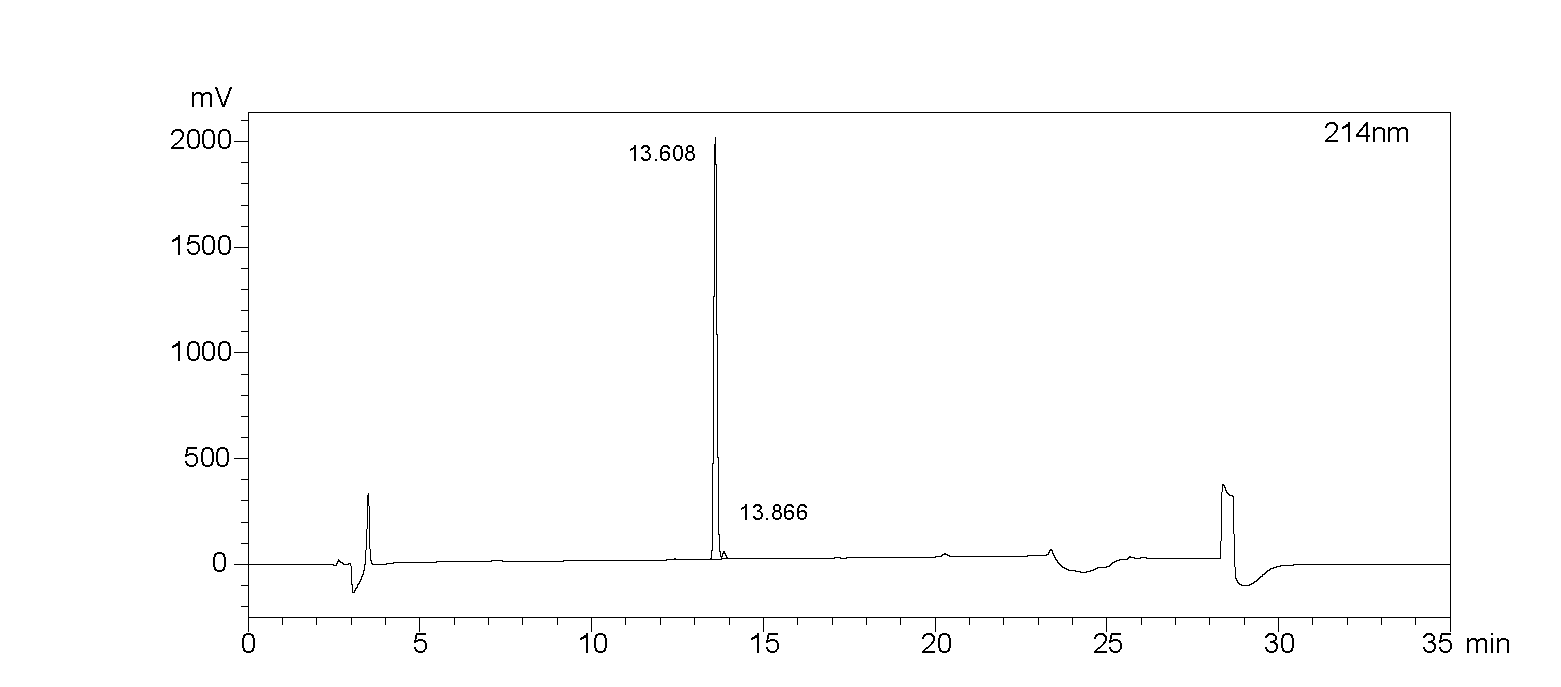

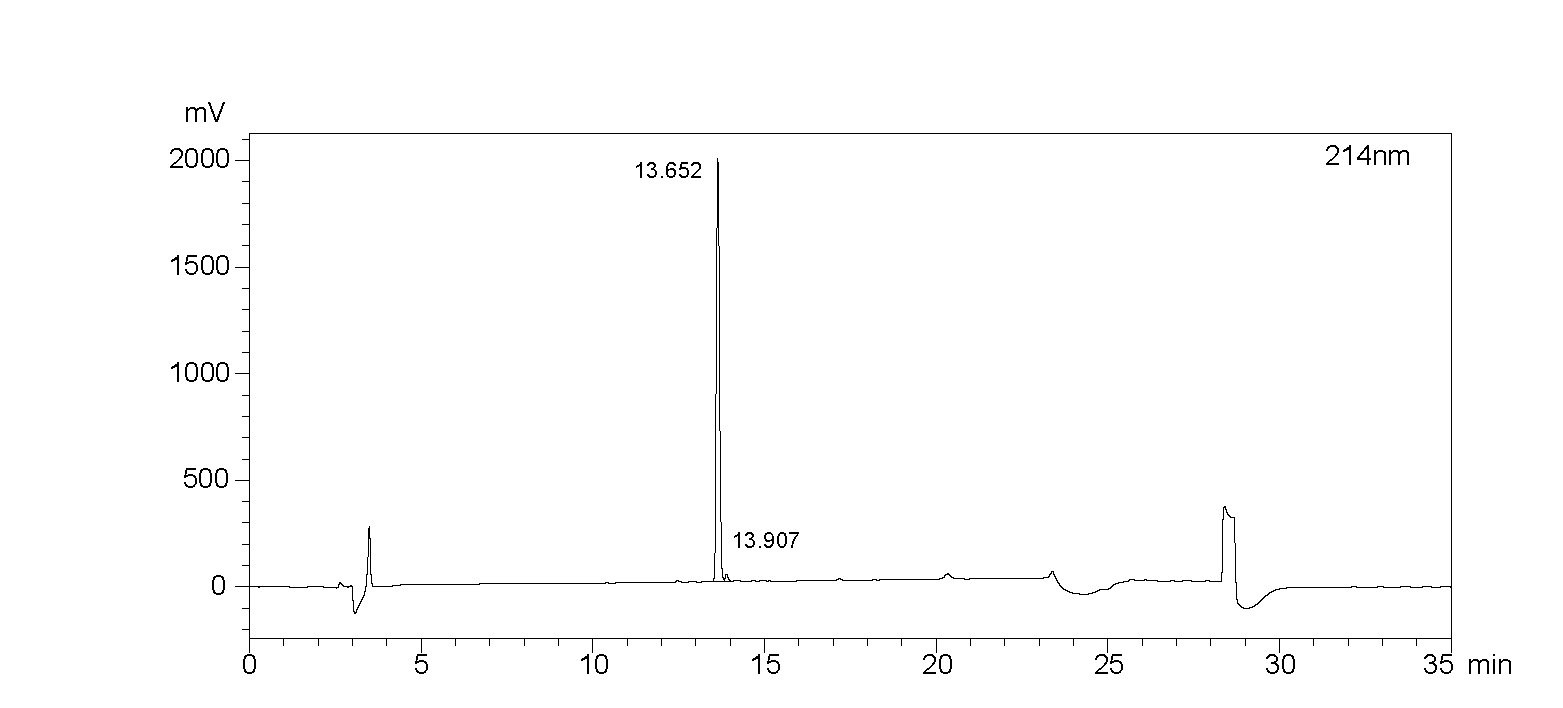


b.

a.

c.

**Figure S5.** UHPLC chromatogram of PHP-DPS@INS NPs loaded INS (n=3).(a).The retention time of wave peak 1 is 13.602 minutes, with an area of 12,899,615 and a height of 2,008,843. The retention time of wave peak 2 is 13.856 minutes, with an area of 204,313 and a height of 35,942.(b). The retention time of wave peak 1 is 13.608 minutes, with an area of 12,760,395 and a height of 1,992,563. The retention time of wave peak 2 is 13.866 minutes, with an area of 199,603 and a height of 34,672.(c).The retention time of wave peak 1 is 13.652 minutes, with an area of 12,720,504 and a height of 1,981,525. The retention time of wave peak 2 is 13.907 minutes, with an area of 184,253 and a height of 32,709.


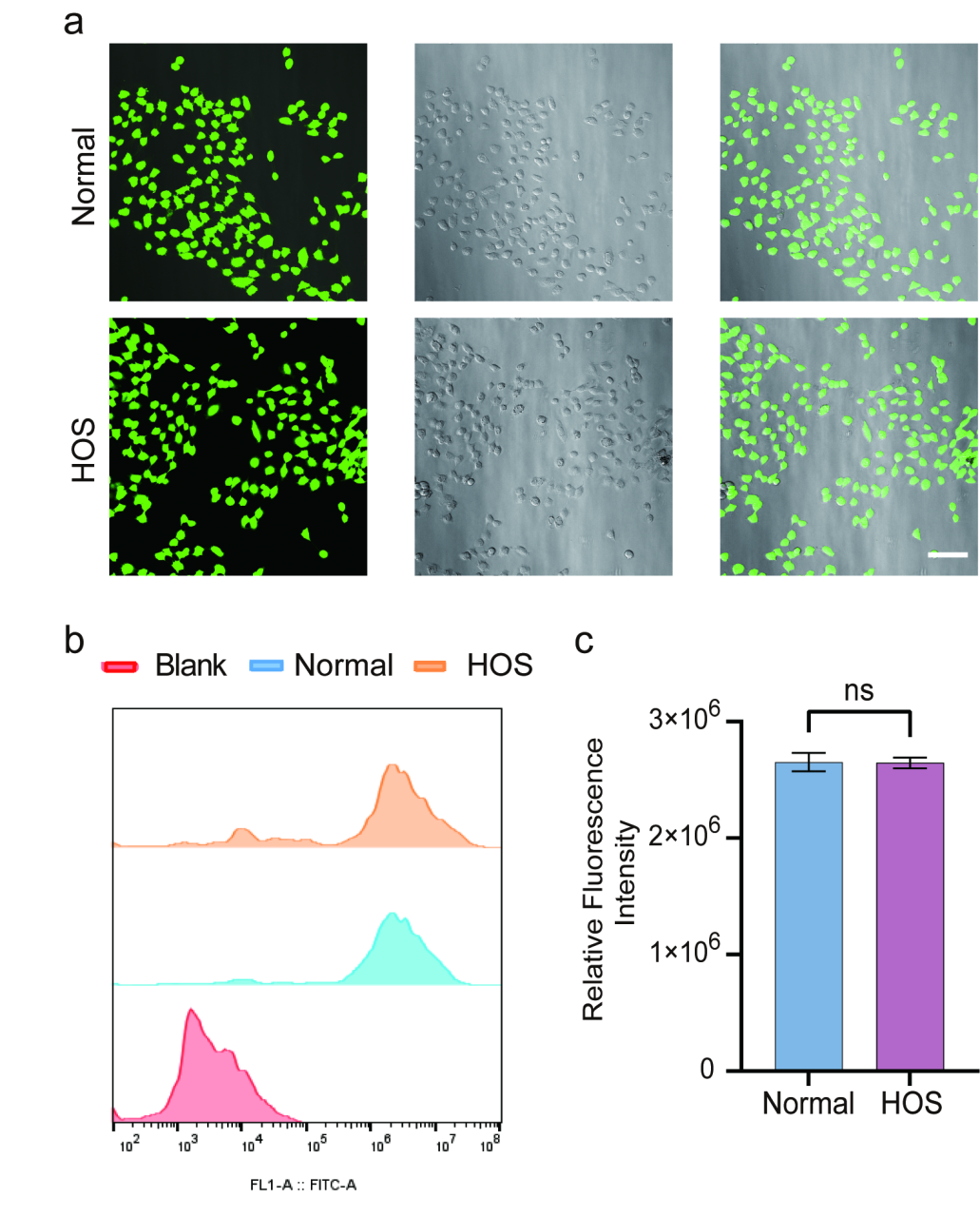


**Figure S6.**(a)The bright field image (scale: 50 μm), (b) flow cytometry and (c)quantification of intracellular pH fluorescence between normal HCECs and HOS-cultured HCECs using BCECF-AM staining (n=3).


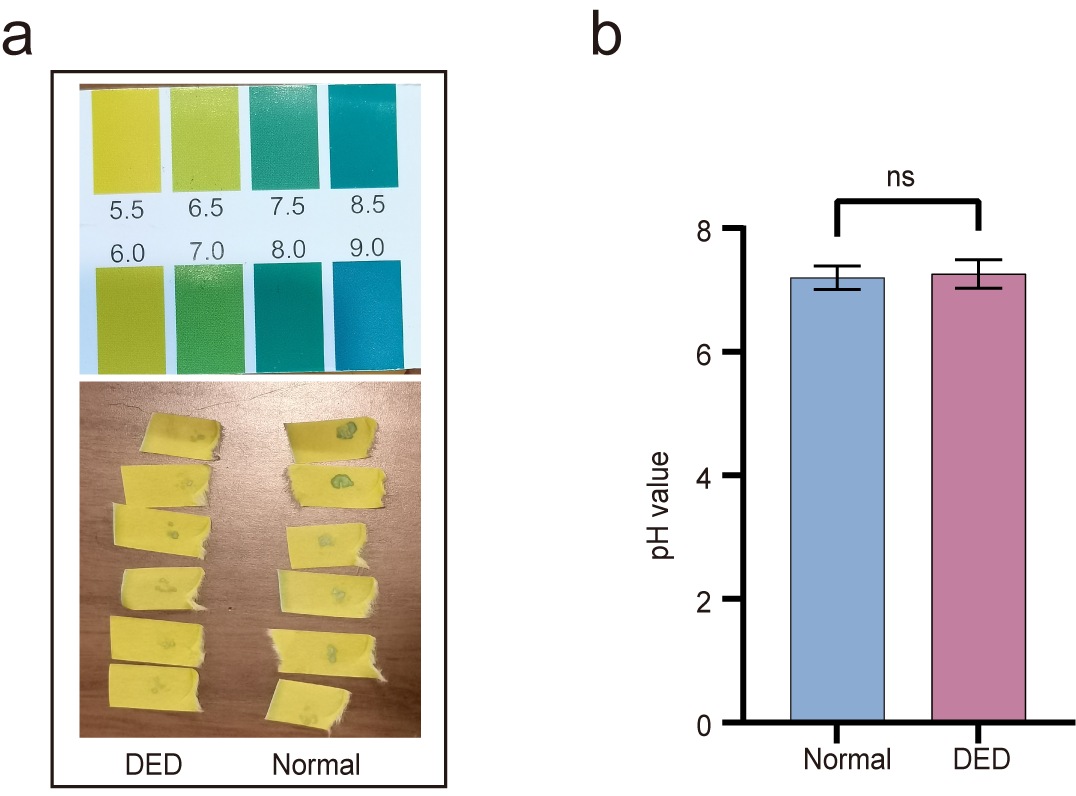


**Figure S7.**(a)Measurement of ocular surface pH values and (b)comparison between normal and DED mice using pH paper (n=6).

**Table S1**. Encapsulation efficiency (EE) and drug-loading capacity (DLC) of PHP-DPS@INS NPs by UHPLC (n=3).

|  | EE(%) | DL (%) |
| --- | --- | --- |
| 1 | 49.31 | 9.86 |
| 2 | 48.78 | 9.76 |
| 3 | 48.63 | 9.73 |

EE:Encapsulation Efficiency;DL:Drug Loading.

**Table S2**.The release rate of INS from PHP-DPS@INS NPs by HPLC at 37℃for 12 h under different pH conditions.

| Time  (min) | PH=4.8 | | |  | PH=5.8 | | |  | PH=7.4 | | |
| --- | --- | --- | --- | --- | --- | --- | --- | --- | --- | --- | --- |
|  | RR 1  (%) | RR 2  (%) | RR 3  (%) |  | RR 1  (%) | RR 2  (%) | RR 3  (%) |  | RR 1  (%) | RR 2  (%) | RR 3  (%) |
| 0.5 | 2.21 | 2.01 | 2.05 |  | 1.69 | 1.56 | 1.64 |  | 1.63 | 1.54 | 1.48 |
| 1 | 11.79 | 10.81 | 10.65 |  | 6.62 | 6.55 | 6.66 |  | 3.21 | 3.22 | 3.22 |
| 2 | 15.21 | 13.90 | 13.90 |  | 9.78 | 10.21 | 10.24 |  | 4.29 | 4.58 | 4.51 |
| 4 | 26.59 | 25.53 | 25.50 |  | 18.21 | 18.94 | 19.39 |  | 10.77 | 10.93 | 11.07 |
| 6 | 46.62 | 45.13 | 45.65 |  | 36.05 | 38.07 | 38.42 |  | 18.30 | 17.73 | 17.79 |
| 8 | 63.14 | 62.68 | 63.15 |  | 48.10 | 51.02 | 51.83 |  | 29.85 | 28.28 | 28.50 |
| 10 | 67.25 | 67.94 | 68.47 |  | 55.47 | 59.46 | 59.89 |  | 41.49 | 39.46 | 40.14 |
| 12 | 70.29 | 72.20 | 72.44 |  | 61.06 | 66.62 | 67.05 |  | 53.08 | 50.77 | 51.41 |

RR：Release rate.


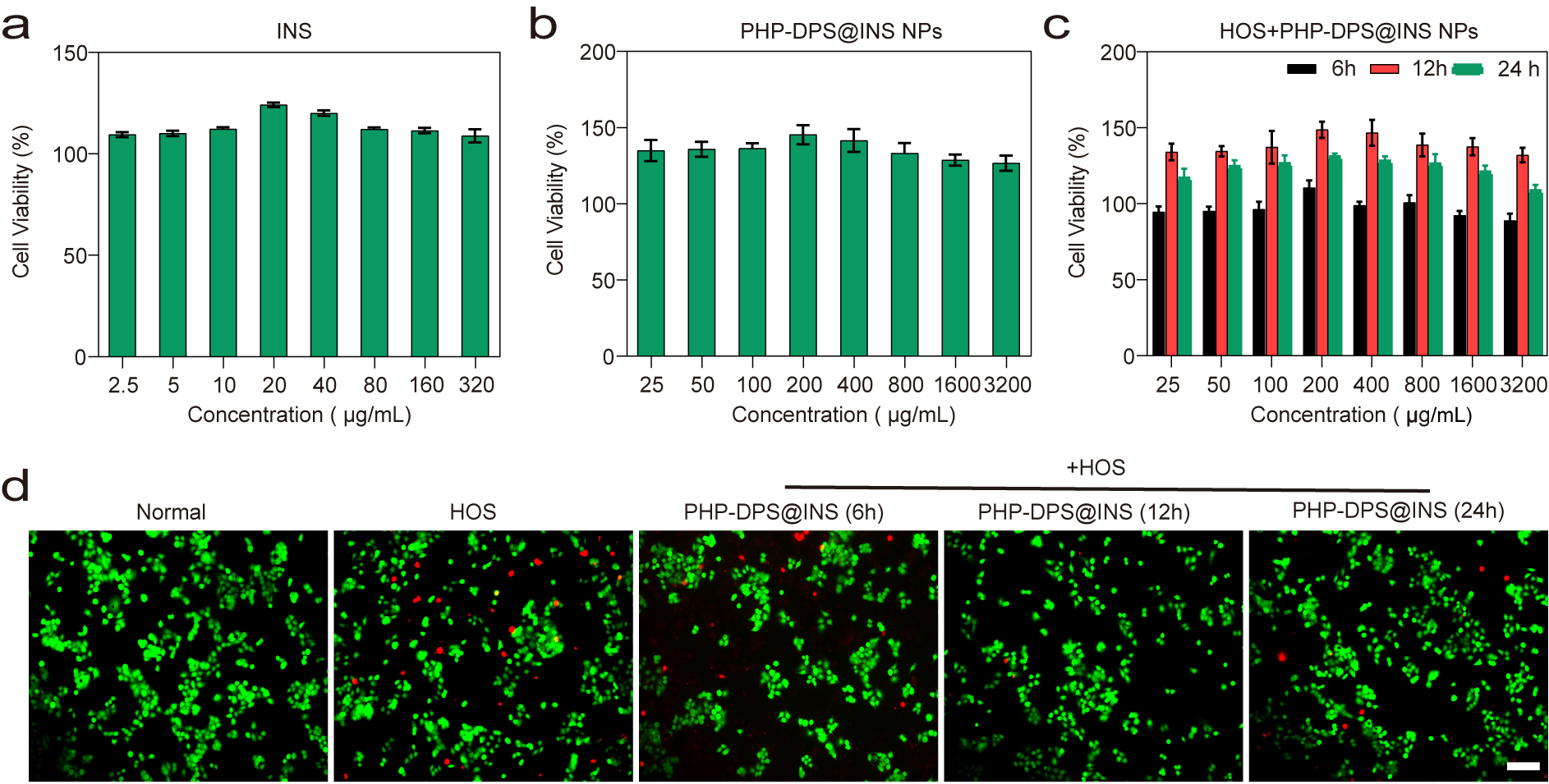


**Figure S8.**The cytotoxicity testing of (a)INS and (b)PHP-DPS@INS NPs in normal HCECs for 24 h by CCK-8 method. (c)The cell viability of the HOS-cultured HCECs incubated with PHP-DPS@INS NPs for r 6h,12 h and 24 h by CCK-8 method.(d)Fluorescence images of Calcein AM/PI staining in the Normal,HOS-cultured HCECs ,HOS-cultured HCEC treated with PHP-DPS@INS NPs for 6 h,12h and 24h.Values are presented as mean±SD(n=3；scale bars:100μm).HOS:HCECs cultured in hyperosmolar stress(450 mOsM) for 12 hours.


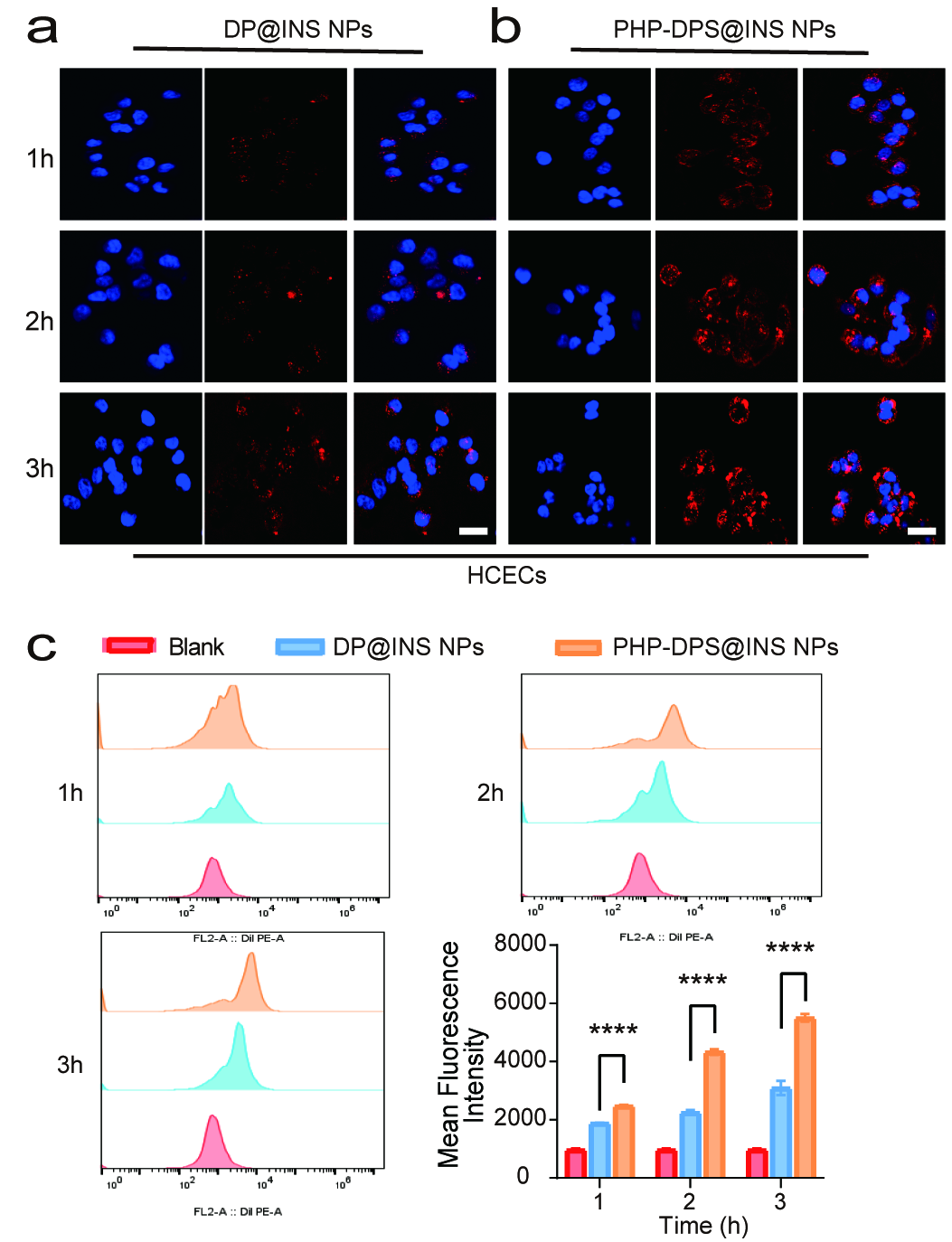


**Figure S9.**(a) Fluorescence images of normal HCECs incubated with DP@INS NPs and (b) PHP-DPS@INS NPs at different treatment times (Blue: DAPI; Red: DiI-labeled nanoparticles; scale bars: 50 μm). (c) Flow cytometric curves and quantitative results of uptake of DiI-DP@INS NPs and DiI-PHP-DPS@INS NPs at 1, 2, and 3h in normal HCECs.

**Table S3.** Significantly Altered Metabolites in Normal group and HOS group

| Metabolite | VIP | Fold Change | *P* Value | Mean in Normal | Mean in HOS |
| --- | --- | --- | --- | --- | --- |
|  |  |  |  |  |  |
| Acetylcholine | 1.99 | 2.19 | 0.00 | 0.00 | 0.00 |
| L-Phenylalanine | 1.96 | 2.62 | 0.00 | 0.04 | 0.10 |
| L-Norleucine | 1.94 | 2.62 | 0.00 | 0.01 | 0.04 |
| L-Leucine | 2.00 | 2.60 | 0.01 | 0.03 | 0.09 |
| Kynurenine | 1.59 | 6.25 | 0.01 | 0.00 | 0.00 |
| L-Tryptophan | 1.87 | 2.30 | 0.00 | 0.01 | 0.03 |
| Glycylphenylalanine | 1.54 | 1.41 | 0.03 | 0.00 | 0.00 |
| Pantothenic acid | 1.72 | 1.63 | 0.01 | 0.01 | 0.01 |
| Norvaline | 1.54 | 4.09 | 0.01 | 0.00 | 0.00 |
| Epinephrine | 1.95 | 1.99 | 0.00 | 0.05 | 0.09 |
| L-Valine | 1.84 | 2.58 | 0.00 | 0.07 | 0.19 |
| L-Proline | 1.75 | 2.60 | 0.01 | 0.04 | 0.11 |
| L-Tyrosine | 1.94 | 2.82 | 0.00 | 0.04 | 0.11 |
| Hydroxyproline | 1.91 | 2.28 | 0.00 | 0.00 | 0.00 |
| Taurine | 1.80 | 1.77 | 0.03 | 0.01 | 0.01 |
| L-Threonine | 1.76 | 3.06 | 0.05 | 0.01 | 0.03 |
| L-Homoserine | 1.85 | 1.92 | 0.02 | 0.00 | 0.00 |
| L-Histidine | 1.55 | 1.72 | 0.05 | 0.01 | 0.02 |
| L-Glycine | 1.69 | 2.02 | 0.05 | 0.06 | 0.12 |
| beta-Alanine | 1.85 | 2.01 | 0.00 | 0.03 | 0.05 |
| L-Glutamine | 1.85 | 2.14 | 0.01 | 0.01 | 0.02 |
| Glycerophosphocholine | 1.67 | 1.34 | 0.05 | 0.03 | 0.04 |
| 4-Aminobutyric acid | 1.74 | 2.10 | 0.01 | 0.00 | 0.01 |
| L-Serine | 1.89 | 2.13 | 0.01 | 0.01 | 0.03 |
| N-Acetyl-Neuraminic Acid | 1.87 | 1.57 | 0.01 | 0.56 | 0.88 |
| Cysteine-S-sulfate | 1.72 | 2.17 | 0.04 | 0.00 | 0.00 |
| NAD+ | 1.99 | 2.88 | 0.01 | 0.05 | 0.14 |
| gamma-Glutamylalanine | 1.88 | 3.00 | 0.03 | 0.00 | 0.00 |
| L-Glutamic acid | 2.04 | 2.38 | 0.00 | 1.17 | 2.79 |
| L-Carnosine | 1.99 | 2.41 | 0.00 | 0.00 | 0.00 |
| L-Aspartic acid | 1.95 | 1.71 | 0.01 | 0.47 | 0.81 |
| N-Acetylglucosaminylasparagine | 1.98 | 1.72 | 0.00 | 0.01 | 0.01 |
| L-2-Aminoadipic Acid | 2.03 | 2.49 | 0.00 | 0.00 | 0.00 |
| Guanidinosuccinic acid | 1.88 | 2.40 | 0.00 | 0.00 | 0.00 |
| S-Adenosylmethionine | 1.81 | 2.61 | 0.02 | 0.09 | 0.22 |
| Inosinic acid | 1.92 | 1.79 | 0.00 | 0.16 | 0.29 |
| O-Phosphoethanolamine | 1.87 | 2.14 | 0.02 | 0.00 | 0.01 |
| Cytidine-5'-monophosphate | 1.89 | 1.81 | 0.01 | 0.02 | 0.04 |
| Thiamine pyrophosphate | 1.76 | 1.58 | 0.03 | 0.01 | 0.01 |
| L-Argininosuccinic Acid | 1.99 | 2.56 | 0.00 | 0.00 | 0.01 |
| L-Homoarginine | 1.80 | 1.93 | 0.01 | 0.00 | 0.00 |
| L-Arginine | 1.89 | 2.14 | 0.02 | 0.10 | 0.21 |
| Nicotinamide adenine dinucleotide phosphate | 1.98 | 1.85 | 0.00 | 0.06 | 0.11 |
| N-Formyl-L-methionine | 1.61 | 1.77 | 0.01 | 0.00 | 0.00 |
| Hydroxyphenyllactic acid | 1.98 | 2.02 | 0.00 | 0.00 | 0.00 |
| Orotic acid | 1.92 | 2.06 | 0.00 | 0.00 | 0.00 |
| L-Arabitol | 1.67 | 1.58 | 0.04 | 0.00 | 0.00 |
| N-Acetyl-L-alanine | 2.03 | 2.00 | 0.00 | 0.00 | 0.00 |
| 3-Hydroxyisobutyric acid | 1.79 | 2.31 | 0.02 | 0.00 | 0.00 |
| L-Threonic acid | 2.00 | 2457.20 | 0.02 | 0.00 | 0.00 |
| Inositol | 1.75 | 2.73 | 0.04 | 0.26 | 0.72 |
| 2-Methylglutaric acid | 0.95 | 2.22 | 0.03 | 0.00 | 0.00 |
| Itaconic acid | 1.94 | 2.03 | 0.00 | 0.00 | 0.00 |
| N-Acetyl-L-glutamic acid | 1.60 | 1.54 | 0.01 | 0.00 | 0.00 |
| Succinic acid | 1.91 | 1.90 | 0.00 | 0.01 | 0.02 |
| Thymidine-5'-diphosphate | 1.57 | 1.53 | 0.03 | 0.00 | 0.01 |
| L-Malic acid | 1.90 | 2.71 | 0.03 | 0.02 | 0.04 |
| UDP-D-glucose | 1.76 | 1.70 | 0.02 | 0.04 | 0.07 |
| Fumaric acid | 1.87 | 2.36 | 0.01 | 0.02 | 0.05 |
| N-carbamoyl-L-aspartate | 1.97 | 2.38 | 0.02 | 0.00 | 0.00 |
| Uridine-5'-diphosphate | 1.83 | 1.83 | 0.01 | 0.02 | 0.04 |
| Glutathione Disulfide | 1.70 | 1.49 | 0.03 | 0.11 | 0.16 |

Detected by targeted Metabolomics and UPLC-QQQ-MS analysis.HOS:HCECs cultured in hyp-erosmolar stress(450 mOsM) for 12 hours.

*
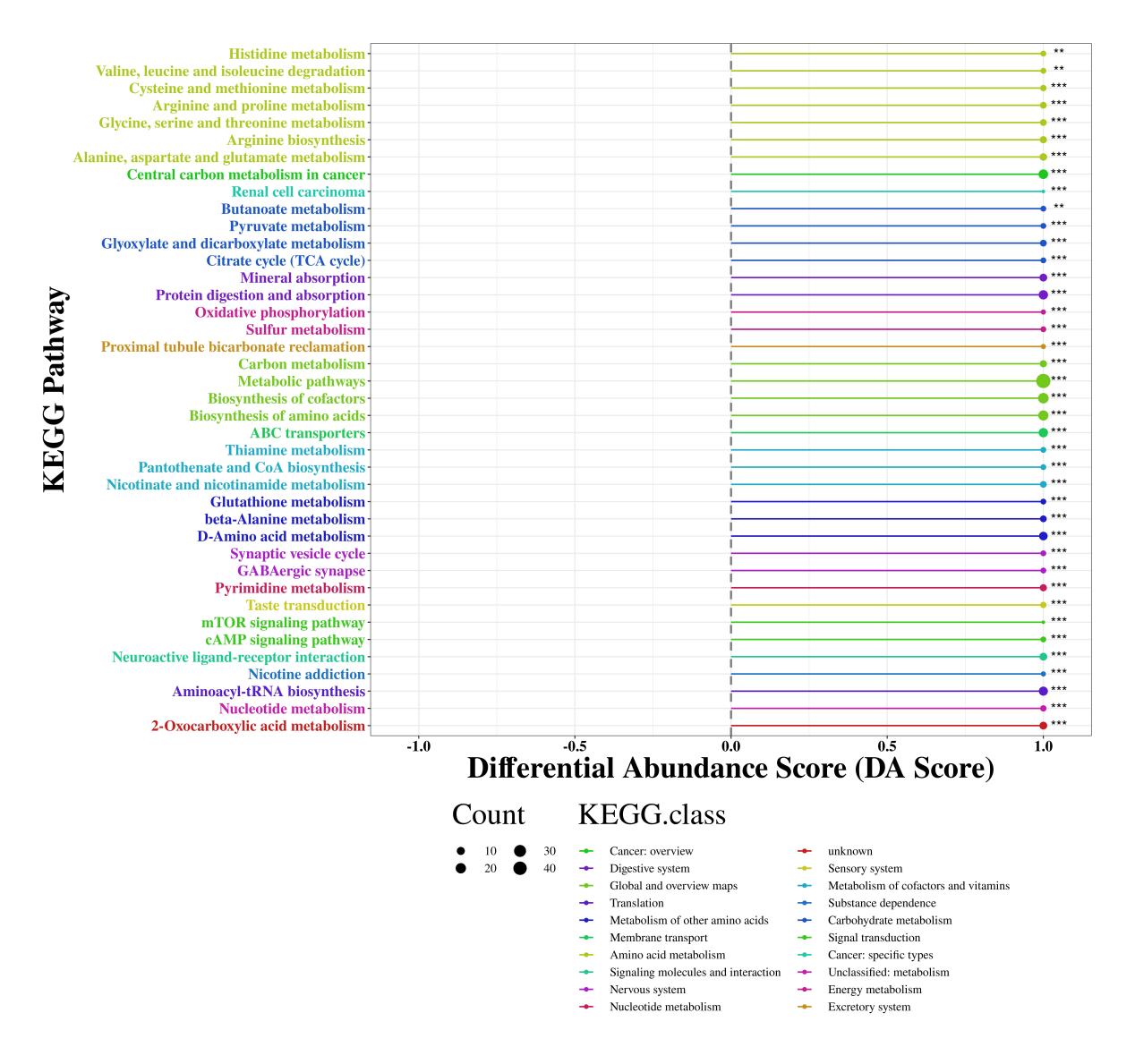
*

**Figure S10.**Differential abundance score of KEGG pathway for Normal group and HOS gro-up.Detected by targeted Metabolomics and UPLC-QQQ-MS analysis.score 1.0：the expression of metabolites in this pathway is up-regulated;score -1.0:the expression of differential metabolites in this pathway is down-regulated；the length of the line：the absolute value ofDA Score；the size of the dot：the number of differential metabolites noted in this pathway.HOS:HCECs cultured in hyperosmolar stress(450 mOsM) for 12 hours.

**Table S4.** Pathway Analysis for Normal group and HOS group（According to the Impact value from big to small）

| Pathway | Total | Hits | *P* Value | Impact |
| --- | --- | --- | --- | --- |
| Alanine, aspartate and glutamate metabolism | 24 | 7 | 0 | 0.65 |
| Glycine, serine and threonine metabolism | 48 | 6 | 0 | 0.49 |
| Arginine and proline metabolism | 77 | 10 | 0 | 0.36 |
| beta-Alanine metabolism | 28 | 5 | 0 | 0.28 |
| Pantothenate and CoA biosynthesis | 27 | 4 | 0 | 0.25 |
| Aminoacyl-tRNA biosynthesis | 75 | 14 | 0 | 0.17 |
| Cysteine and methionine metabolism | 56 | 5 | 0.01 | 0.14 |
| Histidine metabolism | 44 | 4 | 0.01 | 0.14 |
| D-Glutamine and D-glutamate metabolism | 11 | 2 | 0.02 | 0.14 |
| Phenylalanine metabolism | 45 | 4 | 0.02 | 0.12 |
| Pyrimidine metabolism | 60 | 7 | 0 | 0.09 |
| Propanoate metabolism | 35 | 3 | 0.04 | 0.09 |
| Citrate cycle (TCA cycle) | 20 | 3 | 0.01 | 0.08 |
| Valine, leucine and isoleucine degradation | 40 | 4 | 0.01 | 0.08 |
| Lysine biosynthesis | 32 | 3 | 0.03 | 0.07 |
| Tyrosine metabolism | 76 | 5 | 0.02 | 0.05 |
| Butanoate metabolism | 40 | 4 | 0.01 | 0.04 |
| Thiamine metabolism | 24 | 3 | 0.01 | 0.03 |
| Valine, leucine and isoleucine biosynthesis | 27 | 3 | 0.02 | 0.03 |
| Glutathione metabolism | 38 | 4 | 0.01 | 0.02 |
| Phenylalanine, tyrosine and tryptophan biosynthesis | 27 | 3 | 0.02 | 0.01 |
| Nitrogen metabolism | 39 | 9 | 0 | 0 |
| Cyanoamino acid metabolism | 16 | 3 | 0 | 0 |
| Nicotinate and nicotinamide metabolism | 44 | 4 | 0.01 | 0 |

Detected by targeted Metabolomics and UPLC-QQQ-MS analysis.Impact:the influence factors obtained from the topological analysis.HOS:HCECs cultured in hyperosmolar stress(450 mOsM) for 12 hours.

**Table S5.** Significantly Altered Metabolites for HOS+INS group and HOS group

| Metabolite | VIP | Fold Change | *P* Value | Mean in HOS+INS | Mean in HOS |
| --- | --- | --- | --- | --- | --- |
|  |  |  |  |  |  |
| Erucamide | 1.48 | 0.34 | 0.00 | 0.14 | 0.05 |
| Calcifediol | 1.18 | 2.73 | 0.04 | 0.22 | 0.59 |
| Nicotinamide | 1.43 | 2.70 | 0.04 | 0.11 | 0.29 |
| 2'-Deoxyadenosine | 1.47 | 0.29 | 0.00 | 0.00 | 0.00 |
| Acetylcholine | 1.47 | 10.21 | 0.00 | 0.00 | 0.00 |
| Deoxyinosine | 0.98 | 0.31 | 0.02 | 0.00 | 0.00 |
| L-Phenylalanine | 1.50 | 2.92 | 0.00 | 0.03 | 0.10 |
| L-Norleucine | 1.57 | 3.36 | 0.00 | 0.01 | 0.04 |
| Trimethylamine | 1.32 | 2.30 | 0.02 | 0.21 | 0.49 |
| L-Leucine | 1.55 | 3.87 | 0.00 | 0.02 | 0.09 |
| Kynurenine | 1.11 | 3.20 | 0.03 | 0.00 | 0.00 |
| 1-Methyladenosine | 1.30 | 2.04 | 0.02 | 0.00 | 0.00 |
| L-Tryptophan | 1.36 | 2.43 | 0.01 | 0.01 | 0.03 |
| Pyridoxamine | 1.36 | 6.61 | 0.01 | 0.00 | 0.00 |
| Pantothenic acid | 1.26 | 1.91 | 0.02 | 0.01 | 0.01 |
| Glycyl-L-Leucine | 1.19 | 1.55 | 0.03 | 0.00 | 0.00 |
| 3,4-Dihydroxymandelic acid | 1.35 | 0.65 | 0.03 | 0.01 | 0.00 |
| L-Valine | 1.48 | 3.12 | 0.00 | 0.06 | 0.19 |
| Ethanolamine | 1.69 | 70925.35 | 0.04 | 0.00 | 0.00 |
| L-Tyrosine | 1.54 | 3.73 | 0.00 | 0.03 | 0.11 |
| N-Acetylglutamine | 1.44 | 3.87 | 0.04 | 0.00 | 0.00 |
| Taurine | 1.46 | 2.63 | 0.01 | 0.01 | 0.01 |
| L-Threonine | 1.44 | 3.66 | 0.03 | 0.01 | 0.03 |
| L-Homoserine | 1.55 | 2.52 | 0.00 | 0.00 | 0.00 |
| gamma-Glutamylmethionine | 1.56 | 50.10 | 0.04 | 0.00 | 0.00 |
| L-Histidine | 1.21 | 1.77 | 0.04 | 0.01 | 0.02 |
| L-Glycine | 1.51 | 2.54 | 0.01 | 0.05 | 0.12 |
| beta-Alanine | 1.25 | 2.06 | 0.02 | 0.03 | 0.05 |
| L-Glutamine | 1.59 | 3.61 | 0.00 | 0.01 | 0.02 |
| Glycerophosphocholine | 1.64 | 4.05 | 0.00 | 0.01 | 0.04 |
| L-Asparagine | 1.41 | 1.81 | 0.03 | 0.01 | 0.01 |
| gamma-L-Glutamyl-L-valine | 1.65 | 3.01 | 0.00 | 0.00 | 0.00 |
| L-Serine | 1.62 | 2.99 | 0.00 | 0.01 | 0.03 |
| N-Acetyl-Neuraminic Acid | 1.66 | 8.38 | 0.00 | 0.11 | 0.88 |
| 5-Aminovaleric acid | 1.50 | 3.14 | 0.00 | 0.00 | 0.00 |
| Cysteine-S-sulfate | 1.29 | 15.17 | 0.00 | 0.00 | 0.00 |
| NAD+ | 1.62 | 6.08 | 0.01 | 0.02 | 0.14 |
| gamma-Glutamylalanine | 1.63 | 15.52 | 0.01 | 0.00 | 0.00 |
| D-Glucosamine-6-sulfate | 1.36 | 8.61 | 0.02 | 0.00 | 0.00 |
| L-Glutamic acid | 1.63 | 4.07 | 0.00 | 0.68 | 2.79 |
| L-Carnosine | 1.66 | 4.27 | 0.00 | 0.00 | 0.00 |
| L-Aspartic acid | 1.66 | 7.25 | 0.00 | 0.11 | 0.81 |
| N-Acetylglucosaminylasparagine | 1.65 | 4.73 | 0.00 | 0.00 | 0.01 |
| N,N-Dimethylarginine | 1.66 | 4.92 | 0.01 | 0.00 | 0.00 |
| Guanidinosuccinic acid | 1.37 | 2.53 | 0.00 | 0.00 | 0.00 |
| S-Adenosylmethionine | 1.63 | 6.59 | 0.02 | 0.03 | 0.22 |
| Inosinic acid | 1.68 | 3.99 | 0.00 | 0.07 | 0.29 |
| 5-Methyltetrahydrofolic acid | 1.67 | 7.36 | 0.01 | 0.00 | 0.00 |
| Citicoline | 1.48 | 2.27 | 0.03 | 0.01 | 0.02 |
| L-Saccharopine | 1.47 | 2.04 | 0.01 | 0.00 | 0.00 |
| O-Phosphoethanolamine | 1.57 | 7.10 | 0.00 | 0.00 | 0.01 |
| Cytidine-5'-monophosphate | 1.62 | 6.50 | 0.00 | 0.01 | 0.04 |
| Ciliatine | 1.35 | 9.20 | 0.03 | 0.00 | 0.00 |
| Thiamine pyrophosphate | 1.63 | 4.91 | 0.00 | 0.00 | 0.01 |
| Thiamine monophosphate | 1.57 | 5.37 | 0.01 | 0.00 | 0.00 |
| Phosphorylcholine Chloride | 1.53 | 2.25 | 0.00 | 0.70 | 1.57 |
| O-Phospho-L-Serine | 1.56 | 2.40 | 0.00 | 0.10 | 0.24 |
| L-Argininosuccinic Acid | 1.48 | 2.29 | 0.00 | 0.00 | 0.01 |
| L-Homoarginine | 1.59 | 2.79 | 0.00 | 0.00 | 0.00 |
| N6,N6,N6-Trimethyl-L-lysine | 1.67 | 4.85 | 0.00 | 0.00 | 0.00 |
| L-Arginine | 1.58 | 4.73 | 0.00 | 0.04 | 0.21 |
| Nicotinamide adenine dinucleotide phosphate | 1.64 | 3.52 | 0.00 | 0.03 | 0.11 |
| L-Lysine | 1.55 | 4.08 | 0.00 | 0.10 | 0.40 |
| 5-Hydroxylysine | 1.29 | 2.94 | 0.01 | 0.00 | 0.00 |
| 3-Hydroxydecanoic acid | 1.20 | 0.50 | 0.05 | 0.00 | 0.00 |
| Uridine | 1.51 | 6.15 | 0.00 | 0.00 | 0.02 |
| N-Formyl-L-methionine | 0.88 | 2.74 | 0.00 | 0.00 | 0.00 |
| Inosine | 1.48 | 3.87 | 0.01 | 0.01 | 0.04 |
| 3-Hydroxyisobutyric acid | 1.51 | 4.13 | 0.02 | 0.00 | 0.00 |
| L-Sorbose | 1.25 | 3.27 | 0.02 | 0.01 | 0.02 |
| Galactitol | 1.22 | 2.11 | 0.04 | 0.00 | 0.00 |
| Uric acid | 1.36 | 4.36 | 0.01 | 0.00 | 0.00 |
| L-Threonic acid | 1.67 | 1880.07 | 0.02 | 0.00 | 0.00 |
| Cellobiose | 1.58 | 4.32 | 0.02 | 0.04 | 0.18 |
| Gluconolactone | 1.10 | 2.08 | 0.05 | 0.22 | 0.45 |
| Thymidine-5'-phosphate | 1.50 | 5.49 | 0.00 | 0.00 | 0.01 |
| Inositol | 1.50 | 4.46 | 0.01 | 0.16 | 0.72 |
| Trehalose | 1.58 | 3.46 | 0.00 | 0.00 | 0.01 |
| Isomaltose | 1.47 | 3.99 | 0.01 | 0.33 | 1.31 |
| Malonic acid | 1.05 | 4.65 | 0.04 | 0.00 | 0.00 |
| 2-Methylglutaric acid | 1.44 | 14.46 | 0.00 | 0.00 | 0.00 |
| Itaconic acid | 1.54 | 2.82 | 0.00 | 0.00 | 0.00 |
| D-Glucuronate | 1.26 | 1.84 | 0.01 | 0.00 | 0.01 |
| N-Acetyl-L-glutamic acid | 1.31 | 1.62 | 0.02 | 0.00 | 0.00 |
| Succinic acid | 1.57 | 3.11 | 0.00 | 0.01 | 0.02 |
| Dihydroxyacetone phosphate | 1.62 | 0.15 | 0.02 | 2.26 | 0.34 |
| Thymidine-5'-diphosphate | 1.45 | 3.57 | 0.00 | 0.00 | 0.01 |
| Xylulose 5-phosphate | 1.49 | 0.48 | 0.00 | 2.28 | 1.10 |
| N-Acetyl-D-Glucosamine 6-Phosphate | 1.36 | 2.33 | 0.01 | 0.00 | 0.00 |
| 2'-Deoxyguanosine 5'-monophosphate | 1.69 | 2294.43 | 0.01 | 0.00 | 0.00 |
| Ribose-5-Phosphate | 1.41 | 0.50 | 0.02 | 0.05 | 0.02 |
| Cytidine 5'-monophosphate-N-acetylneuraminic acid | 1.26 | 1.84 | 0.02 | 0.01 | 0.01 |
| UDP-D-glucose | 1.20 | 1.56 | 0.04 | 0.05 | 0.07 |
| Fumaric acid | 1.14 | 2.20 | 0.05 | 0.02 | 0.05 |
| Guanosine-5'-monophosphate | 1.27 | 3.28 | 0.01 | 0.01 | 0.03 |
| Deoxyuridine monophosphate (dUMP) | 1.50 | 0.55 | 0.00 | 0.03 | 0.02 |
| N-carbamoyl-L-aspartate | 1.59 | 4.25 | 0.01 | 0.00 | 0.00 |
| Uridine-5'-diphosphate | 1.21 | 1.99 | 0.03 | 0.02 | 0.04 |
| GDP-L-Fucose | 1.32 | 1.69 | 0.02 | 0.00 | 0.01 |
| Glutathione Disulfide | 1.31 | 1.53 | 0.02 | 0.11 | 0.16 |
| Guanosine-5'-diphosphate | 1.42 | 2.98 | 0.01 | 0.02 | 0.07 |

Detected by targeted Metabolomics and UPLC-QQQ-MS analysis.HOS:HCECs cultured in hyp-erosmolar stress(450 mOsM) for 12 hours.HOS+INS:HOS-cultured HCECs treated with 20μg/ml insulin for 12 h.


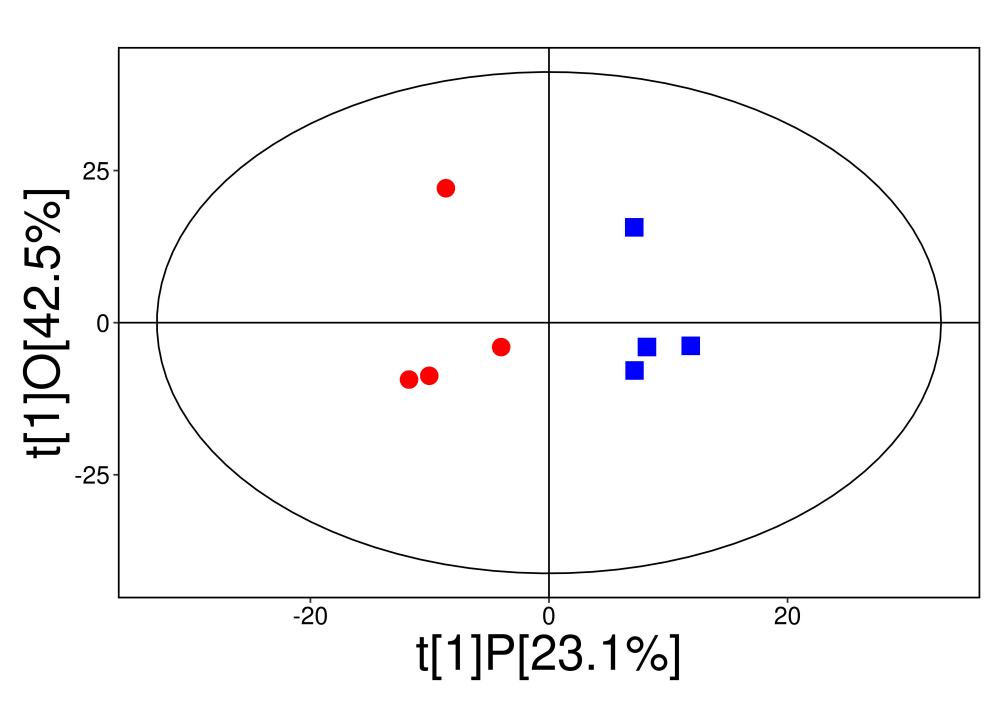


**Figure S11.**Score scatter plot of OPLS-DA model for Normal group and HOS group.Red:Normal group; Blue:HOS group.HOS：HCECs cultured in hyperosmolar stress(450 mOsM) for 12hours.OPLS-DA:orthogonal projections to latent structures- discriminant analysis.


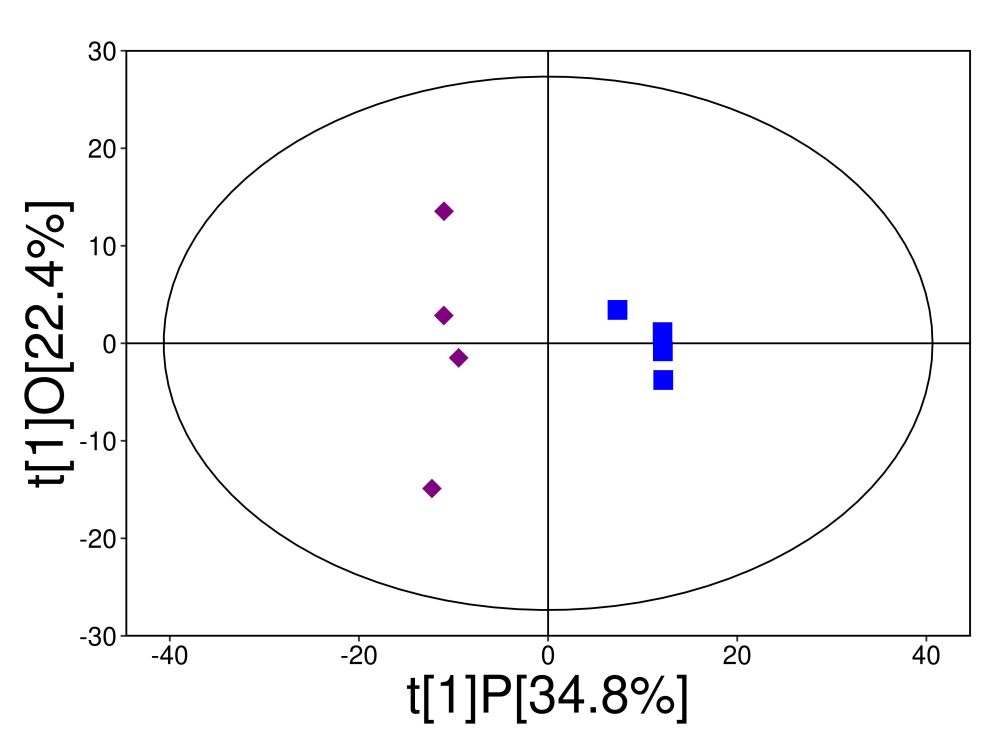


**Figure S12**.Score scatter plot of OPLS-DA model for HOS+INS group and HOS group.Purple:HOS+INS group; Blue:HOS group.HOS：HCECs cultured in hyperosmolar stress(450 mOsM) for 12 hours.HOS+INS：HOS-cultured HCECs treated with 20μg/ml insulin for 12 h.OPLS-DA:orthogonal projections to latent structures- discriminant analysis;


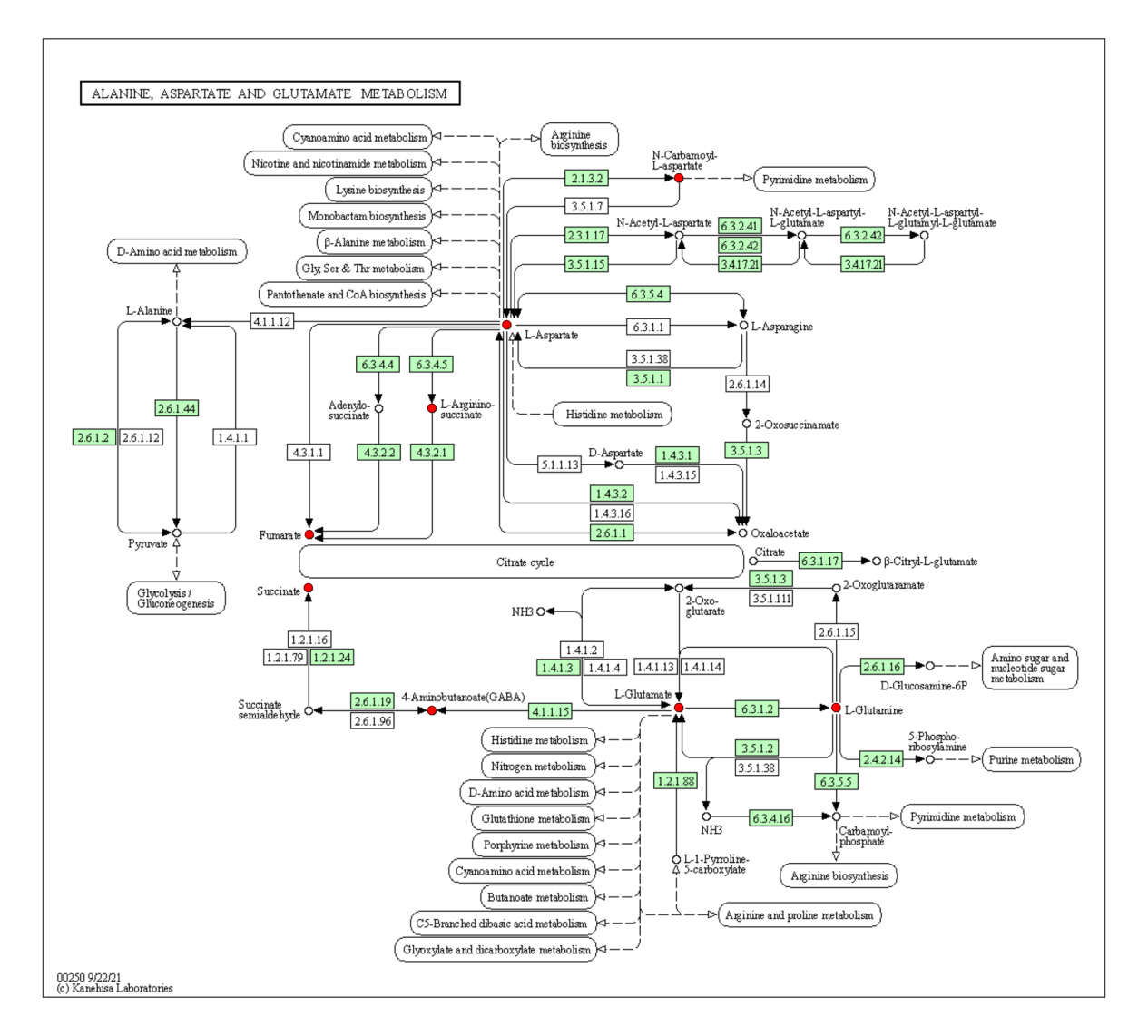


**Figure S13.**KEGG pathways plotted of Alanine,aspartate and glutamate metabolism.Red dots: up-regulated significantly different metabolites; Blue dots: down-regulated significantly different metabolites; Box:the genes (proteins) involved in the pathway;Line:the flow direction of the metabolic reaction.

**Table S6.**Blood routine test results of mice on the 30th day

| Parameters | Concentration | Reference |
| --- | --- | --- |
| ALT (U/L) | 70.38±18.93 | 10.06-96.47 |
| AST (U/L) | 132.17±52.87 | 36.31-235.48 |
| BUN（mg/dL） | 15.09±2.05 | 10.81-34.74 |
| CRE（μmol/L） | 12.63±5.47 | 10.91-85.09 |

PHP-DPS@INS NPs were administered to the eyes of normal Babl/c mice, 2 times a day, 5 μL each time.Values are presented as mean±SD(n=5).

**Table S7.** Biochemical parameters of mice on the 30th day

| Parameters | Concentration | Reference |
| --- | --- | --- |
| WBC（10^9^/L） | 4.39±1.79 | 0.8-6.8 |
| Lymphocyte（10^9^/L） | 2.50±1.16 | 0.7-5.7 |
| Monocyte（10^9^/L） | 0.24±0.18 | 0.0-0.3 |
| RBC（10^12^/L） | 8.60±0.85 | 6.36-9.42 |
| HGB(g/L) | 136.67±5.72 | 110-143 |
| MCHC(g/L) | 308.13±7.18 | 302-353 |
| RDW（%） | 15.75±0.85 | 13-17 |
| PLT（10^9^/L） | 1034.38±272.02 | 450-1590 |
| MPV(fL) | 4.99±0.35 | 3.8-6.0 |

PHP-DPS@INS NPs were administered to the eyes of normal Babl/c mice, 2 times a day, 5 μL each time.Values are presented as mean±SD(n=5).
